# Large-scale plasma proteomics identifies proteins and pathways associated with incident interstitial lung disease

**DOI:** 10.3389/fimmu.2026.1962219

**Published:** 2026-10-21

**Authors:** Zijian Kang, Ying Zhu, Jianzheng Zhang, Chen Zhu, Ping Li, Kai Li, Qiang Tong, Sheng-Ming Dai

**Affiliations:** 1 Department of Rheumatology and Immunology, Shanghai Sixth People’s Hospital Affiliated to Shanghai Jiao Tong University School of Medicine, Shanghai, China; 2 Senior Department of Respiratory and Critical Care Medicine, The Eighth Medical Center of Chinese PLA General Hospital, Beijing, China; 3 Senior Department of Orthopedics, The Fourth Medical Center of Chinese PLA General Hospital, Beijing, China; 4 College of Economics and Management, China Agricultural University, Beijing, China; 5 College of Biological Science and Medical Engineering, Donghua University, Shanghai, China; 6 Department of Rheumatology and Immunology, Beijing Jishuitan Hospital, Capital Medical University, Beijing, China

**Keywords:** biomarkers, inflammation, interstitial lung disease, plasma proteomics, predictive model

## Abstract

**Objective:**

Reliable biomarkers for early interstitial lung disease (ILD) risk stratification are limited; therefore, we aimed to identify prediagnostic proteins associated with incident ILD and develop a long-term prediction model.

**Methods:**

We analyzed 52,743 UK Biobank participants without baseline ILD and measured 2,923 plasma proteins. Cox models were used to identify proteins associated with incident ILD. Key predictors were selected using LASSO and sequential forward selection within a LightGBM framework. Model performance was assessed using the area under the curve (AUC) and SHAP. Prediagnostic protein trajectories were evaluated using LOESS and clustering. Single-cell transcriptomic data from human idiopathic pulmonary fibrosis lungs were used to explore cellular sources.

**Results:**

During a mean follow-up period of 14.4 years, 814 ILD cases were identified in the cohort. A total of 277 proteins were significantly associated with incident ILD and enriched in immune activation and cytokine signaling pathways. Five proteins (GDF15, LAMP3, SFTPD, EDA2R, and WFDC2) were identified as dominant predictors. Trajectory analysis showed that several ILD-associated proteins deviated from control levels years before diagnosis and could be grouped into four distinct temporal clusters. GDF15, LAMP3, SFTPD, WFDC2, and EGFR showed detectable prediagnostic alterations more than a decade before ILD onset. Furthermore, connective tissue disease (CTD)-ILD displayed a distinct inflammatory proteomic profile, including CXCL13, CCL7, PDCD1, and TNFRSF4. When the five protein biomarkers were combined with clinical variables, the integrated model achieved relatively good predictive accuracy for ILD, with AUCs of 0.938 and 0.908 for 5-year and 10-year ILD risks, respectively. Single-cell analysis indicated that these proteins may predominantly originate from dysregulated alveolar and airway epithelial cells.

**Conclusion:**

Plasma proteomics identified prediagnostic protein signatures associated with future ILD risk and improved long-term prediction when combined with clinical variables.

## Introduction

Interstitial lung disease (ILD) is a heterogeneous group of disorders characterized by progressive lung fibrosis and impaired respiratory function, with significant morbidity and mortality (1). The incidence and prevalence of ILD have markedly increased in recent years. In the United States alone, ILD affects approximately 650,000 individuals and is responsible for an estimated 25,000–30,000 deaths annually (1, 2). Idiopathic pulmonary fibrosis (IPF), the most common and severe form, carries a worse prognosis than many malignancies, with a median survival of 3–5 years from diagnosis (3).

While antifibrotic therapies, including nintedanib and pirfenidone, have demonstrated efficacy, their benefits are most pronounced when initiated early, emphasizing the critical need for early detection (4–6). However, the clinical management of ILD remains challenging due to its insidious onset and nonspecific symptoms, which frequently lead to diagnostic delays until a large proportion of patients develop irreversible, end-stage fibrosis (7–9). Current biomarkers for ILD assist in early detection but suffer from low efficacy and an inability to forecast long-term outcomes (10, 11). Pre-symptomatic biomarkers are urgently needed to identify high-risk individuals before structural damage occurs.

Recent advances in high-throughput proteomics have revolutionized biomarker discovery for complex diseases (12, 13). However, comprehensive proteomic profiling for ILD risk prediction in large population-based cohorts remains limited. The UK Biobank, with over 500,000 participants, provides rich proteomic data that may help identify molecular biomarkers associated with ILD (14). The availability of longitudinal data coupled with detailed clinical and lifestyle information makes the UK Biobank an invaluable tool for predictive modeling. When proteomics is linked to prospectively ascertained health records, this technology can transform population biobanks into “molecular observatories” capable of charting protein trajectories years before phenotypic expression. Recent exemplars include the identification of NEFL, CSRP3, and MEGF10 as decade-ahead predictors of amyotrophic lateral sclerosis and the demonstration that cardiac-specific proteins (NT-proBNP and hs-cTnI) forecast heart failure independent of traditional risk factors (15, 16). Therefore, plasma proteomics may help provide an early warning for ILD, enabling therapeutic intervention at the pre-fibrotic stage and offering a realistic prospect of delaying disease onset.

Machine learning algorithms, especially those proficient in high-dimensional data analysis, have shown potential in developing predictive models of disease risk. The Light Gradient Boosting Machine (LightGBM) is a gradient-boosting framework ideal for large, high-dimensional datasets, efficiently managing both categorical and continuous variables (17, 18). By capturing intricate relationships and tolerating missing values, its leaf-wise growth boosts both speed and accuracy, excelling in proteomic data rife with complex protein interactions (19, 20). This method may help synthesize various variables, ranging from genetics to environmental and lifestyle factors, and can discern intricate biological signatures from extensive cohorts, thereby predicting an individual’s risk of developing ILD.

In this study, we focused on identifying plasma proteins significantly associated with the risk of ILD using the UK Biobank Dataset. In addition, we developed a predictive model based on plasma proteins and clinical variables using the LightGBM method. Shapley Additive explanations (SHAP) were used to evaluate the variables within the machine learning model. Our model identifies the risk factors and proteins associated with incident ILD and identifies patients at a higher risk of developing ILD, offering insights into early intervention and improved disease management.

## Methods

### Study population

The UK Biobank is a prospective cohort of 502,357 adults (aged 39–70 years) recruited between 2006 and 2010 through 22 assessment centers embedded within the UK’s National Health Service.

Proteomic measurements were only available for a predefined UKB-PPP subset (21, 22). After excluding 1,040 participants with baseline ILD from 502,357 participants, 501,317 participants remained, of whom 52,743 had proteomic data and were included in the analysis. Follow-up began on the date of the baseline assessment and ended at the earliest incident ILD diagnosis, loss to follow-up, death, or the administrative end of follow-up on August 31, 2023, whichever occurred first.

### Blood proteomics

Fasting EDTA plasma obtained at the baseline visit was centrifuged, aliquoted within 2 h, and stored at −80 °C. Samples were shipped on dry ice to the Olink facility and assayed for 2,923 unique proteins using proximity extension assays under blinded conditions. Normalized protein expression (NPX) values were derived by log2-transforming the ratio of protein-specific counts to internal extension controls, followed by batch and plate-level normalization. Details of quality-control metrics are provided in https://biobank.ndph.ox.ac.uk/ukb/ukb/docs/PPP_Phase_1_QC_dataset_companion_doc.pdf.

### Clinical covariates

Demographic, lifestyle, anthropometric, and morbidity data were extracted from baseline touchscreen questionnaires, physical measurements and electronic health records. Covariates were selected *a priori* based on previously reported associations with ILD, including age, sex, ethnicity, body mass index (BMI), Townsend deprivation index, smoking status, alcohol intake, physical activity, hypertension, type 2 diabetes, and air pollution. To assess the robustness of the protein-ILD associations with additional clinical factors, we performed sensitivity analyses with further adjustment for baseline gastroesophageal reflux disease (GERD), substance-related disorders, and systemic corticosteroid use. FEV1 and FVC were measured at baseline, and percent-predicted values were calculated using reference equations incorporating age, sex, and height. These measures reflect baseline lung function rather than ILD severity at the time of subsequent diagnosis. Because baseline lung function measures were correlated, FEV1% predicted and FVC % predicted, calculated using the study reference equations, were additionally incorporated into separate models to minimize potential collinearity. Detailed definitions of all covariates are provided in **Supplementary Table 1**.

### ILD outcomes

Incident ILD was ascertained from UK Biobank first-occurrence records derived from linked primary care, hospital inpatient, and death-registration data using International Classification of Diseases, 10th Revision (ICD-10) codes (23). The selected diagnostic codes were informed by previously published population-based studies using routinely collected healthcare data (23–25). Cases of connective tissue disease-associated ILD (CTD-ILD) required a qualifying CTD diagnosis before the ILD index date, including rheumatoid arthritis, systemic sclerosis, systemic lupus erythematosus, dermatomyositis, and Sjögren’s syndrome. The qualifying ICD-10 codes are listed in **Supplementary Table 2**. Case ascertainment was further assessed by data source. A sensitivity analysis used a more restrictive definition based only on hospital inpatient records and death registrations (**Supplementary Figure 1**).

### Single-cell type expression analysis

To assess the cell type–specific expression of ILD-associated protein-coding genes, we analyzed a publicly available single-cell RNA-seq dataset of human IPF (GSE135893), (26), which included 20 IPF patients and 9 healthy controls. The raw expression matrix was processed using the Seurat package. We filtered out genes detected in fewer than three cells and those with fewer than 200 unique features. The counts were normalized and scaled using the NormalizeData and ScaleData functions. Dimensionality reduction (principal component analysis), visualization (uniform manifold approximation and projection, UMAP), and graph-based clustering were applied to the integrated Seurat object. Cell types were annotated using known marker genes. Differential expression between a given cell type and all other cells was evaluated using the Wilcoxon rank-sum test to identify differentially expressed genes.

### Statistical analysis

Baseline characteristics were compared using the χ² test for categorical variables. For continuous variables, two-sample t-tests or Wilcoxon rank-sum tests were used, depending on the distribution of the data. Protein-outcome associations were estimated using Cox proportional hazards regression, reporting hazard ratios (HRs) and 95% confidence intervals (CIs) per 1-standard-deviation increase in NPX. Two models were fitted: Model 1 was adjusted for age, sex, body mass index, ethnicity, and Townsend deprivation index; Model 2 was additionally adjusted for smoking status, alcohol intake, physical activity, hypertension, type 2 diabetes, cardiovascular disease, and exposure to air pollution. Sensitivity analyses were further adjusted for baseline GERD, substance-related disorders, and systemic corticosteroid use and lung function (FEV1 and FVC percentage predicted).

To compare the leading proteins across different stages of ILD, we further performed propensity-score matching among participants with prevalent ILD at baseline, those who subsequently developed incident ILD, and those without recorded ILD during follow-up. Matching incorporated age, sex, ethnicity, smoking status, BMI, major comorbidities, medication use, and baseline lung function. Baseline levels of proteins were compared across the three matched groups, with Bonferroni correction applied for multiple comparisons.

To reduce the potential influence of clinically unrecognized ILD present at baseline, we additionally conducted 2- and 5-year landmark analyses. Participants who developed ILD before each landmark were excluded, and associations with subsequent ILD were reassessed among participants who remained free of recorded ILD and under follow-up at the landmark. Cox models incorporated the expanded clinical covariates, with FEV1% predicted and FVC % predicted evaluated in separate models. Bonferroni correction was applied for testing of the five proteins.

To guard against false positives across the 2,923 proteins, we applied the Bonferroni correction (α = 0.05/2923). Proteins that survived this threshold in both models were subjected to over-representation analysis using ClusterProfiler (false discovery rate [FDR] < 0.05) against the entire Olink panel as a background.

Bonferroni-significant proteins were filtered using least absolute shrinkage and selection operator (LASSO)-penalized Cox regression. The retained candidates were forwarded to a gradient-boosting framework (LightGBM) coupled with sequential forward selection (SFS). Feature importance was ranked using SHAP values. Incremental addition was stopped when it failed to improve the area under the receiver operating characteristic (ROC) curve (AUC). Hyperparameters (learning rate, tree depth, and number of leaves) were optimized using Bayesian tuning under leave-one-center-out cross-validation (five centers). Model performance was summarized as AUC, sensitivity, specificity, and accuracy with 1000-bootstrap 95% CIs.

To test the generalizability of the pipeline, we repeated the pipeline in three prediction horizons: ILD diagnosed ≤ 5 years, ≤ 10 years, and all incident cases. Kaplan–Meier curves were constructed after dividing the participants according to the optimal NPX cutoff point of the Youden index. The incidence rates were also calculated across the protein expression tertiles.

Proteins selected from the LASSO-penalized Cox regression model were included in trajectory analysis (27, 28). Extreme protein values were winsorized at the 2.5th and 97.5th percentiles. To balance the baseline characteristics between ILD cases and controls at each time interval before diagnosis, propensity score matching was performed using age, sex, and BMI as covariates (1:5 case-to-control ratio, caliper = 0.01). Within each matched set, the protein levels in the cases were standardized to Z-scores using the means and standard deviations (SMD) of the matched controls. The Z-score trajectories across the years before diagnosis were smoothed using locally estimated scatterplot smoothing (LOESS). Proteins with absolute smoothed Z-scores below 0.05 were considered unchanged. Hierarchical clustering with Ward’s minimum variance method was applied to the smoothed trajectory matrix, and the proteins were grouped into four clusters. Functional enrichment analysis for each cluster was performed using the Gene Ontology (Biological Process, Cellular Component, and Molecular Function), KEGG, and Reactome pathway databases, with Benjamini-Hochberg correction (adjusted *P* < 0.05 considered significant).

All analyses were performed using R 4.3.1. Two-sided P-values < 0.05 were considered significant.

## Results

### Participant selection and baseline characteristics

After excluding 1,040 participants with baseline ILD and 448,574 without proteomic data, 52,743 of 502,357 UK Biobank participants were included (**Figure 1A**). Participants with and without proteomic data had similar baseline characteristics (all SMDs <0.05), although ILD incidence was higher in the proteomic subset (**Supplementary Tables 3**, **4**). In the analytic cohort, most participants were white (93.1%) and had a median age of 58 years. Among them, 54% (28,464) were female and 46% (24,279) were male (**Supplementary Table 5**). Over a mean follow-up period of 14.42 years, 814 patients were diagnosed with ILD during the study period, with 199 and 401 cases occurring within 5 and 10 years, respectively (**Figures 1A–C**).

**Figure 1:**
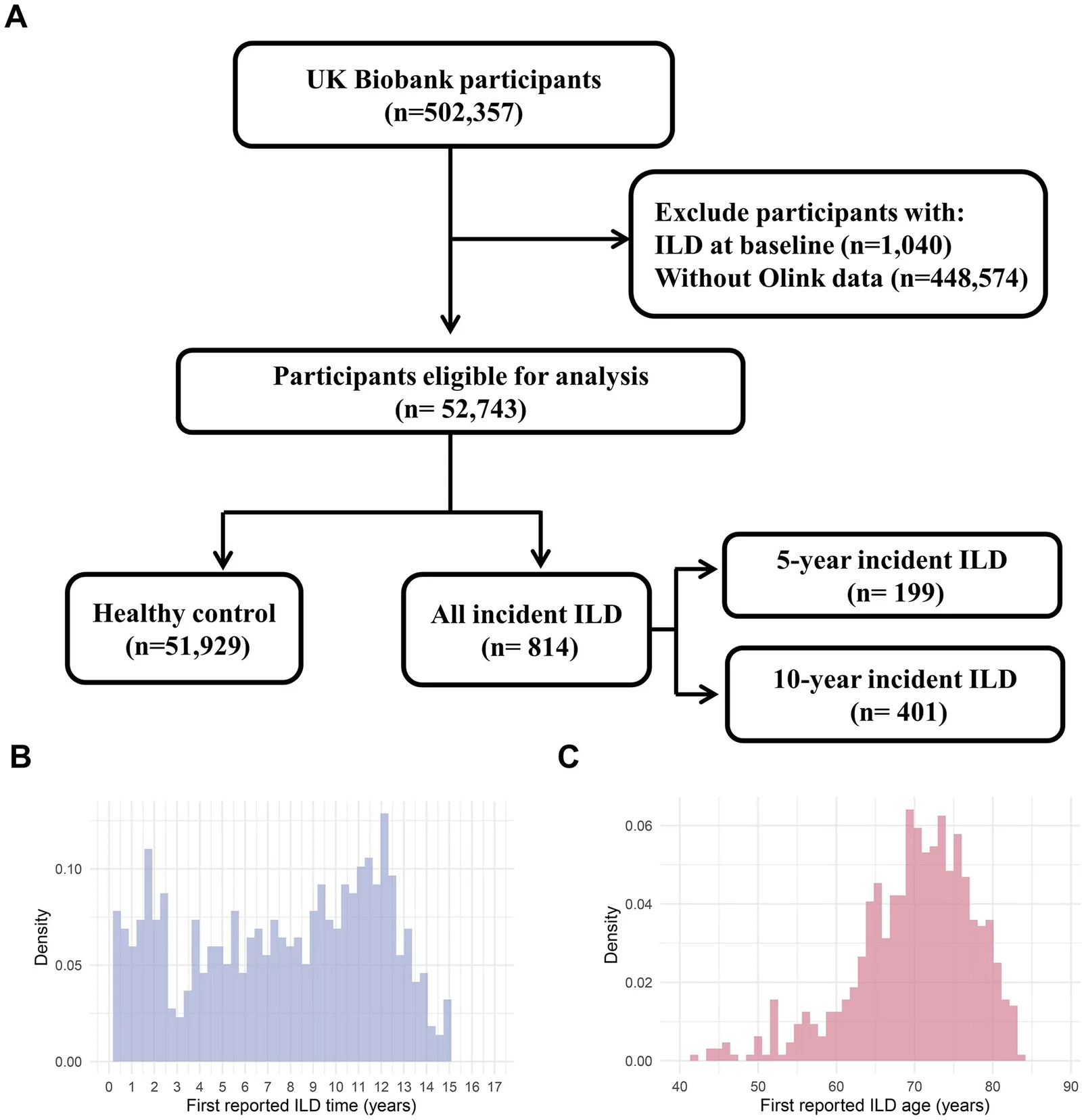
Participant selection flowchart. **(A)** In the UK Biobank cohort, participants who have ILD at baseline or lacked olink data. proteomic data were excluded. The remaining individuals were followed from baseline to the earliest incident ILD diagnosis, death, loss to follow-up, or censoring at the end of follow-up. **(B, C)** Distribution of the observation period for the first reported ILD **(B)** and age at first ILD record **(C)**. ILD, Interstitial lung disease.

Compared with participants who did not develop ILD, those with incident ILD were older at recruitment (median age, 64 vs. 58 years) and less frequently female (44.2% vs. 54.1%). They were more frequently obese and former or current smokers, lived in more deprived areas, and had lower physical activity and higher air pollution scores. They also had lower baseline FVC or FEV1 percentage predicted and FEV1/FVC values, higher prevalence of hypertension, diabetes, cardiovascular disease, GERD, and substance-related disorders, and more frequent use of systemic corticosteroids and ILD-associated medications (all P<0.001; **Supplementary Table 5**).

### Identifying proteins associated with incident ILD

To identify the proteins truly associated with ILD outcomes, we used Cox regression analysis and adjusted for known clinical factors to control for potential confounding effects. Our comprehensive analysis of 2,923 proteomic biomarkers revealed a significant association between a subset of proteins and incident ILD (**Figure 2**). After adjusting for age, sex, BMI, Townsend index, and ethnicity in Model 1, a total of 440 proteins were found to be significantly correlated with new-onset ILD after Bonferroni correction (**Figure 2A**). As a sensitivity analysis, we re-ran all analyses using Model 2, which was additionally adjusted for smoking status, drinking status, daily activity, comorbidities, and air pollution. A total of 277 proteins remained significantly associated with the development of new-onset ILD (**Figures 2A, B**). For example, SCGB3A1 and ROBO2 were positively associated with incident ILD, with HRs of 9.85 (95% CI:6.99-13.88) and 7.89 (95% CI: 5.77-10.78), respectively. In contrast, EGFR and CLEC3B exhibited HRs of 0.14 (95% CI: 0.09–0.22) and 0.21 (95% CI: 0.13–0.34), respectively, suggesting a negative correlation with ILD risk (**Supplementary Table 6**). The associations of the top-ranked proteins remained robust under a more restrictive hospital-based case definition (**Supplementary Figure 2**). Further adjustment for GERD, substance-related disorders, systemic corticosteroid use, and baseline lung function had limited effects on the association between the top-ranked proteins and incident ILD. Across the leading proteins identified in the discovery analysis, the effect estimates were generally consistent with those from the primary model, with only modest attenuation after additional clinical adjustment. Similar results were observed when FEV1% predicted and FVC % predicted were incorporated separately into the models (**Supplementary Figure 3**).

**Figure 2:**
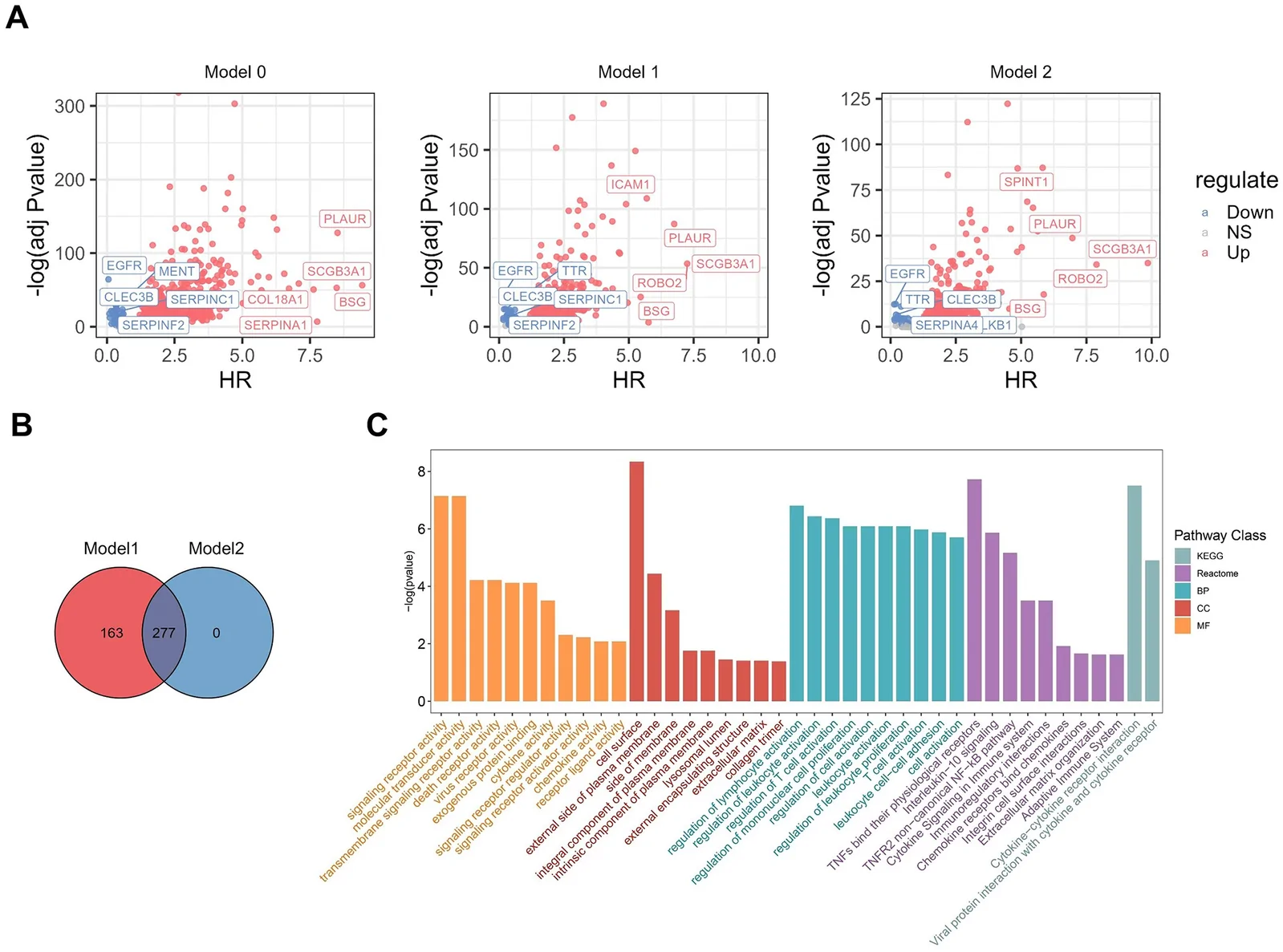
Association of plasma proteins with incident ILD. **(A)** Volcano plot showing the association of 2,923 plasma proteins with incident ILD in terms of HRs on the x-axis and -log (FDR) on the y-axis. Cox proportional hazards regression in Model 0 (left), Model 1(middle), and Model 2 (right) results are presented here. Model 0 is the crude model. Model 1 is adjusted for age, sex, BMI, ethnicity, and Townsend index. Model 2 included additional adjustments for daily activities, smoking, alcohol consumption, hypertension, diabetes, and air pollution. **(B)** Venn diagram showing the proteins shared between Models 1 and 2. **(C)** Enrichment of GO, KEGG, and Reactome pathways. Significant proteins identified in both models after Bonferroni correction were subjected to enrichment analysis using the Olink protein panel as a background gene set. FDR, False discovery rate; HR, hazard ratio; BMI, Body mass index; GO, Gene ontology; KEGG, Kyoto encyclopedia of genes and genomes.

Enrichment analysis revealed several biological pathways associated with the significantly correlated proteins, including T-cell migration and activation, cytokine signaling, TNF signaling, and NF-κB pathways (**Figure 2C**; **Supplementary Table 7**). STRING functional network analysis placed GDF15, LAMP3, and SFTPD within the largest connected component, whereas EDA2R and WFDC2 had no retained connections at the selected confidence thresholds (**Supplementary Figure 4A**). Module-level enrichment highlighted cytokine-mediated signaling, extracellular matrix organization, and TNFR2 non-canonical NF-κB signaling as the most significant annotations (**Supplementary Figure 4B**). These findings may provide insights into the molecular mechanisms underlying the development of ILD.

### Protein importance ranking via SHAP analysis

To identify the most informative proteins among the many candidates associated with incident ILD, we constructed a multivariate prediction model. First, LASSO regularization was applied to the complete panel of 277 circulating proteins to remove redundant features and mitigate multicollinearity (**Supplementary Figures 5A, B**). The 162 proteins retained after LASSO were then subjected to SFS to isolate the smallest set that maximized AUC (**Figure 3A**). The AUC increased steeply with the first few additions and then levelled off. GDF15 and LAMP3 were the strongest individual contributors, followed by SFTPD, EDA2R, and WFDC2 (**Figure 3A**).

**Figure 3:**
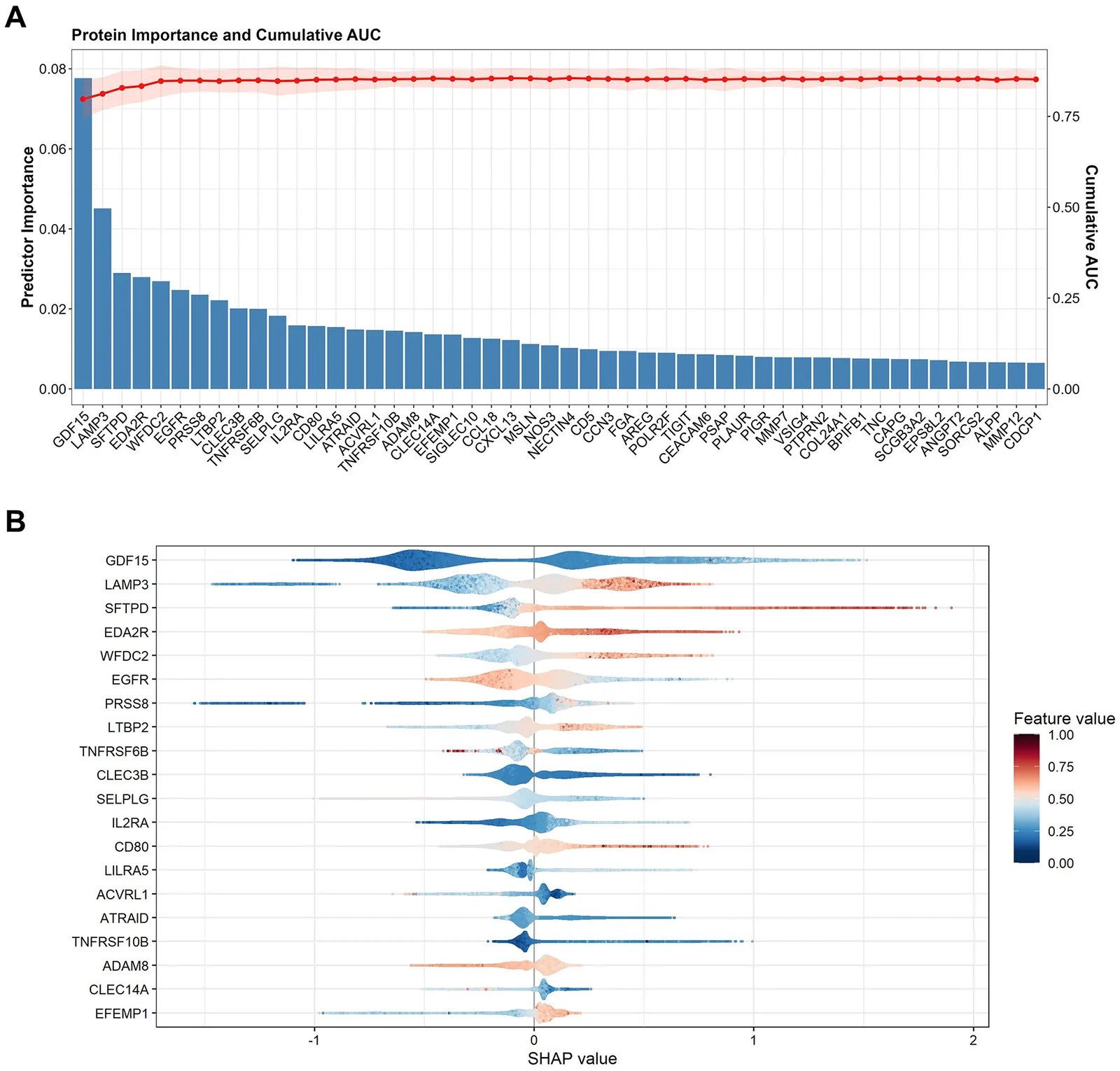
Protein importance ranking and SHAP visualization for all incident ILD. **(A)** Sequential forward selection from a pool of candidate proteins. Feature importance was calculated as the mean absolute SHAP value for each predictor, normalized by the sum of mean absolute SHAP values across all predictors within each fold, and then averaged across the five folds. Higher values indicate greater relative predictive contribution within the corresponding model. The line graph shows each iteration’s cumulative AUC values (right y-axis) as proteins are included individually. The top selected proteins are highlighted in red. The shaded area represents the standard error. **(B)** SHAP visualization of the top proteins. The width of the horizontal bars can be interpreted as the degree of contribution to the prediction of ILD; the wider the range, the more significant the contribution. The color of the horizontal bars indicates the magnitude of the plasma proteins encoded in a gradient from dark blue (low) to red (high), as shown in the color bar on the right side of the figure. The x-axis represents the likelihood of developing ILD (right side) or remaining healthy (left side). SHAP, SHapley Additive exPlanations; AUC, Area under the curve.

To quantify the direction and magnitude of each protein’s contribution, we computed the SHAP values for each participant, which assigned each feature an importance value that reflected its contribution to the model output. The SHAP beeswarm plots illustrate feature importance through color gradients representing value magnitude and horizontal positioning, indicating the direction and degree of influence on the probability of developing ILD (**Figure 3B**; **Supplementary Figures 6A–C**). Our results showed that higher circulating levels of GDF15, LAMP3, and SFTPD shifted the model output toward a higher probability of ILD, with GDF15 exhibiting the widest SHAP value range, underscoring its predominant effect. Conversely, elevated EGFR and TNFRSF6B expression levels were associated with negative SHAP scores, indicating a protective effect. The Kaplan–Meier curves showed consistent patterns, with a higher cumulative incidence of ILD in participants with elevated risk-associated proteins and a lower incidence in those with higher EGFR levels (**Supplementary Figure 7**).

### Dose-response associations of candidate proteins

To further characterize the most informative proteins associated with incident ILD, we examined their risk gradients, dose–response relationships, and associations with prevalent ILD. We also found that participants with higher baseline protein percentiles had higher event rates than those with lower protein percentiles (**Supplementary Figure 8A**). This association demonstrates the potential of plasma proteins as stratification biomarkers for the risk of ILD. To elucidate the dose–response relationship between top protein expression levels and ILD risk, we performed restricted cubic spline (RCS) analyses. Notably, several proteins exhibited non-linear associations with ILD risk. Specifically, GDF15, SFTPD, EDA2R, and WFDC2 exhibited J-shaped curves, indicating markedly increased risk at higher concentrations. In contrast, EGFR displayed a reverse J-shaped curve, with a more pronounced protective effect at elevated levels (**Supplementary Figure 8B**). Together, these analyses provide additional support for the robustness and relevance of the top-ranked protein signals associated with incident ILD.

### Comparison of prevalent and future incident ILD and time-lagged landmark analyses

To further examine whether the identified proteins reflected established disease or were also detectable before incident ILD, we first assessed their associations with prevalent ILD. All five candidate proteins showed higher circulating levels in participants with prevalent ILD than in those without (**Supplementary Figure 9A**), and these associations remained significant in both age- and sex-adjusted and fully adjusted logistic regression models (**Supplementary Figure 9B**).

We then compared baseline protein levels across propensity score-matched participants with prevalent ILD, future incident ILD, and no recorded ILD (**Supplementary Table 8**). All five proteins were elevated in both the prevalent and future incident ILD groups compared with participants without recorded ILD. The levels of WFDC2 were higher in prevalent ILD than in future incident ILD, whereas those of GDF15, LAMP3, SFTPD, and EDA2R did not differ significantly between the two ILD groups after Bonferroni correction (**Supplementary Figure 10A**).

In the 2- and 5-year landmark analyses, excluding participants who developed ILD before each landmark, all five proteins remained significantly associated with subsequent ILD after expanded clinical adjustment, including separate adjustment for FEV1% predicted or FVC % predicted, and after Bonferroni correction for the five proteins (**Supplementary Figure 10B**). These findings indicate that the associated proteomic alterations were detectable several years before the first recorded ILD diagnosis, although they cannot distinguish true preclinical changes from subclinical or clinically unrecognized disease already present at baseline.

### Temporal evolution and trajectory clustering of ILD-associated proteins before ILD diagnosis

We used LOESS smoothing to characterize temporal changes in plasma proteins before ILD diagnosis. The ILD-associated proteins were grouped into four trajectory clusters with distinct temporal patterns (**Figures 4A, B**). Among the five leading biomarkers, GDF15, LAMP3, and WFDC2 belonged to Cluster 2 and showed progressive increases toward diagnosis, whereas EDA2R and SFTPD belonged to Cluster 4 and increased after an initially stable or slightly decreased phase. These trajectory patterns provide temporal context for the ILD-associated proteins. The corresponding functional interpretations of the four clusters are shown in **Supplementary Figure 11**. Cluster 1 was enriched in caspase activation, cytokine–cytokine receptor interaction, TNF signaling, and myeloid leukocyte activation. Cluster 2 was enriched in neutrophil degranulation, extracellular matrix organization, elastic fiber formation, cytokine activity, lysosomal components, and cell-adhesion-related pathways. Cluster 3 showed enrichment in EGFR and PI3K-Akt signaling, interferon-γ response, immune regulation, cytokine–cytokine receptor interaction, and leukocyte adhesion. Cluster 4 was predominantly related to extracellular matrix organization, integrin and cytokine receptor interactions, IL-10 and TLR-related signaling, and T-cell or lymphocyte proliferation. Together, these findings indicate that prediagnostic ILD is accompanied by distinct proteomic trajectories involving immune activation, extracellular matrix remodeling, epithelial or growth-factor signaling, and inflammatory responses.

**Figure 4:**
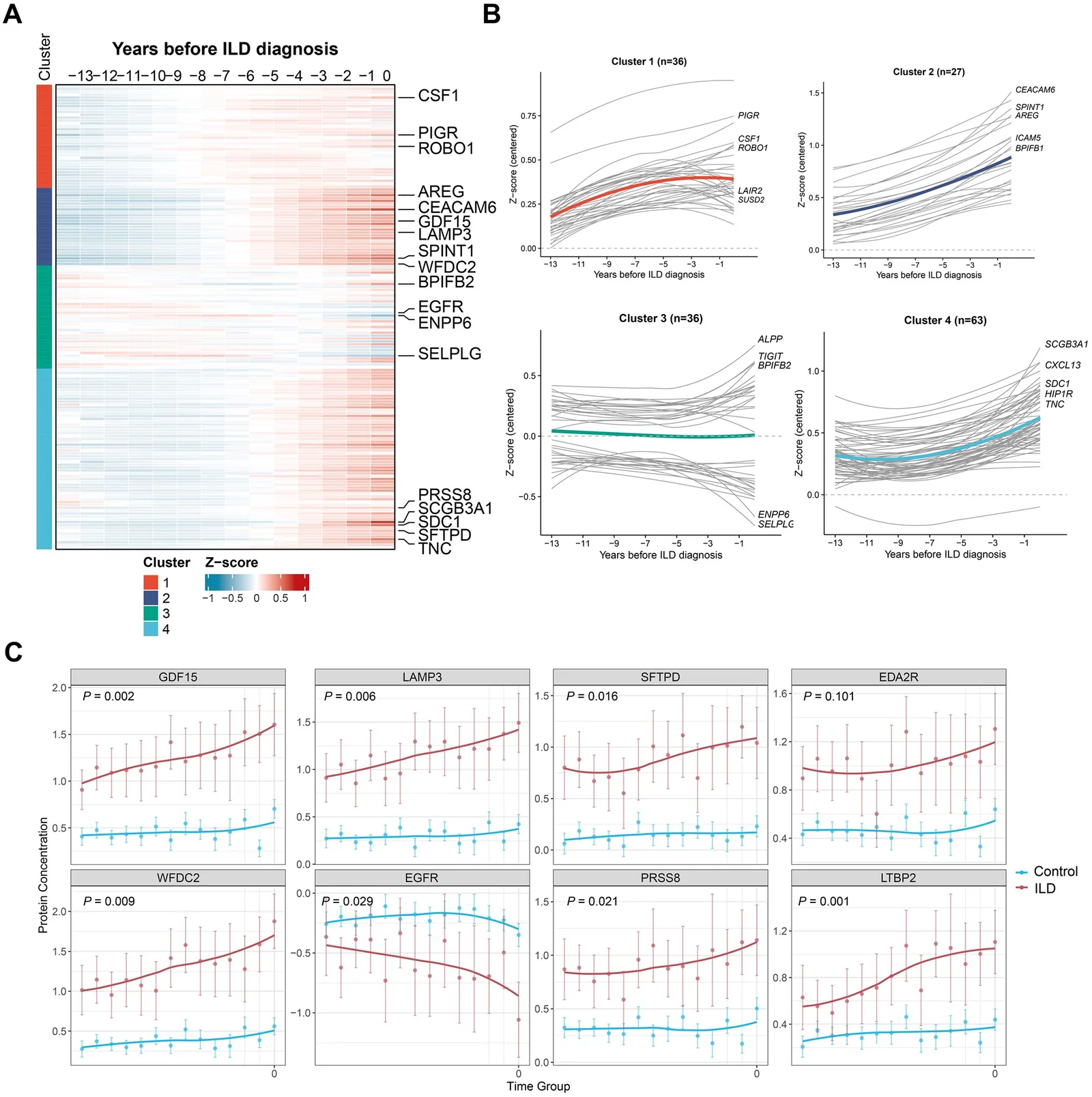
Temporal evolutions of plasma proteins before the diagnosis of ILD and their trajectory clustering. **(A)** Changes in the Z-scores of ILD-associated proteins over time. Protein levels were standardized using the mean and standard deviation of the corresponding plasma proteins in matched controls as the reference. The trajectories were fitted using LOESS smoothing. Colors indicate absolute Z scores greater than 0.25, with red representing positive Z scores and blue representing negative Z scores. **(B)** Trajectories of proteins within the four clusters. Proteins were grouped using unsupervised hierarchical clustering. Thin lines represent individual protein trajectories, and thick lines indicate the mean trajectories within each cluster. **(C)** Dynamic changes in plasma levels of the top eight genes before the onset of ILD are illustrated. A nested case-control study was used to match individuals who experienced events within a 14-year observational period with healthy controls matched for covariates in Model 1. Red curves correspond to individuals who developed ILD, whereas blue curves represent the control group. Protein concentrations averaged over time to the index date were fitted using locally weighted smooth curves. Error bars indicate the standard error. The Mann–Kendall trend test was performed to assess the difference in the slope of plasma protein levels over time between individuals who developed ILD and those who did not, with P values calculated using a two-tailed test without adjustment for multiple comparisons. ILD, interstitial lung disease; LOESS, locally estimated scatterplot smoothing.

Next, we examined the temporal changes in the top SHAP-ranked proteins during the 14 years preceding the ILD diagnosis. Compared with matched controls who remained disease-free over the same period, individuals who later developed ILD showed early divergence in plasma GDF15, LAMP3, SFTPD, and WFDC2 levels, beginning more than 10 years before diagnosis, whereas these proteins remained largely stable in the controls (**Figure 4C**). EGFR showed the opposite pattern, with a gradual prediagnostic decline in the ILD group. In contrast, EDA2R did not show any clear dynamic changes before diagnosis (**Figure 4C**).

Collectively, these findings indicate that alterations in plasma GDF15, LAMP3, SFTPD, WFDC2, and EGFR levels are detectable more than a decade before the clinical manifestation of ILD, suggesting their potential roles as early biomarkers for disease risk stratification.

### Distinct plasma protein profiles between CTD-ILD and non-CTD ILD

As CTD-ILD represents a clinically major phenotypic subset of ILD, we investigated whether its circulating proteome differs from that of non-CTD ILD. Of the 277 ILD-associated plasma proteins analyzed, 25 showed differential abundance, defined by an FDR < 0.05 and an absolute log2 fold-change>0.2. Compared with non-CTD ILD, patients with CTD-ILD exhibited significant upregulation of proteins involved in immune activation and inflammatory responses, including CXCL13, CCL7, PDCD1, TNFRSF4, and RRM2, whereas SFTPD was notably downregulated (**Figures 5A, B**; **Supplementary Table 9**).

**Figure 5:**
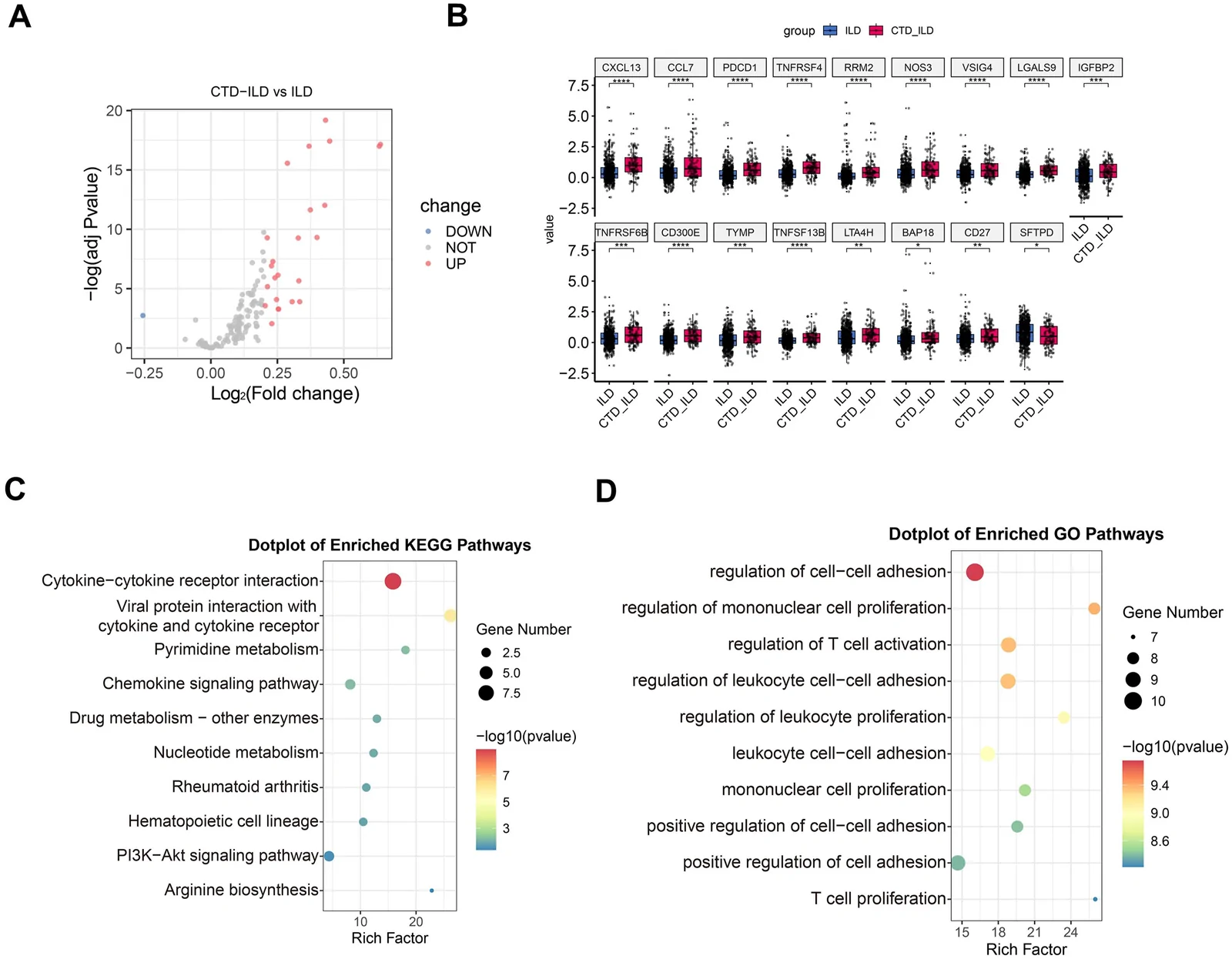
Differential expression and pathway enrichment in CTD-ILD. **(A, B)** Volcano plot **(A)** and box plots **(B)** showing the plasma proteins that were significantly differentially expressed between CTD-ILD and non-CTD ILDs. **(C, D)** GO and KEGG pathway enrichment analyses of significant proteins between CTD-ILD and non-CTD ILDs. Connective tissue disease. *P < 0.05, **P < 0.01, ***P < 0.001, and ****P < 0.0001.

Functional enrichment analysis revealed that these differentially expressed proteins were significantly enriched in several key pathways, including “cytokine–cytokine receptor interaction”, “chemokine signaling pathway”, and Gene Ontology terms related to mononuclear cell proliferation and T-cell activation (adjusted P < 0.005) (**Figures 5C, D**).

These findings suggest that CTD-ILD is characterized by a heightened and chronically activated adaptive immune signature detectable in the peripheral blood, providing a molecular basis for the inflammatory clinical behavior observed in this ILD subtype.

### Clinical Feature selection and interpretability analysis using SHAP

To comprehensively evaluate the predictors of ILD risk, we initially incorporated approximately 150 clinical variables, including demographic characteristics, lifestyle habits, socioeconomic factors, physical activity status, biochemical indicators, and air pollution metrics. An initial screening process using LASSO regression identified a subset of candidate predictors, most of which have been previously reported to be associated with the risk of ILD (**Supplementary Figures 12A, B**). The top 50 features selected by SFS were further analyzed and ranked according to their predictive contributions (**Figure 6A**). Several strong correlations were observed among the predictive factors, including those between age, illness history, smoking status, and air pollution exposure levels.

**Figure 6:**
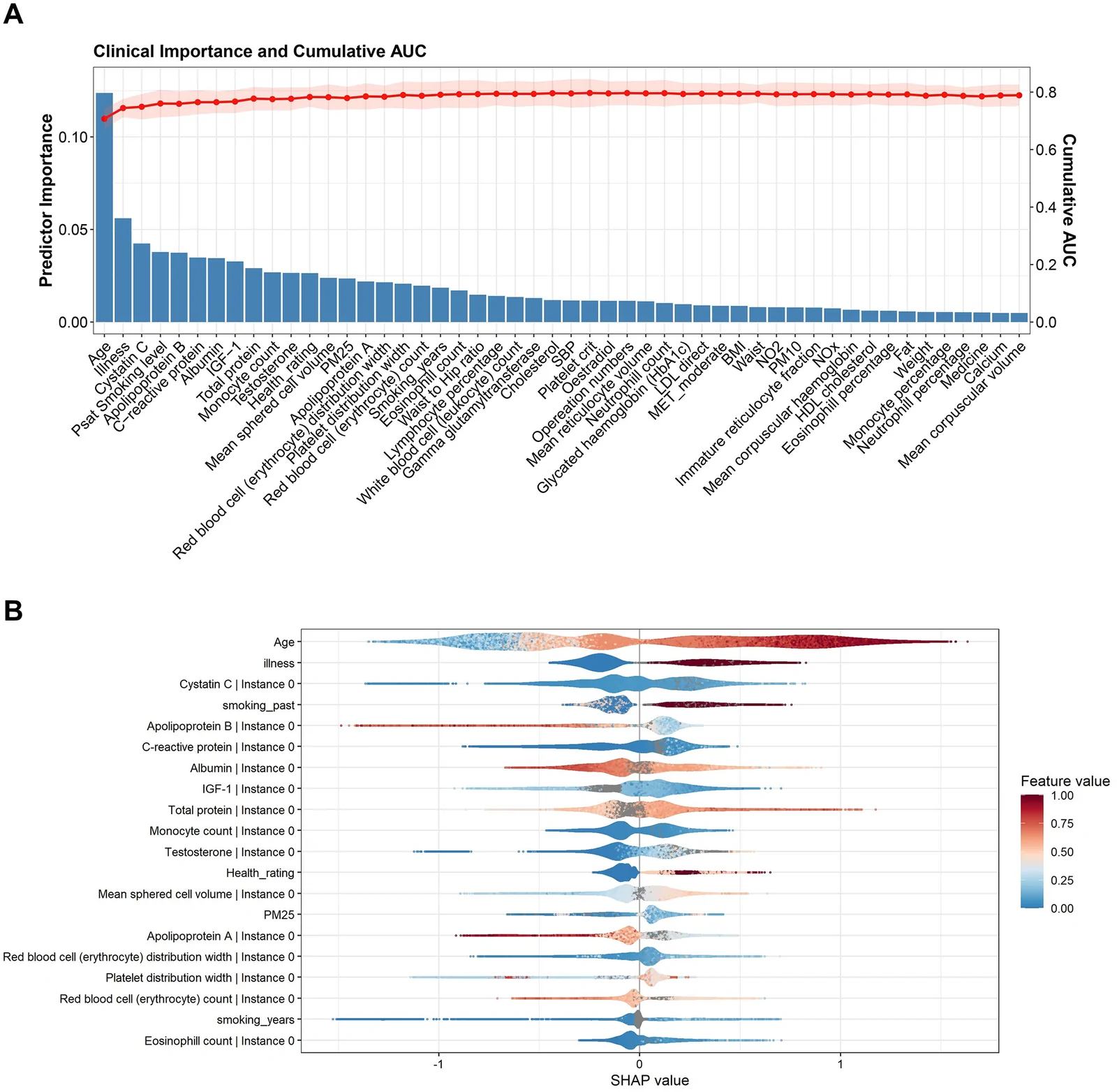
Clinical variable importance ranking and SHAP visualization for incident ILD. **(A)** Sequential forward selection from a pool of candidate clinical variables. The bar chart illustrates the importance of the ranked clinical variables based on their contribution to predicting future ILD, as judged by the SHAP values. The line graph shows each iteration’s cumulative AUC values (right y-axis) as proteins are included individually. The top selected clinical variables are highlighted in red. The shaded area represents the standard error. **(B)** SHAP visualization of the top clinical variables. The width of the horizontal bars can be interpreted as the degree of contribution to the prediction of ILD; the wider the range, the greater the contribution. The color of the horizontal bars indicates the magnitude of the clinical variables encoded in a gradient from dark blue (low) to red (high), as shown in the color bar on the right. The gray color indicates missing values. The x-axis represents the likelihood of developing ILD (right) or remaining healthy (left). AUC, area under the curve; SHAP, SHapley Additive exPlanations; ILD, interstitial lung disease; BMI, body mass index; PM2.5, particulate matter with an aerodynamic diameter ≤2.5 μm.

SHAP value analysis demonstrated that age exhibited the broadest variation in impact among all patients with new-onset ILD, followed by illness history, cystatin C, past smoking intensity, apolipoprotein B (APOB), C-reactive protein, albumin, and insulin-like growth factor 1 (IGF1) (**Figure 6B**; **Supplementary Figures 13A–C**). Other notable contributors included exposure to fine particulate matter (PM2.5), lymphocyte count, and monocyte count. This finding suggests that the risk of ILD is multifactorial, influenced by a complex interplay of demographic, clinical, inflammatory, and environmental factors that can be quantitatively prioritized using SHAP-based interpretability methods.

### Predictive accuracy of ILD-risk model

To construct a high-performing risk prediction model for ILD, we integrated plasma protein biomarkers with clinical variables to enhance the predictive accuracy for future ILD risk. Feature selection was based on SFS analysis, with the process halted when the incremental increase in the AUC fell below 0.01, resulting in the final inclusion of five proteins and five clinical variables in the optimized model.

The model performance was evaluated across multiple time horizons. Using proteins alone, the AUC values were 0.899 for 5-year ILD prediction, 0.890 for 10-year ILD prediction, and 0.859 for all-incident ILD detection (**Figures 7A–C**). In comparison, models based solely on clinical variables achieved AUCs of 0.847, 0.796, and 0.784 for the same timeframes, respectively (**Figures 7A–C**). The integration of plasma proteins with clinical factors yielded a substantial improvement in discriminative ability, with combined AUCs rising to 0.938, 0.908, and 0.891 for the 5-year, 10-year, and all-incident ILD predictions, respectively (**Figures 7A–C**). Concurrently, the integration of plasma proteins with clinical variables also increased sensitivity and specificity across all time horizons (**Supplementary Table 10**).

**Figure 7:**
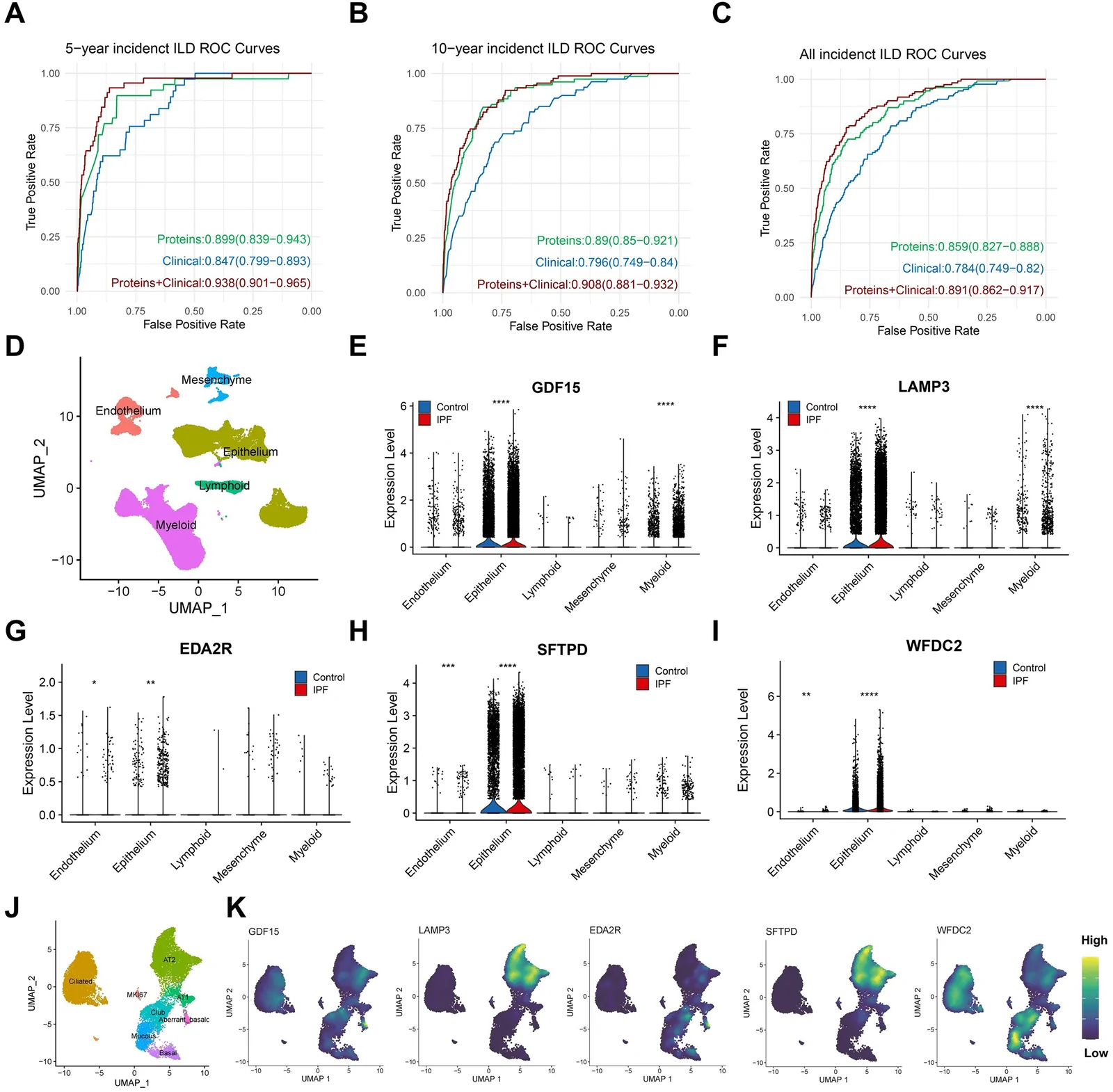
Predictive accuracy of protein biomarkers for incident ILD and validation of their gene expression in single-cell pulmonary data. **(A–C)** ROC curves illustrating the predictive performance of the proteins, clinical variables, and combination of these factors in identifying 5-year incident ILD **(A)**, 10-year incident ILD **(B)**, and all incident ILD **(C)**. **(D)** UMAP plot showing the single-cell transcriptomic atlas of IPF. **(E–I)** Violin plots showing the expression levels of five biomarkers between the IPF and control groups. **(J)** UMAP plot showing the pulmonary epithelial cell subpopulations. **(K)** UMAP plot showing the expression levels of the five biomarkers in epithelial subsets. The expression levels were overlaid onto the cell clusters. ROC, Receiver operating characteristic; IPF, Idiopathic pulmonary fibrosis; UMAP, Uniform manifold approximation and projection. *P < 0.05, **P < 0.01, ***P < 0.001, and ****P < 0.0001.

### Single-cell type expression analysis of candidate protein-coding genes

To evaluate the cell type-specific enrichment of five candidate protein-coding genes in lung tissue and their differential expression between patients with IPF and healthy controls, we performed single-cell-type expression analysis using a published single-cell RNA-seq dataset of human IPF. Cells were clustered into five major types: epithelial, immune, stromal, endothelial, and myeloid cells (**Figure 7D**; **Supplementary Figures 14A, B**). The five biomarker-encoding genes were predominantly expressed in epithelial cells. Moreover, their expression levels in epithelial cells from patients with IPF were significantly higher than those in healthy controls (**Figures 7E–I**).

To further characterize the epithelial populations exhibiting prominent expression, we subclustered the epithelial cells into distinct subsets, including ciliated, MKI67^+^, alveolar type 1 (AT1), alveolar type 2 (AT2), club, basal, and mucous cells (**Figure 7J**). Among these, GDF15 was primarily expressed in AT2 and ciliated cells; LAMP3 and SFTPD were largely restricted to AT2 cells; EDA2R was notably enriched in abnormal basal cells; and WFDC2 was mainly detected in mucous, ciliated, and club cells (**Figure 7K**). Therefore, these results delineate the precise epithelial subpopulations that contribute to the elevated expression of the five candidate biomarkers in IPF, underscoring the alveolar and airway epithelial compartments as key sites of dysregulation in this disease.

## Discussion

In this large prospective cohort study, we identified plasma protein signatures associated with future ILD risk among more than 52,000 participants with long-term follow-up. A total of 277 proteins were independently associated with incident ILD, and five proteins (GDF15, LAMP3, SFTPD, EDA2R, and WFDC2) emerged as dominant predictors. When combined with clinical variables, these proteins achieved good discrimination for 5-year and 10-year ILD risk. Notably, several candidate proteins showed measurable changes more than a decade before diagnosis, suggesting that plasma proteomics may capture early molecular alterations during the preclinical phase of ILD.

Our findings overlap with and extend previous proteomic studies on ILD. GDF15, LAMP3, and WFDC2 have been associated with incident IPF (29), whereas SFTPD and CXCL13 have been linked to progression or adverse outcomes in established ILD (30). Other studies have highlighted markers such as LTBP2, KRT19, ITGB6, SPINT1, and MMP7 (30, 31). These differences may reflect variations in study populations, ILD subtypes, disease stage, clinical endpoints, proteomic platforms, and analytical strategies (30–32). In contrast to studies focused primarily on IPF or established ILD, our study characterized a broader prediagnostic proteomic landscape of ILD in the general population, including CTD-ILD and non-CTD-ILD, temporal protein trajectories before diagnosis, long-term risk prediction, and integration with single-cell lung transcriptomic data to explore potential cellular origins. These complementary analyses provide additional temporal and biological context for circulating proteomic alterations associated with incident ILD.

The five leading biomarkers may reflect complementary aspects of ILD biology rather than a single shared pathogenic pathway. GDF15 is a stress-responsive protein expressed by lung epithelial cells, with circulating levels associated with IPF severity and poorer outcomes, supporting its relevance to epithelial stress (33). LAMP3 localizes to lamellar bodies in alveolar type II cells, and experimental deficiency disrupts surfactant homeostasis, linking it to alveolar epithelial function (34). SFTPD encodes surfactant protein D, which participates in pulmonary innate immunity and alveolar homeostasis and has been investigated as a circulating biomarker in ILD (30). EDA2R is associated with aging across tissues and can mediate inflammatory responses in experimental systems, although its specific role in ILD remains uncertain (35). WFDC2 encodes HE4, which has been experimentally implicated in pulmonary fibrosis through annexin II–mediated epithelial–fibroblast communication and fibroblast activation (36). Together, these observations provide a biological context for the prediagnostic proteomic signature, potentially linking epithelial stress, altered pulmonary homeostasis, inflammation, and tissue remodeling. However, prediagnostic associations may reflect subclinical disease rather than causal drivers and do not establish a shared pathogenic mechanism. Further experimental validation is required.

Consistent with their prospective associations, GDF15, LAMP3, SFTPD, EDA2R, and WFDC2 were also positively associated with prevalent ILD among UK Biobank participants with baseline ILD and available proteomic data. Such consistency is biologically plausible if these proteins reflect processes shared between preclinical and established ILD. However, this within-cohort analysis does not constitute independent or prospective validation, and replication in independent, clinically, and radiologically adjudicated ILD cohorts remains important. Temporal trajectory analysis provides important biological context. ILD-associated proteins did not follow a single pattern but were separated into four clusters with distinct temporal dynamics (37). Some proteins showed early and sustained elevation, whereas others increased progressively as the diagnosis approached or displayed more modest average changes with divergent individual patterns. The enrichment of these clusters in immune activation, cytokine signaling, leukocyte adhesion, extracellular matrix organization, epithelial or growth-factor signaling, and inflammatory responses suggests that preclinical ILD may involve coordinated but temporally distinct biological processes. These findings support the concept that ILD development is not limited to a late fibrotic event but may be preceded by long-standing systemic molecular changes involving epithelial injury, immune dysregulation, and matrix remodeling (38).

Our study also identified a distinct plasma proteomic signature in CTD-ILD, characterized by the upregulation of CXCL13, CCL7, and PDCD1. The CXCL13-CXCR5 axis plays a pivotal role in the immune microenvironment by primarily recruiting lymphocytes, such as B cells and specific T cell subsets. This recruitment is crucial for the formation of tertiary lymphoid structures, which is consistent with its role in coordinating adaptive immune responses and may be associated with a more favorable prognosis in certain diseases (39). CCL7 facilitates the recruitment of innate immune cells, such as monocytes and neutrophils, to sites of inflammation through its interactions with multiple receptors (CCR1, CCR2, and CCR3). This mechanism supports its involvement in the early inflammatory phase of CTD-ILD (39). Elevated PDCD1 expression is frequently indicative of T-cell exhaustion and dysfunction, suggesting a mechanism by which persistent autoimmune activity may be sustained in CTD-ILD. This study underscores that the proteomic profile of CTD-ILD is distinctly inflammatory, rooted in rheumatic immunity, and driven by adaptive immune dysregulation (39). While highlighting novel targets, the translational promise of these biomarkers depends on future validation in CTD-ILD-specific studies.

Reliable biomarkers and predictive models for ILD remain a central challenge, as existing studies frequently demonstrate limitations in terms of accuracy, generalizability, and long-term prognostic value. For instance, Jehn et al. (40) identified serum biomarkers, such as KL-6, as indicators of ILD progression; however, the modest AUC value of 0.61 constrains their utility for high-confidence, early risk stratification in clinical practice. Similarly, cross-sectional studies, such as that by Wheeler et al. (41) in patients with RA, have explored multi-component biomarker panels related to immune responses and tissue remodeling in patients with RA. Although these models achieved AUCs between 0.739 and 0.751, their cross-sectional design inherently limits their ability to predict future disease progression, which is critical for early interventions. Furthermore, while promising plasma proteomic biomarkers developed by Rose et al. (42) achieved a more robust AUC of 0.88, their validation in larger cohorts is essential to ensure broader applicability beyond the specific population from which they were derived.

Our study demonstrated that integrating plasma protein biomarkers with clinical variables markedly improved ILD prediction. Our combined model achieved AUCs of 0.938 and 0.908 for 5-year- and 10-year ILD prediction, respectively, which were higher than those reported in prior models based solely on clinical, genomic, or imaging features (43). Notably, the capacity to predict ILD up to a decade before its clinical onset suggests that plasma proteins may capture preclinical molecular perturbations that are not reflected in conventional clinical parameters. These findings underscore the utility of plasma proteins as promising biomarkers for the early detection of ILD and risk stratification in asymptomatic patients. Developing a reliable, reproducible, and minimally invasive blood-based test with high predictive accuracy may facilitate the early identification of individuals who warrant further diagnostic evaluation, such as high-resolution chest computed tomography (CT) scans. This strategy may reduce screening barriers, minimize radiation exposure, lower healthcare costs, and ultimately improve timely diagnosis and disease management.

Single-cell transcriptomic analysis revealed that the five plasma biomarkers predominantly originated from dysregulated epithelial compartments in patients with IPF. GDF15, LAMP3, and SFTPD were enriched in alveolar type 2 cells, whereas EDA2R and WFDC2 were associated with aberrant basal and secretory lineages, respectively. Recent studies have established epithelial senescence, AT2 cell dysfunction, and airway basal cell reprogramming as early central drivers of fibrogenesis (44–46). Our findings are consistent with these observations, suggesting that systemic proteomic alterations mirror local epithelial remodeling and stress responses. By integrating plasma proteomics with single-cell resolution, our study may help bridge population-level biomarker discovery with tissue-level pathology, underscoring epithelial dysregulation as both the source and hallmark of early ILD and highlighting it as a promising target for preventive and disease-modifying strategies in ILD.

Although our study provides insights into ILD risk prediction, it is essential to acknowledge its limitations to ensure an appropriate interpretation of the findings. First, ILD was ascertained from healthcare records rather than centralized imaging or pathological adjudication. The lack of systematic high-resolution computed tomography (HRCT) and histopathological data precluded reliable classification of usual interstitial pneumonia (UIP) versus non-UIP patterns and limited subtype-specific interpretation of our findings. Although self-reported cases were excluded and the main associations remained robust under a stricter hospital-based definition, some diagnostic misclassification remains possible. In addition, the predominantly European ancestry of UK Biobank participants, the higher ILD incidence in the proteomic subset, non-random selection of some UKB-PPP participants, and healthy volunteer bias may limit generalizability. Second, despite the implementation of rigorous quality control procedures, the possibility of residual confounding from unmeasured or unknown factors cannot be eliminated in this study. Third, proteomic profiling was performed only at baseline; therefore, the prediagnostic trajectories reflect population-level patterns rather than true within-individual longitudinal changes. Although these proteins were measured before the first recorded ILD diagnosis, this does not establish specificity for future disease risk, as subclinical or unrecognized ILD may already have been present at baseline. Their similar alterations in prevalent ILD further suggest that they may reflect pathobiological processes shared across preclinical and established diseases. Therefore, we interpret these as prediagnostic proteins associated with incident ILD and with potential predictive utility, rather than biomarkers specific to an early disease stage. Fourth, the predictive model was assessed primarily by internal validation; thus, neither the model nor the individual proteins should be considered as clinically validated predictive biomarkers. Independent prospective cohorts with adjudicated ILD, serial proteomics, and longitudinal imaging are needed to establish temporal specificity, generalizability, and clinical predictive value. Fifth, the single-cell transcriptomic analysis was based on an IPF dataset; therefore, the observed cellular expression patterns may not fully represent other ILD subtypes with distinct cellular and molecular architectures. These findings should be considered exploratory and require validation in single-cell datasets encompassing a broader spectrum of ILDs.

Future studies should prioritize addressing these limitations through three key approaches: implementing longitudinal designs with serial proteomic measurements to capture the temporal dynamics of protein expression throughout ILD development, integrating objective clinical measures to complement and validate diagnostic data, and establishing large-scale, multicenter collaborations to aggregate datasets from diverse cohorts, thereby enhancing statistical power and improving the generalizability of prediction models.

In summary, our findings show that plasma proteomics is a useful tool for predicting the risk of future ILD. The identified biomarkers and models provide a foundation for advancing early detection strategies and underscore the need for future research to validate their utility in guiding preventive interventions for ILD.

## Data Availability

The original contributions presented in the study are included in the article/**Supplementary Material**. Further inquiries can be directed to the corresponding authors.
