# Supplementary material for: Large-scale plasma proteomics identifies proteins and pathways associated with incident interstitial lung disease
Source: Front Immunol. 2026 Oct 21;17:1962219. doi: 10.3389/fimmu.2026.1962219 (PMC13731268; doi:10.3389/fimmu.2026.1962219)
Supplement: Supplementary file 1 [file DataSheet1.pdf]

Supplementary Materials

Large-scale plasma proteomics identifies proteins and pathways associated with incident interstitial lung disease

Zijian Kang, MD[1#], Ying Zhu, MD, PhD [2#], Jianzheng Zhang, MD, PhD[3#], Chen Zhu, PhD[4], Ping Li, PhD[5], Kai Li, PhD[5], Qiang Tong, MD, PhD[6*] and Sheng-Ming Dai, MD, PhD[1*]

1. Department of Rheumatology and Immunology, Shanghai Sixth People's Hospital Affiliated to Shanghai Jiao Tong University School of Medicine, Shanghai, China
2. Senior Department of Respiratory and Critical Care Medicine, The Eighth Medical Center of Chinese PLA General Hospital, Beijing, China
3. Senior Department of Orthopedics, The Fourth Medical Center of Chinese PLA General Hospital, Beijing, China
4. College of Economics and Management, China Agricultural University, Beijing, China
5. College of Biological Science and Medical Engineering, Donghua University, Shanghai, China
6. Department of Rheumatology and Immunology, Beijing Jishuitan Hospital, Capital Medical University, Beijing, China

These authors contributed equally to this work.

*Corresponding author:

Prof. Sheng-Ming Dai, Department of Rheumatology and Immunology, Shanghai Sixth People's Hospital Affiliated to Shanghai Jiao Tong University School of Medicine, Shanghai, China (shengmingdai@163.com)

Dr. Qiang Tong, Department of Rheumatology and Immunology, Beijing Jishuitan Hospital, Capital Medical University, Beijing, China (jasontong1985@outlook.com)

Appendices index

Supplementary Table 1. Extraction of data variables from the UK Biobank ... 3
Supplementary Table 2. ICD code for disease definition ... 7
Supplementary Table 3. Baseline characteristics of participants with and without available plasma proteomic data ... 8
Supplementary Table 4. Incidence of ILD among participants with and without available plasma proteomic data ... 10
Supplementary Table 5. Baseline characteristics of the UK Biobank proteomic cohort according to incident ILD status during follow-up ... 11
Supplementary Table 6. Proteomics-based Cox regression model for incident ILD ... 13
Supplementary Table 7. The GO, KEGG, and Reactome enrichment analyses of proteins related to ILD ... 27
Supplementary Table 8. Baseline characteristics and covariate balance after three-group propensity-score matching ... 38
Supplementary Table 9. Differential plasma protein expression between CTD-ILD and non-CTD-ILD ... 40
Supplementary Table 10. Model evaluation for incident ILD ... 44
Supplementary Figure 1. Evidence sources for incident composite ILD ascertainment ... 45
Supplementary Figure 2. Volcano plots of protein–ILD associations in the sensitivity analysis ... 46
Supplementary Figure 3. Sensitivity of protein-ILD associations to additional clinical adjustment ... 47
Supplementary Figure 4. STRING functional network and module enrichment of ILD-associated proteins ... 48
Supplementary Figure 5. LASSO (L1 regularization) protein screening ... 49
Supplementary Figure 6. Protein model explanation by the SHAP method in ILD ... 50
Supplementary Figure 7. Kaplan–Meier curves showing the cumulative incidence of ILD according to protein levels ... 51
Supplementary Figure 8. Predictive performance of baseline protein levels on the risk of ILD ... 52
Supplementary Figure 9. Cross-sectional assessment of five candidate proteins in prevalent ILD ... 53
Supplementary Figure 10. Baseline protein levels across ILD groups and prospective associations in landmark analyses ... 54
Supplementary Figure 11. Functional enrichment analysis of trajectory-based protein clusters ... 55
Supplementary Figure 12. LASSO (L1 regularization) clinical feature screening ... 56
Supplementary Figure 13. Clinical model explanation by the SHAP method in ILD ... 57
Supplementary Figure 14. Marker gene expression in the single-cell RNA sequencing data from idiopathic pulmonary fibrosis (IPF) lungs. ... 58

Supplementary Table 1. Extraction of data variables from the UK Biobank

Category	Field ID	Description
Baseline characteristics	21022	Age at recruitment
	33	Date of birth
	31	Sex
	21000	Ethnic background
	54	UK Biobank assessment centre
	53	Date of attending assessment centre
	21003	Age when attended assessment centre
Body composition	21001	Body mass index
	23128	Trunk fat mass
	48	Waist circumference
	49	Hip circumference
	23105	Basal metabolic rate
	50	Standing height
	21002	Weight
Disease definition	131528	Date J84 first reported
	41270	Diagnoses - ICD10
	41280	Date of first in-patient diagnosis - ICD10
	41202	Diagnoses - main ICD10
	41203	Diagnoses - main ICD9
	41204	Diagnoses - secondary ICD10
	41205	Diagnoses - secondary ICD9
Employment	709	Number in household
	738	Average total household income before tax
	22189	Townsend deprivation index at recruitment
	6138	Qualifications
	6142	Current employment status
	20277	Job code at visit
	3426	Job involves night shift work
	806	Job involves mainly walking or standing
	816	Job involves heavy manual or physical work
Smoking and drinking	20116	Smoking status
	20161	Pack years of smoking
	1239	Current tobacco smoking
	1249	Past tobacco smoking
	2644	Light smokers, at least 100 smokes in lifetime
	20117	Alcohol drinker status
	20403	Amount of alcohol drunk on a typical drinking day
	1558	Alcohol intake frequency
	100580	Alcohol consumed

Sleep and activity	1160	Sleep duration
	22040	Summed MET minutes per week for all activity
	22032	IPAQ activity group
	22033	Summed days activity
	22034	Summed minutes activity
	22037	MET minutes per week for walking
	22038	MET minutes per week for moderate activity
	22039	MET minutes per week for vigorous activity
Blood pressure	93	Systolic blood pressure, manual reading
	94	Diastolic blood pressure, manual reading
	4080	Systolic blood pressure, automated reading
	4079	Diastolic blood pressure, automated reading
Lost to follow-up and death	191	Date lost to follow-up
	190	Reason lost to follow-up
	40000	Date of death
	40001	Underlying (primary) cause of death: ICD10
	40002	Contributory (secondary) causes of death: ICD10
Spirometry	3062	Forced vital capacity (FVC)
	20257	Forced vital capacity (FVC) Z-score
	3063	Forced expiratory volume in 1-second (FEV1)
	20153	Forced expiratory volume in 1-second (FEV1), predicted
	20154	Forced expiratory volume in 1-second (FEV1), predicted percentage
	20256	Forced expiratory volume in 1-second (FEV1) Z-score
	20258	FEV1/ FVC ratio Z-score
Comorbidity	2443	Diabetes diagnosed by doctor
	20544	Mental health problems ever diagnosed by a professional
	3627	Age angina diagnosed
	4056	Age stroke diagnosed
	3992	Age emphysema/chronic bronchitis diagnosed
	29976	Age diabetes diagnosed
Medications	6177	Medication for cholesterol, blood pressure or diabetes
	6153	Medication for cholesterol, blood pressure, diabetes, or take exogenous hormones
	20003	Treatment/medication code
Depression	20507	Recent feelings of inadequacy
	20508	Recent trouble concentrating on things
	20510	Recent feelings of depression
	20511	Recent poor appetite or overeating

	20513	Recent thoughts of suicide or self-harm
	20514	Recent lack of interest or pleasure in doing things
	20517	Trouble falling or staying asleep, or sleeping too much
	20518	Recent changes in speed/amount of moving or speaking
	20519	Recent feelings of tiredness or low energy
	20448	Professional informed about depression
Anxiety	20505	Recent easy annoyance or irritability
	20506	Recent feelings or nervousness or anxiety
	20509	Recent inability to stop or control worrying
	20512	Recent feelings of foreboding
	20515	Recent trouble relaxing
	20516	Recent restlessness
	20520	Recent worrying too much about different things
	20428	Professional informed about anxiety
Pain questionnaire	6159	Pain type(s) experienced in last month
	3571	Back pain for 3+ months
	2956	General pain for 3+ months
	3799	Headaches for 3+ months
	3414	Hip pain for 3+ months
	3773	Knee pain for 3+ months
	3404	Neck/shoulder pain for 3+ months
Experience of pain	120017	Ever had pelvic pain
	120020	Length of time of pain or discomfort
	120021	Pain or discomfort all over the body in the last 3 months
	120022	All over body pain in last three months and rating of pain
	120025	Neck or shoulder pain in last three months and rating of pain
	120026	Back pain in last three months and rating of pain
	120028	Hip pain in last three months and rating of pain
	120029	Knee pain in last three months and rating of pain
	120030	Arm pain in last three months and rating of pain
	120035	Chest pain in last three months and rating of pain
	120040	Fatigue severity over the past week
Blood biochemistry	30620	Alanine aminotransferase
	30600	Albumin
	30610	Alkaline phosphatase
	30630	Apolipoprotein A
	30640	Apolipoprotein B
	30650	Aspartate aminotransferase

	30710	C-reactive protein
	30680	Calcium
	30690	Cholesterol
	30700	Creatinine
	30720	Cystatin C
	30660	Direct bilirubin
	30730	Gamma glutamyltransferase
	30740	Glucose
	30750	Glycated haemoglobin (HbA1c)
	30760	HDL cholesterol
	30770	IGF-1
	30780	LDL direct
	30790	Lipoprotein A
	30800	Oestradiol
	30810	Phosphate
	30820	Rheumatoid factor
	30830	SHBG
	30850	Testosterone
	30840	Total bilirubin
	30860	Total protein
	30870	Triglycerides
	30880	Urate
	30670	Urea
	30890	Vitamin D
Proteomics	30903	UKB-PPP Consortium selected participant
	30900	Number of proteins measured
	30901	Plate used for sample run
	30902	Well used for sample run
Ari pollution	24016	Nitrogen dioxide air pollution; 2005
	24017	Nitrogen dioxide air pollution; 2006
	24018	Nitrogen dioxide air pollution; 2007
	24003	Nitrogen dioxide air pollution; 2010
	24004	Nitrogen oxides air pollution; 2010
	24019	Particulate matter air pollution (pm10); 2007
	24005	Particulate matter air pollution (pm10); 2010
	24007	Particulate matter air pollution (pm2.5) absorbance; 2010
	24006	Particulate matter air pollution (pm2.5); 2010
	24008	Particulate matter air pollution 2.5-10um; 2010

Supplementary Table 2. ICD code for disease definition

**Disease**	**ICD-10 codes**
Other Interstitial lung disease	J84.0, J84.1, J84.8, J84.9
Pulmonary sarcoidosis	D86.0, D86.2
Hypersensitivity pneumonitis	J67.0–J67.9
Pulmonary eosinophilia	J82
Rheumatoid arthritis	M05, M06
Systemic lupus erythematosus	M32
Dermatopolymyositis	M33
Sjögren's syndrome	M35
Systemic sclerosis	M34

Supplementary Table 3. Baseline characteristics of participants with and without available plasma proteomic data

Characteristic	Overall	No proteomics	Proteomics	P value	SMD
	501317	448574	52743		
**Age, years (median [IQR])**	58.00 [50.00, 63.00]	58.00 [50.00, 63.00]	58.00 [50.00, 64.00]	<0.001	0.036
**Sex (%)**				0.026	0.01
Female	272840 (54.4)	244376 (54.5)	28464 (54.0)		
Male	228477 (45.6)	204198 (45.5)	24279 (46.0)		
**Ethnicity (%)**				<0.001	0.043
White	471035 (94.0)	421918 (94.1)	49117 (93.1)		
Other	26824 (5.4)	23533 (5.2)	3291 (6.2)		
**Townsend deprivation index (%)**					
1 (least deprived)	125425 (25.0)	112425 (25.1)	13000 (24.6)	<0.001	0.039
2	125062 (24.9)	112272 (25.0)	12790 (24.2)		
3	125385 (25.0)	112418 (25.1)	12967 (24.6)		
4 (most deprived)	124822 (24.9)	110902 (24.7)	13920 (26.4)		
**BMI category (%)**				<0.001	0.023
Underweight	2616 (0.5)	2349 (0.5)	267 (0.5)		
Normal	162077 (32.3)	145285 (32.4)	16792 (31.8)		
Overweight	211663 (42.2)	189120 (42.2)	22543 (42.7)		
Obese	121870 (24.3)	108987 (24.3)	12883 (24.4)		
**Smoking status (%)**				0.002	0.018
Never	273036 (54.5)	244471 (54.5)	28565 (54.2)		
Past	172525 (34.4)	154170 (34.4)	18355 (34.8)		
Current	52813 (10.5)	47245 (10.5)	5568 (10.6)		
**Alcohol intake frequency (%)**				<0.001	0.027
Never	40467 (8.1)	35921 (8.0)	4546 (8.6)		
Occasional	242566 (48.4)	216978 (48.4)	25588 (48.5)		
Frequent	216788 (43.2)	194304 (43.3)	22484 (42.6)		
**Physical activity (%)**				0.022	0.014
Low	70749 (14.1)	63083 (14.1)	7666 (14.5)		
Medium	194797 (38.9)	174486 (38.9)	20311 (38.5)		
High	118902 (23.7)	106451 (23.7)	12451 (23.6)		
**Hypertension (%)**	265091 (52.9)	236769 (52.8)	28322 (53.7)	<0.001	0.018
**Diabetes (%)**	20474 (4.1)	18181 (4.1)	2293 (4.3)	0.001	0.015
**Cardiovascular disease (%)**	23284 (4.6)	20535 (4.6)	2749 (5.2)	<0.001	0.029
**Air pollution score (median [IQR])**	63.43 [58.84, 68.49]	63.42 [58.83, 68.47]	63.58 [58.93, 68.72]	<0.001	0.025
**FEV1, % predicted (median [IQR])**	93.65 [83.28, 103.52]	93.66 [83.35, 103.53]	93.54 [82.75, 103.44]	0.005	0.020

**FVC, % predicted (median [IQR])**	97.98 [88.31, 107.67]	98.00 [88.35, 107.67]	97.78 [87.94, 107.68]	0.025	0.015
**FEV1/FVC ratio (median [IQR])**	0.76 [0.72, 0.80]	0.76 [0.72, 0.80]	0.76 [0.72, 0.80]	<0.001	0.028
**GERD before baseline (%)**	30231 (6.0)	26957 (6.0)	3274 (6.2)	0.072	0.008
**Substance-related disorder before baseline (%)**	4401 (0.9)	3886 (0.9)	515 (1.0)	0.011	0.012
**Systemic corticosteroid use at baseline (%)**	3401 (0.7)	2891 (0.6)	510 (1.0)	<0.001	0.048
**ILD-associated medication use at baseline (%)**	4752 (0.9)	4110 (0.9)	642 (1.2)	<0.001	0.043

Supplementary Table 4. Incidence of ILD among participants with and without available plasma proteomic data

Group	Participants	Events	Cumulative incidence percent	Person-years	Incidence per 1000 person-years	Rate 95% CI lower	Rate 95% CI upper
No proteomics	448574	4849	1.081	6291148	0.771	0.749	0.793
Proteomics	52743	814	1.543	731891.4	1.112	1.037	1.191

Supplementary Table 5. Baseline characteristics of the UK Biobank proteomic cohort according to incident ILD status during follow-up

**Characteristics**	**Overall**	**No incident ILD**	**Incident ILD**	**P value**	**SMD**
**Participants, n**	52743	51929	814		
**Age, years, median [IQR]**	58.00 [50.00, 64.00]	58.00 [50.00, 63.00]	64.00 [59.25, 67.00]	<0.001	0.698
**Sex, n (%)**				<0.001	0.199
Female	28464 (54.0)	28104 (54.1)	360 (44.2)		
Male	24279 (46.0)	23825 (45.9)	454 (55.8)		
**Ethnicity, n (%)**				0.362	0.036
White	49117 (93.1)	48353 (93.1)	764 (93.9)		
Other	3291 (6.2)	3247 (6.3)	44 (5.4)		
**Townsend deprivation quartile, n (%)**				<0.001	0.203
1 (least deprived)	13000 (24.6)	12838 (24.7)	162 (19.9)		
2	12790 (24.2)	12618 (24.3)	172 (21.1)		
3	12967 (24.6)	12774 (24.6)	193 (23.7)		
4 (most deprived)	13920 (26.4)	13634 (26.3)	286 (35.1)		
**BMI category, n (%)**				<0.001	0.251
Underweight	267 (0.5)	260 (0.5)	7 (0.9)		
Normal	16792 (31.8)	16610 (32.0)	182 (22.4)		
Overweight	22543 (42.7)	22195 (42.7)	348 (42.8)		
Obese	12883 (24.4)	12616 (24.3)	267 (32.8)		
**Smoking status, n (%)**				<0.001	0.368
Never	28565 (54.2)	28270 (54.4)	295 (36.2)		
Past	18355 (34.8)	17961 (34.6)	394 (48.4)		
Current	5568 (10.6)	5453 (10.5)	115 (14.1)		
**Alcohol intake frequency, n (%)**				0.005	0.107
Never	4546 (8.6)	4451 (8.6)	95 (11.7)		
Occasional	25588 (48.5)	25200 (48.5)	388 (47.7)		
Frequent	22484 (42.6)	22159 (42.7)	325 (39.9)		
**Physical activity (MET category), n (%)**				<0.001	0.182
Low	7666 (14.5)	7513 (14.5)	153 (18.8)		
Medium	20311 (38.5)	20044 (38.6)	267 (32.8)		
High	12451 (23.6)	12292 (23.7)	159 (19.5)		
**Hypertension, n (%)**	28322 (53.7)	27772 (53.5)	550 (67.6)	<0.001	0.291
**Diabetes, n (%)**	2293 (4.3)	2205 (4.2)	88 (10.8)	<0.001	0.251
**Cardiovascular disease, n (%)**	2749 (5.2)	2617 (5.0)	132 (16.2)	<0.001	0.371

**Air pollution score, median [IQR]**	63.58 [58.93, 68.72]	63.56 [58.91, 68.71]	64.47 [60.10, 69.29]	<0.001	0.144
**FEV1, % predicted, median [IQR]**	93.54 [82.75, 103.44]	93.63 [82.90, 103.53]	84.19 [73.31, 95.63]	<0.001	0.511
**FVC, % predicted, median [IQR]**	97.78 [87.94, 107.68]	97.89 [88.06, 107.76]	89.38 [78.55, 99.01]	<0.001	0.539
**FEV1/FVC, median [IQR]**	0.76 [0.72, 0.80]	0.76 [0.72, 0.80]	0.75 [0.71, 0.79]	<0.001	0.174
**GERD before baseline, n (%)**	3279 (6.2)	3179 (6.1)	100 (12.3)	<0.001	0.214
**Substance-related disorder, n (%)**	515 (1.0)	496 (1.0)	19 (2.3)	<0.001	0.109
**Systemic corticosteroid use at baseline, n (%)**	510 (1.0)	460 (0.9)	50 (6.1)	<0.001	0.288
**ILD-associated medication use, n (%)**	642 (1.2)	598 (1.2)	44 (5.4)	<0.001	0.241

Supplementary Table 6. Proteomics-based Cox regression model for incident ILD

**Proteins**	**Model 1**			**Model 2**		
	HR	P value	Bonferroni	HR	P value	Bonferroni
ACVRL1	3.189 (2.545, 3.997)	7.73E-24	2.26E-20	2.82 (2.093, 3.801)	9.85E-12	2.88E-08
ADA2	2.74 (2.392, 3.139)	5.96E-48	1.74E-44	3.067 (2.598, 3.62)	4.29E-40	1.25E-36
ADAM8	2.712 (2.233, 3.294)	8.06E-24	2.36E-20	2.376 (1.826, 3.092)	1.17E-10	3.42E-07
ADGRE5	1.866 (1.522, 2.288)	1.96E-09	5.73E-06	2.327 (1.798, 3.013)	1.46E-10	4.26E-07
ADGRG1	1.373 (1.28, 1.472)	6.74E-19	1.97E-15	1.369 (1.25, 1.498)	1.05E-11	3.05E-08
ADM	3.238 (2.481, 4.227)	5.49E-18	1.60E-14	2.754 (1.966, 3.86)	3.97E-09	1.16E-05
AGRN	2.581 (2.23, 2.988)	5.99E-37	1.75E-33	2.812 (2.307, 3.427)	1.31E-24	3.82E-21
AHNAK	2.194 (1.805, 2.666)	3.01E-15	8.80E-12	2.315 (1.852, 2.894)	1.66E-13	4.86E-10
ALPP	1.359 (1.293, 1.429)	3.74E-33	1.09E-29	1.357 (1.261, 1.461)	4.47E-16	1.31E-12
ANGPT2	2.053 (1.736, 2.427)	4.37E-17	1.28E-13	1.913 (1.544, 2.37)	3.09E-09	9.05E-06
AOC3	2.568 (2.035, 3.24)	1.95E-15	5.69E-12	2.682 (2.003, 3.591)	3.56E-11	1.04E-07
AREG	3.041 (2.748, 3.366)	1.03E-102	3.02E-99	3.034 (2.673, 3.444)	4.20E-66	1.23E-62
ATRAID	1.648 (1.436, 1.891)	1.15E-12	3.36E-09	1.622 (1.38, 1.905)	4.12E-09	1.20E-05
B2M	1.743 (1.573, 1.932)	3.38E-26	9.89E-23	1.698 (1.506, 1.915)	6.25E-18	1.83E-14
B4GALT1	2.373 (1.863, 3.024)	2.68E-12	7.84E-09	2.654 (1.973, 3.57)	1.12E-10	3.27E-07
BAP18	1.364 (1.252, 1.485)	1.19E-12	3.49E-09	1.396 (1.26, 1.547)	2.06E-10	6.02E-07
BCAM	2.546 (1.989, 3.259)	1.20E-13	3.51E-10	2.925 (2.158, 3.964)	4.51E-12	1.32E-08
BPIFB1	2.141 (1.917, 2.392)	2.23E-41	6.51E-38	2.223 (1.934, 2.555)	2.44E-29	7.13E-26
BSG	5.456 (4.062, 7.329)	1.78E-29	5.22E-26	5.866 (4.091, 8.412)	6.56E-22	1.92E-18

BST2	1.509 (1.355, 1.681)	7.48E-14	2.19E-10	1.477 (1.295, 1.683)	5.53E-09	1.62E-05
BTN2A1	2.836 (2.265, 3.55)	9.51E-20	2.78E-16	3.188 (2.409, 4.219)	5.15E-16	1.51E-12
C9	1.812 (1.544, 2.127)	3.35E-13	9.79E-10	1.841 (1.513, 2.24)	1.10E-09	3.22E-06
CAPG	1.859 (1.681, 2.056)	1.24E-33	3.63E-30	1.844 (1.627, 2.09)	8.41E-22	2.46E-18
CCL17	1.567 (1.455, 1.686)	7.12E-33	2.08E-29	1.6 (1.461, 1.753)	6.23E-24	1.82E-20
CCL18	1.53 (1.435, 1.63)	3.23E-39	9.43E-36	1.475 (1.363, 1.597)	4.53E-22	1.32E-18
CCL20	1.295 (1.217, 1.377)	2.21E-16	6.45E-13	1.286 (1.188, 1.392)	5.36E-10	1.57E-06
CCL21	1.764 (1.592, 1.954)	1.86E-27	5.44E-24	1.664 (1.44, 1.924)	5.48E-12	1.60E-08
CCL22	1.762 (1.577, 1.969)	1.45E-23	4.23E-20	1.645 (1.426, 1.897)	7.85E-12	2.29E-08
CCL3	1.352 (1.261, 1.451)	2.96E-17	8.66E-14	1.316 (1.204, 1.439)	1.67E-09	4.89E-06
CCL7	1.757 (1.638, 1.885)	8.87E-56	2.59E-52	1.794 (1.639, 1.963)	5.16E-37	1.51E-33
CCN3	1.872 (1.555, 2.254)	3.65E-11	1.07E-07	2.005 (1.596, 2.519)	2.34E-09	6.85E-06
CD163	1.957 (1.683, 2.275)	2.39E-18	6.98E-15	2.093 (1.736, 2.524)	9.54E-15	2.79E-11
CD22	1.764 (1.525, 2.04)	2.20E-14	6.42E-11	1.734 (1.445, 2.082)	3.34E-09	9.76E-06
CD27	2.796 (2.463, 3.174)	8.56E-57	2.50E-53	2.893 (2.465, 3.394)	9.16E-39	2.68E-35
CD274	2.416 (2.086, 2.798)	5.37E-32	1.57E-28	2.502 (2.055, 3.045)	5.94E-20	1.74E-16
CD300A	2.673 (2.082, 3.432)	1.22E-14	3.58E-11	2.564 (1.879, 3.501)	3.00E-09	8.76E-06
CD300C	2.629 (2.187, 3.161)	8.51E-25	2.49E-21	2.607 (2.062, 3.296)	1.13E-15	3.31E-12
CD300E	2.195 (1.893, 2.545)	2.22E-25	6.49E-22	2.119 (1.753, 2.56)	7.53E-15	2.20E-11
CD46	2.365 (1.946, 2.873)	4.52E-18	1.32E-14	2.527 (2.005, 3.185)	4.30E-15	1.26E-11
CD5	1.864 (1.615, 2.152)	1.59E-17	4.65E-14	1.738 (1.449, 2.083)	2.45E-09	7.16E-06

CD70	1.711 (1.464, 1.999)	1.37E-11	3.99E-08	1.806 (1.494, 2.184)	1.02E-09	2.99E-06
CD72	2.036 (1.733, 2.393)	6.08E-18	1.78E-14	2.086 (1.701, 2.559)	1.74E-12	5.09E-09
CD74	3.136 (2.646, 3.717)	1.24E-39	3.63E-36	3.008 (2.43, 3.724)	4.71E-24	1.38E-20
CD79B	2.059 (1.827, 2.322)	3.47E-32	1.01E-28	2.035 (1.755, 2.361)	5.58E-21	1.63E-17
CD80	4.894 (4.251, 5.634)	3.44E-108	1.01E-104	5.234 (4.373, 6.265)	8.84E-73	2.58E-69
CD83	3.634 (3.122, 4.23)	2.87E-62	8.39E-59	3.652 (3.032, 4.399)	2.49E-42	7.29E-39
CD93	2.13 (1.72, 2.639)	4.40E-12	1.29E-08	2.255 (1.738, 2.925)	9.32E-10	2.72E-06
CDCP1	2.355 (2.129, 2.606)	6.92E-62	2.02E-58	2.383 (2.096, 2.709)	3.28E-40	9.59E-37
CDH3	2.307 (1.959, 2.717)	1.34E-23	3.92E-20	2.183 (1.782, 2.674)	4.86E-14	1.42E-10
CEACAM5	1.557 (1.418, 1.71)	1.45E-20	4.23E-17	1.54 (1.362, 1.741)	5.39E-12	1.58E-08
CEACAM6	2.201 (2.077, 2.332)	5.01E-156	1.46E-152	2.194 (2.03, 2.371)	1.90E-87	5.56E-84
CHCHD10	1.936 (1.678, 2.234)	1.51E-19	4.42E-16	1.968 (1.639, 2.365)	4.59E-13	1.34E-09
CHRDL1	2.853 (2.318, 3.512)	4.46E-23	1.30E-19	2.775 (2.138, 3.602)	1.70E-14	4.98E-11
CKAP4	2.645 (2.235, 3.132)	1.32E-29	3.85E-26	2.765 (2.267, 3.372)	9.71E-24	2.84E-20
CLC	1.414 (1.292, 1.548)	5.94E-14	1.74E-10	1.447 (1.299, 1.611)	1.71E-11	5.00E-08
CLEC14A	1.966 (1.601, 2.416)	1.20E-10	3.51E-07	2.201 (1.713, 2.828)	6.70E-10	1.96E-06
CLEC3B	0.192 (0.133, 0.275)	4.25E-19	1.24E-15	0.21 (0.131, 0.337)	8.60E-11	2.51E-07
CLEC5A	2.764 (2.251, 3.393)	2.73E-22	7.97E-19	2.273 (1.736, 2.977)	2.41E-09	7.05E-06
CLEC6A	2.056 (1.81, 2.335)	1.50E-28	4.39E-25	1.962 (1.668, 2.308)	4.37E-16	1.28E-12
CLEC7A	1.802 (1.547, 2.1)	4.21E-14	1.23E-10	1.794 (1.484, 2.17)	1.63E-09	4.76E-06
COL18A1	3.749 (2.815, 4.992)	1.51E-19	4.41E-16	3.331 (2.324, 4.773)	5.57E-11	1.63E-07

COL24A1	2.803 (2.394, 3.282)	1.58E-37	4.61E-34	2.645 (2.173, 3.22)	3.25E-22	9.49E-19
COL6A3	2.791 (2.389, 3.261)	2.86E-38	8.36E-35	2.801 (2.3, 3.411)	1.20E-24	3.50E-21
COLEC12	3.449 (2.759, 4.313)	1.69E-27	4.94E-24	3.333 (2.5, 4.443)	2.30E-16	6.74E-13
CRIM1	4.952 (3.645, 6.727)	1.36E-24	3.97E-21	4.55 (3.07, 6.742)	4.33E-14	1.27E-10
CSF1	3.325 (2.641, 4.186)	1.62E-24	4.75E-21	3.476 (2.601, 4.646)	3.69E-17	1.08E-13
CSF2	1.804 (1.523, 2.137)	8.06E-12	2.36E-08	1.948 (1.597, 2.377)	5.03E-11	1.47E-07
CST3	2.964 (2.42, 3.631)	9.57E-26	2.80E-22	2.941 (2.267, 3.815)	4.56E-16	1.33E-12
CSTB	2.357 (2.06, 2.697)	9.56E-36	2.80E-32	2.28 (1.931, 2.692)	2.56E-22	7.49E-19
CTSL	3.305 (2.542, 4.298)	4.49E-19	1.31E-15	2.75 (1.976, 3.826)	1.94E-09	5.68E-06
CWC15	1.39 (1.262, 1.531)	2.59E-11	7.57E-08	1.459 (1.3, 1.637)	1.46E-10	4.27E-07
CXCL10	1.56 (1.435, 1.695)	9.47E-26	2.77E-22	1.614 (1.459, 1.786)	1.76E-20	5.15E-17
CXCL11	1.363 (1.271, 1.462)	5.60E-18	1.64E-14	1.406 (1.293, 1.529)	1.71E-15	5.00E-12
CXCL13	1.572 (1.492, 1.657)	2.48E-64	7.26E-61	1.565 (1.464, 1.673)	1.75E-39	5.12E-36
CXCL16	4.102 (3.12, 5.395)	5.39E-24	1.58E-20	3.861 (2.728, 5.465)	2.49E-14	7.28E-11
CXCL17	2.324 (2.124, 2.542)	1.23E-75	3.60E-72	2.303 (2.035, 2.606)	5.30E-40	1.55E-36
CXCL9	1.608 (1.479, 1.748)	6.54E-29	1.91E-25	1.601 (1.444, 1.775)	3.71E-19	1.09E-15
DCTPP1	2.36 (1.911, 2.915)	1.51E-15	4.40E-12	2.76 (2.154, 3.537)	1.06E-15	3.09E-12
DDAH1	1.62 (1.405, 1.868)	3.38E-11	9.88E-08	1.686 (1.418, 2.004)	3.11E-09	9.09E-06
DPY30	1.561 (1.42, 1.716)	3.12E-20	9.12E-17	1.599 (1.42, 1.8)	7.90E-15	2.31E-11
DSC2	2.199 (1.859, 2.6)	3.31E-20	9.68E-17	2.18 (1.773, 2.68)	1.46E-13	4.26E-10
DTX3	2.273 (1.784, 2.896)	3.07E-11	8.98E-08	2.476 (1.833, 3.345)	3.39E-09	9.91E-06

EDA2R	3.585 (3.165, 4.061)	1.31E-89	3.82E-86	3.624 (3.095, 4.244)	1.82E-57	5.32E-54
EFEMP1	3.726 (3.078, 4.51)	1.65E-41	4.83E-38	3.548 (2.79, 4.512)	5.38E-25	1.57E-21
EFNA4	2.607 (2.208, 3.078)	1.29E-29	3.78E-26	2.629 (2.122, 3.257)	9.24E-19	2.70E-15
EGFR	0.117 (0.08, 0.169)	1.18E-29	3.44E-26	0.138 (0.086, 0.221)	1.75E-16	5.10E-13
EGLN1	1.363 (1.25, 1.486)	2.46E-12	7.19E-09	1.393 (1.255, 1.545)	3.91E-10	1.14E-06
EPHA2	2.446 (2.114, 2.83)	2.66E-33	7.78E-30	2.425 (2.026, 2.902)	4.39E-22	1.28E-18
EPS8L2	1.864 (1.518, 2.29)	2.92E-09	8.53E-06	2.186 (1.686, 2.834)	3.53E-09	1.03E-05
EZR	2.784 (2.204, 3.515)	7.90E-18	2.31E-14	2.628 (1.937, 3.567)	5.49E-10	1.60E-06
FBLN2	2.645 (2.122, 3.296)	4.57E-18	1.34E-14	2.685 (2.016, 3.575)	1.39E-11	4.07E-08
FCER2	1.512 (1.36, 1.681)	1.89E-14	5.53E-11	1.587 (1.395, 1.805)	2.29E-12	6.69E-09
FCRL5	1.711 (1.524, 1.922)	9.71E-20	2.84E-16	1.761 (1.527, 2.029)	6.06E-15	1.77E-11
FGA	2.455 (1.915, 3.148)	1.35E-12	3.93E-09	2.846 (2.096, 3.865)	2.08E-11	6.07E-08
FGF23	1.489 (1.355, 1.637)	1.29E-16	3.79E-13	1.46 (1.292, 1.65)	1.35E-09	3.96E-06
FGL1	1.981 (1.766, 2.223)	2.41E-31	7.05E-28	1.988 (1.724, 2.291)	2.60E-21	7.61E-18
FLT3LG	2.664 (2.179, 3.257)	1.24E-21	3.62E-18	3.18 (2.484, 4.071)	4.13E-20	1.21E-16
FOLR1	3.993 (3.508, 4.544)	1.23E-97	3.61E-94	5.638 (4.553, 6.982)	1.21E-56	3.55E-53
FSTL3	2.609 (2.177, 3.127)	3.08E-25	9.01E-22	2.56 (2.031, 3.228)	1.84E-15	5.39E-12
FUT3_FUT5	1.888 (1.631, 2.184)	1.39E-17	4.06E-14	1.771 (1.474, 2.128)	9.91E-10	2.90E-06
GALNT5	2.331 (1.847, 2.941)	9.72E-13	2.84E-09	2.277 (1.732, 2.994)	3.87E-09	1.13E-05
GBP1	1.478 (1.32, 1.656)	1.37E-11	4.00E-08	1.54 (1.341, 1.768)	8.96E-10	2.62E-06
GCHFR	1.903 (1.725, 2.099)	8.29E-38	2.42E-34	1.935 (1.719, 2.177)	7.76E-28	2.27E-24

GDF15	2.819 (2.626, 3.025)	8.30E-182	2.43E-178	2.947 (2.687, 3.232)	1.92E-116	5.62E-113
GM2A	1.686 (1.501, 1.893)	1.03E-18	3.00E-15	1.654 (1.434, 1.907)	5.00E-12	1.46E-08
HAVCR2	3.344 (2.854, 3.918)	1.84E-50	5.39E-47	3.39 (2.777, 4.139)	3.85E-33	1.13E-29
HGF	2.784 (2.379, 3.259)	3.38E-37	9.88E-34	2.621 (2.136, 3.216)	2.56E-20	7.47E-17
HIP1R	1.519 (1.357, 1.701)	3.95E-13	1.16E-09	1.651 (1.417, 1.924)	1.28E-10	3.73E-07
HSPG2	2.812 (2.293, 3.449)	3.14E-23	9.19E-20	2.952 (2.284, 3.816)	1.38E-16	4.05E-13
HYAL1	3.142 (2.433, 4.057)	1.71E-18	5.00E-15	3.094 (2.253, 4.25)	3.05E-12	8.91E-09
ICAM1	5.691 (4.895, 6.618)	4.40E-113	1.29E-109	5.455 (4.517, 6.588)	1.80E-69	5.27E-66
ICAM3	3.399 (2.721, 4.245)	4.04E-27	1.18E-23	3.576 (2.714, 4.711)	1.31E-19	3.84E-16
ICAM5	2.807 (2.44, 3.231)	4.62E-47	1.35E-43	2.608 (2.186, 3.113)	2.10E-26	6.13E-23
IFI30	3.294 (2.867, 3.786)	2.44E-63	7.14E-60	3.392 (2.864, 4.019)	2.52E-45	7.38E-42
IGFBP2	1.505 (1.338, 1.694)	1.10E-11	3.20E-08	1.601 (1.386, 1.85)	1.63E-10	4.78E-07
IGFBP4	1.935 (1.711, 2.19)	9.83E-26	2.87E-22	1.848 (1.572, 2.173)	1.08E-13	3.15E-10
IGFBP7	2.182 (1.876, 2.537)	4.02E-24	1.18E-20	2.131 (1.773, 2.562)	7.78E-16	2.27E-12
IGFBPL1	2.368 (1.978, 2.833)	5.23E-21	1.53E-17	2.189 (1.736, 2.761)	3.63E-11	1.06E-07
IGLC2	2.019 (1.76, 2.315)	1.03E-23	3.00E-20	2.111 (1.784, 2.496)	2.82E-18	8.25E-15
IL10RB	2.615 (2.228, 3.071)	7.74E-32	2.26E-28	2.484 (2.038, 3.029)	2.33E-19	6.82E-16
IL12RB1	1.88 (1.595, 2.216)	5.42E-14	1.58E-10	1.805 (1.482, 2.2)	4.62E-09	1.35E-05
IL15RA	2.04 (1.665, 2.5)	6.13E-12	1.79E-08	2.144 (1.667, 2.757)	2.88E-09	8.42E-06
IL18	1.619 (1.408, 1.862)	1.49E-11	4.35E-08	1.699 (1.431, 2.018)	1.41E-09	4.13E-06
IL18BP	3.249 (2.714, 3.888)	8.97E-38	2.62E-34	3.221 (2.557, 4.057)	3.05E-23	8.91E-20

IL19	1.441 (1.324, 1.569)	3.37E-17	9.84E-14	1.442 (1.301, 1.599)	3.63E-12	1.06E-08
IL1RN	1.596 (1.445, 1.762)	2.42E-20	7.08E-17	1.63 (1.443, 1.842)	4.65E-15	1.36E-11
IL22	1.381 (1.25, 1.526)	2.46E-10	7.18E-07	1.456 (1.29, 1.644)	1.22E-09	3.55E-06
IL2RA	3.015 (2.707, 3.358)	9.60E-90	2.81E-86	2.89 (2.53, 3.301)	3.68E-55	1.07E-51
IL32	1.819 (1.593, 2.077)	1.10E-18	3.21E-15	1.948 (1.667, 2.277)	5.20E-17	1.52E-13
IL6	1.399 (1.317, 1.487)	2.28E-27	6.67E-24	1.417 (1.312, 1.53)	8.73E-19	2.55E-15
ITGA5	3.451 (2.656, 4.485)	1.98E-20	5.78E-17	3.159 (2.269, 4.399)	9.75E-12	2.85E-08
ITGB6	2.871 (2.269, 3.632)	1.55E-18	4.52E-15	3.517 (2.618, 4.725)	6.70E-17	1.96E-13
KLKB1	0.297 (0.217, 0.408)	5.46E-14	1.60E-10	0.285 (0.193, 0.421)	2.79E-10	8.15E-07
KRT19	1.927 (1.776, 2.09)	2.53E-56	7.40E-53	1.915 (1.728, 2.122)	2.38E-35	6.97E-32
LAG3	1.843 (1.606, 2.115)	3.12E-18	9.11E-15	1.913 (1.616, 2.263)	4.13E-14	1.21E-10
LAIR1	2.212 (1.938, 2.524)	4.58E-32	1.34E-28	2.286 (1.933, 2.702)	3.59E-22	1.05E-18
LAMP3	4.331 (3.865, 4.852)	5.49E-141	1.61E-137	4.865 (4.174, 5.671)	4.67E-91	1.37E-87
LAYN	2.284 (1.934, 2.697)	2.13E-22	6.23E-19	2.396 (1.946, 2.951)	1.80E-16	5.26E-13
LCP1	1.637 (1.431, 1.874)	7.20E-13	2.10E-09	1.689 (1.446, 1.972)	3.39E-11	9.90E-08
LGALS1	2.319 (1.949, 2.759)	2.57E-21	7.51E-18	2.493 (2.028, 3.065)	4.27E-18	1.25E-14
LGALS9	4.656 (3.908, 5.547)	2.24E-66	6.56E-63	4.853 (3.898, 6.042)	2.59E-45	7.58E-42
LILRA5	3.841 (3.155, 4.676)	5.58E-41	1.63E-37	3.323 (2.575, 4.289)	2.78E-20	8.12E-17
LILRB1	2.748 (2.129, 3.546)	8.36E-15	2.44E-11	2.729 (1.995, 3.733)	3.45E-10	1.01E-06
LILRB4	2.317 (2.007, 2.676)	2.49E-30	7.28E-27	2.391 (1.997, 2.864)	2.69E-21	7.86E-18
LMNB1	1.424 (1.29, 1.572)	2.56E-12	7.48E-09	1.471 (1.307, 1.656)	1.61E-10	4.72E-07

LMNB2	3.563 (2.976, 4.266)	1.62E-43	4.74E-40	3.618 (2.872, 4.558)	9.39E-28	2.75E-24
LRG1	3.262 (2.497, 4.263)	4.50E-18	1.32E-14	3.312 (2.352, 4.663)	6.96E-12	2.04E-08
LRRC25	1.946 (1.734, 2.184)	1.28E-29	3.74E-26	1.858 (1.609, 2.146)	3.06E-17	8.94E-14
LTA4H	1.834 (1.661, 2.025)	2.52E-33	7.38E-30	1.811 (1.606, 2.041)	2.93E-22	8.57E-19
LTBP2	4.622 (3.887, 5.497)	3.21E-67	9.39E-64	5.023 (4.04, 6.246)	9.03E-48	2.64E-44
LTBR	2.645 (2.152, 3.252)	2.63E-20	7.68E-17	2.645 (2.037, 3.435)	3.01E-13	8.81E-10
LY96	2.308 (1.99, 2.677)	2.16E-28	6.31E-25	2.16 (1.796, 2.597)	2.74E-16	8.01E-13
MAD1L1	1.595 (1.467, 1.735)	7.12E-28	2.08E-24	1.599 (1.44, 1.775)	1.34E-18	3.93E-15
MAMDC2	2.745 (2.26, 3.334)	2.41E-24	7.04E-21	2.692 (2.11, 3.434)	1.59E-15	4.64E-12
MARCO	2.713 (2.07, 3.555)	4.74E-13	1.39E-09	2.858 (2.05, 3.985)	5.82E-10	1.70E-06
MDK	1.458 (1.313, 1.62)	1.83E-12	5.35E-09	1.492 (1.311, 1.697)	1.28E-09	3.74E-06
MMP10	1.545 (1.391, 1.717)	4.89E-16	1.43E-12	1.603 (1.403, 1.831)	3.98E-12	1.16E-08
MMP12	1.822 (1.627, 2.04)	3.04E-25	8.89E-22	1.656 (1.429, 1.919)	1.89E-11	5.51E-08
MMP7	2.764 (2.39, 3.196)	8.87E-43	2.59E-39	2.667 (2.22, 3.205)	1.25E-25	3.66E-22
MRC1	4.375 (3.479, 5.502)	1.60E-36	4.68E-33	4.242 (3.186, 5.648)	4.29E-23	1.25E-19
MSLN	2.682 (2.451, 2.934)	1.58E-102	4.63E-99	2.733 (2.425, 3.08)	4.63E-61	1.35E-57
MSR1	2.469 (2.137, 2.853)	1.42E-34	4.16E-31	2.437 (2.034, 2.92)	4.49E-22	1.31E-18
MZB1	2.456 (2.196, 2.747)	6.78E-56	1.98E-52	2.461 (2.143, 2.826)	2.84E-37	8.31E-34
NBL1	4.106 (3.281, 5.139)	5.91E-35	1.73E-31	4.115 (3.106, 5.452)	6.37E-23	1.86E-19
NCR3LG1	2.465 (2.002, 3.035)	1.80E-17	5.27E-14	2.496 (1.923, 3.24)	6.38E-12	1.86E-08
NECTIN2	3.087 (2.539, 3.754)	1.37E-29	4.02E-26	3.123 (2.453, 3.976)	2.49E-20	7.28E-17

NECTIN4	2.166 (1.825, 2.57)	8.34E-19	2.44E-15	2.283 (1.826, 2.854)	4.20E-13	1.23E-09
NELL2	2.776 (2.235, 3.447)	2.42E-20	7.06E-17	3.13 (2.419, 4.049)	3.87E-18	1.13E-14
NOS3	1.757 (1.629, 1.895)	2.39E-48	6.99E-45	1.749 (1.59, 1.925)	1.89E-30	5.52E-27
NPC2	3.123 (2.827, 3.45)	2.42E-111	7.07E-108	3.049 (2.69, 3.455)	2.34E-68	6.84E-65
NPDC1	2.389 (2.023, 2.822)	1.11E-24	3.26E-21	2.37 (1.908, 2.944)	6.16E-15	1.80E-11
NPL	1.998 (1.711, 2.334)	2.51E-18	7.34E-15	1.914 (1.57, 2.333)	1.39E-10	4.07E-07
NRCAM	2.903 (2.344, 3.596)	1.61E-22	4.72E-19	3.002 (2.272, 3.967)	1.09E-14	3.18E-11
NRP2	3.124 (2.576, 3.789)	5.44E-31	1.59E-27	3.127 (2.491, 3.926)	8.46E-23	2.47E-19
OCLN	2.77 (2.471, 3.104)	9.64E-69	2.82E-65	2.737 (2.375, 3.154)	5.83E-44	1.70E-40
OGFR	1.96 (1.602, 2.398)	6.17E-11	1.80E-07	2.172 (1.707, 2.762)	2.60E-10	7.61E-07
OSCAR	3.172 (2.581, 3.898)	5.32E-28	1.55E-24	2.92 (2.255, 3.78)	4.13E-16	1.21E-12
PDCD1	2.333 (2.092, 2.601)	2.19E-52	6.41E-49	2.389 (2.094, 2.726)	3.04E-38	8.90E-35
PGF	2.989 (2.542, 3.516)	6.04E-40	1.76E-36	3.418 (2.709, 4.313)	3.61E-25	1.05E-21
PIGR	2.633 (2.243, 3.09)	2.23E-32	6.53E-29	2.542 (2.058, 3.14)	4.61E-18	1.35E-14
PIK3IP1	2.406 (1.954, 2.961)	1.20E-16	3.52E-13	2.435 (1.873, 3.166)	3.05E-11	8.91E-08
PILRA	2.152 (1.817, 2.549)	6.57E-19	1.92E-15	2.084 (1.682, 2.582)	1.80E-11	5.27E-08
PLA2G10	1.584 (1.401, 1.79)	1.86E-13	5.43E-10	1.685 (1.447, 1.962)	1.85E-11	5.42E-08
PLA2G15	2.901 (2.284, 3.685)	2.50E-18	7.30E-15	2.678 (1.994, 3.597)	6.04E-11	1.76E-07
PLAT	1.839 (1.608, 2.102)	5.26E-19	1.54E-15	1.679 (1.419, 1.988)	1.75E-09	5.11E-06
PLAU	1.904 (1.705, 2.125)	1.84E-30	5.38E-27	1.826 (1.602, 2.081)	1.99E-19	5.82E-16
PLAUR	6.75 (5.612, 8.118)	2.17E-91	6.35E-88	6.964 (5.431, 8.929)	7.25E-53	2.12E-49

PLXNB2	2.81 (2.15, 3.674)	4.07E-14	1.19E-10	3.133 (2.203, 4.456)	2.06E-10	6.02E-07
POLR2F	1.864 (1.652, 2.104)	5.89E-24	1.72E-20	1.848 (1.593, 2.143)	4.98E-16	1.46E-12
POSTN	1.949 (1.596, 2.381)	6.01E-11	1.76E-07	2.097 (1.651, 2.664)	1.26E-09	3.68E-06
PRSS8	4.365 (3.792, 5.024)	1.51E-93	4.43E-90	4.599 (3.816, 5.542)	8.35E-58	2.44E-54
PRTN3	1.542 (1.381, 1.723)	1.69E-14	4.93E-11	1.55 (1.352, 1.777)	3.38E-10	9.87E-07
PSAP	2.393 (2.026, 2.826)	8.08E-25	2.36E-21	2.335 (1.908, 2.859)	2.10E-16	6.15E-13
PTGDS	2.53 (2.122, 3.016)	4.16E-25	1.22E-21	2.657 (2.136, 3.303)	1.55E-18	4.53E-15
PTPRN2	3.454 (2.927, 4.075)	7.55E-49	2.21E-45	3.784 (3.064, 4.672)	3.89E-35	1.14E-31
QPCT	2.599 (2.056, 3.285)	1.31E-15	3.83E-12	2.66 (1.98, 3.573)	8.05E-11	2.35E-07
RBFOX3	2.123 (1.889, 2.386)	1.08E-36	3.16E-33	2.215 (1.921, 2.554)	7.73E-28	2.26E-24
RELT	3.198 (2.656, 3.851)	1.32E-34	3.87E-31	3.352 (2.625, 4.281)	3.20E-22	9.37E-19
REN	1.383 (1.285, 1.49)	8.83E-18	2.58E-14	1.352 (1.226, 1.49)	1.55E-09	4.53E-06
RNASE1	2.474 (2.066, 2.963)	7.06E-23	2.06E-19	2.697 (2.07, 3.514)	1.95E-13	5.69E-10
RNASE6	2.066 (1.838, 2.323)	4.80E-34	1.40E-30	2.062 (1.745, 2.437)	1.87E-17	5.46E-14
RNASET2	2.813 (2.356, 3.357)	2.46E-30	7.19E-27	3.072 (2.439, 3.87)	1.58E-21	4.63E-18
ROBO1	3.032 (2.37, 3.878)	1.02E-18	2.98E-15	3.224 (2.352, 4.419)	3.36E-13	9.82E-10
ROBO2	7.251 (5.689, 9.24)	1.04E-57	3.04E-54	7.887 (5.769, 10.783)	2.59E-38	7.57E-35
RRM2	1.438 (1.315, 1.571)	1.20E-15	3.50E-12	1.506 (1.358, 1.669)	6.68E-15	1.95E-11
RSPO1	2.093 (1.771, 2.473)	4.75E-18	1.39E-14	2.017 (1.633, 2.492)	7.89E-11	2.31E-07
RSPO3	2.113 (1.692, 2.64)	4.41E-11	1.29E-07	2.28 (1.732, 3.002)	4.20E-09	1.23E-05
SCARB2	2.226 (1.867, 2.654)	4.92E-19	1.44E-15	2.345 (1.877, 2.929)	6.20E-14	1.81E-10

SCGB1A1	1.853 (1.612, 2.131)	4.49E-18	1.31E-14	2.067 (1.736, 2.46)	3.14E-16	9.17E-13
SCGB3A1	9.897 (7.5, 13.06)	4.89E-59	1.43E-55	9.852 (6.993, 13.878)	4.07E-39	1.19E-35
SCGB3A2	1.373 (1.299, 1.453)	1.28E-28	3.74E-25	1.386 (1.288, 1.491)	1.98E-18	5.80E-15
SDC1	2.21 (1.922, 2.541)	8.41E-29	2.46E-25	2.224 (1.873, 2.641)	7.92E-20	2.31E-16
SEL1L	1.7 (1.491, 1.937)	1.75E-15	5.11E-12	1.697 (1.452, 1.983)	2.98E-11	8.70E-08
SELPLG	0.427 (0.353, 0.517)	2.38E-18	6.96E-15	0.403 (0.322, 0.505)	2.91E-15	8.49E-12
SEMA7A	2.089 (1.722, 2.534)	7.33E-14	2.14E-10	2.142 (1.704, 2.693)	6.62E-11	1.93E-07
SEPTIN8	2.106 (1.759, 2.52)	4.80E-16	1.40E-12	2.085 (1.66, 2.619)	2.59E-10	7.58E-07
SERPINA4	0.324 (0.231, 0.454)	6.12E-11	1.79E-07	0.29 (0.191, 0.44)	5.68E-09	1.66E-05
SERPINB8	1.429 (1.317, 1.551)	1.06E-17	3.09E-14	1.459 (1.319, 1.613)	1.71E-13	5.00E-10
SEZ6L2	3.209 (2.565, 4.014)	1.79E-24	5.24E-21	3.538 (2.661, 4.706)	3.76E-18	1.10E-14
SFTPA1	1.428 (1.314, 1.553)	6.24E-17	1.82E-13	1.448 (1.312, 1.598)	2.03E-13	5.94E-10
SFTPA2	2.216 (2.02, 2.431)	1.44E-63	4.21E-60	2.231 (1.966, 2.533)	2.38E-35	6.97E-32
SFTPD	3.288 (2.958, 3.655)	1.01E-107	2.95E-104	3.111 (2.719, 3.558)	1.94E-61	5.67E-58
SHISA5	2.563 (2.235, 2.938)	1.98E-41	5.80E-38	2.445 (2.033, 2.941)	2.28E-21	6.66E-18
SIGLEC1	2.412 (2.092, 2.781)	8.70E-34	2.54E-30	2.636 (2.207, 3.15)	1.36E-26	3.98E-23
SIGLEC10	2.005 (1.689, 2.38)	1.93E-15	5.63E-12	2.053 (1.661, 2.537)	2.75E-11	8.04E-08
SIRPB1	1.886 (1.634, 2.177)	4.17E-18	1.22E-14	1.857 (1.55, 2.225)	1.85E-11	5.40E-08
SLAMF1	1.713 (1.521, 1.929)	6.56E-19	1.92E-15	1.735 (1.505, 2)	3.20E-14	9.37E-11
SLAMF7	1.57 (1.404, 1.755)	2.27E-15	6.62E-12	1.555 (1.353, 1.786)	4.56E-10	1.33E-06
SLAMF8	1.92 (1.683, 2.191)	3.69E-22	1.08E-18	1.795 (1.517, 2.122)	8.31E-12	2.43E-08

SNRPB2	1.445 (1.305, 1.599)	1.15E-12	3.36E-09	1.477 (1.307, 1.669)	4.06E-10	1.19E-06
SORCS2	2.023 (1.687, 2.426)	2.88E-14	8.41E-11	2.16 (1.707, 2.735)	1.53E-10	4.47E-07
SPINT1	5.254 (4.645, 5.944)	2.51E-153	7.35E-150	5.823 (4.911, 6.904)	2.21E-91	6.47E-88
SPON1	2.508 (2.199, 2.859)	5.95E-43	1.74E-39	2.502 (2.085, 3.002)	6.12E-23	1.79E-19
SPON2	3.123 (2.608, 3.74)	3.50E-35	1.02E-31	3.288 (2.627, 4.117)	2.82E-25	8.23E-22
ST6GAL1	2.179 (1.874, 2.534)	4.52E-24	1.32E-20	2.149 (1.784, 2.589)	7.97E-16	2.33E-12
TCN2	2.073 (1.733, 2.479)	1.51E-15	4.40E-12	2.169 (1.75, 2.687)	1.47E-12	4.31E-09
TCOF1	1.895 (1.594, 2.252)	4.18E-13	1.22E-09	1.959 (1.598, 2.402)	1.04E-10	3.03E-07
TFF3	1.72 (1.554, 1.904)	1.62E-25	4.72E-22	1.729 (1.499, 1.995)	6.11E-14	1.79E-10
TFPI2	1.536 (1.384, 1.703)	5.12E-16	1.50E-12	1.492 (1.319, 1.689)	2.11E-10	6.16E-07
TGFA	1.861 (1.635, 2.118)	4.86E-21	1.42E-17	1.814 (1.546, 2.129)	3.10E-13	9.07E-10
TGFBR2	2.007 (1.697, 2.375)	4.55E-16	1.33E-12	2.083 (1.678, 2.585)	2.79E-11	8.14E-08
THBS2	2.122 (1.833, 2.458)	9.34E-24	2.73E-20	2.003 (1.655, 2.425)	9.87E-13	2.89E-09
TIGIT	1.775 (1.58, 1.994)	3.77E-22	1.10E-18	1.719 (1.488, 1.987)	2.13E-13	6.22E-10
TIMD4	1.694 (1.449, 1.982)	4.21E-11	1.23E-07	1.925 (1.588, 2.334)	2.71E-11	7.91E-08
TIMP1	3.216 (2.711, 3.814)	5.14E-41	1.50E-37	3.323 (2.688, 4.11)	1.47E-28	4.31E-25
TMSB10	1.415 (1.289, 1.554)	3.09E-13	9.05E-10	1.465 (1.307, 1.643)	6.35E-11	1.85E-07
TNC	1.956 (1.7, 2.252)	7.72E-21	2.26E-17	2.098 (1.763, 2.498)	8.03E-17	2.35E-13
TNF	1.768 (1.604, 1.948)	1.21E-30	3.53E-27	1.782 (1.591, 1.997)	1.85E-23	5.41E-20
TNFRSF10A	2.175 (1.913, 2.472)	1.50E-32	4.40E-29	2.141 (1.817, 2.523)	8.79E-20	2.57E-16
TNFRSF10B	1.621 (1.538, 1.708)	1.88E-73	5.50E-70	1.635 (1.53, 1.746)	6.52E-48	1.91E-44

TNFRSF11B	2.764 (2.237, 3.416)	4.72E-21	1.38E-17	2.528 (1.929, 3.313)	1.78E-11	5.22E-08
TNFRSF12A	1.963 (1.644, 2.345)	9.66E-14	2.82E-10	2.014 (1.613, 2.516)	6.79E-10	1.99E-06
TNFRSF13B	1.997 (1.772, 2.251)	1.02E-29	2.97E-26	1.995 (1.701, 2.339)	1.79E-17	5.23E-14
TNFRSF14	2.449 (2.011, 2.984)	5.87E-19	1.71E-15	2.637 (2.062, 3.373)	1.11E-14	3.25E-11
TNFRSF17	2.396 (2.001, 2.87)	2.19E-21	6.40E-18	2.754 (2.221, 3.415)	2.62E-20	7.66E-17
TNFRSF19	2.007 (1.671, 2.411)	9.30E-14	2.72E-10	2.059 (1.639, 2.586)	5.53E-10	1.62E-06
TNFRSF1A	2.814 (2.344, 3.378)	1.29E-28	3.77E-25	2.862 (2.261, 3.622)	2.15E-18	6.29E-15
TNFRSF1B	1.869 (1.714, 2.039)	2.50E-45	7.31E-42	1.891 (1.705, 2.097)	1.85E-33	5.40E-30
TNFRSF4	3.318 (2.937, 3.749)	1.11E-82	3.25E-79	3.313 (2.837, 3.869)	8.55E-52	2.50E-48
TNFRSF6B	1.682 (1.527, 1.853)	7.73E-26	2.26E-22	1.612 (1.42, 1.831)	1.60E-13	4.68E-10
TNFRSF8	2.57 (2.302, 2.869)	2.81E-63	8.22E-60	2.659 (2.317, 3.053)	5.56E-44	1.63E-40
TNFRSF9	2.08 (1.891, 2.287)	1.91E-51	5.59E-48	2.141 (1.886, 2.43)	5.74E-32	1.68E-28
TNFSF13B	3.245 (2.832, 3.719)	3.09E-64	9.04E-61	3.833 (3.108, 4.728)	4.00E-36	1.17E-32
TNFSF8	2.455 (2.047, 2.944)	3.74E-22	1.09E-18	2.368 (1.876, 2.989)	4.17E-13	1.22E-09
TPK1	3.839 (2.929, 5.031)	1.87E-22	5.47E-19	4.133 (2.976, 5.738)	2.37E-17	6.94E-14
TPP1	1.628 (1.436, 1.845)	2.23E-14	6.53E-11	1.681 (1.447, 1.952)	1.01E-11	2.95E-08
TREM2	2.17 (1.929, 2.442)	5.24E-38	1.53E-34	2.065 (1.774, 2.402)	6.69E-21	1.96E-17
TTR	0.246 (0.182, 0.334)	2.54E-19	7.41E-16	0.196 (0.133, 0.288)	1.16E-16	3.40E-13
TXNDC15	2.845 (2.272, 3.561)	7.55E-20	2.21E-16	2.819 (2.099, 3.785)	5.53E-12	1.62E-08
TYMP	1.483 (1.333, 1.651)	5.74E-13	1.68E-09	1.585 (1.39, 1.807)	6.19E-12	1.81E-08
ULBP2	2.073 (1.76, 2.441)	2.46E-18	7.18E-15	2.151 (1.756, 2.635)	1.32E-13	3.86E-10

VCAM1	2.966 (2.368, 3.715)	3.09E-21	9.04E-18	2.95 (2.224, 3.913)	6.23E-14	1.82E-10
VSIG4	2.514 (2.188, 2.889)	1.16E-38	3.39E-35	2.516 (2.108, 3.003)	1.46E-24	4.28E-21
WARS	2.767 (2.341, 3.271)	7.94E-33	2.32E-29	2.893 (2.358, 3.548)	2.18E-24	6.38E-21
WFDC2	4.026 (3.672, 4.414)	2.13E-193	6.21E-190	4.481 (3.963, 5.066)	1.66E-126	4.86E-123
YAP1	2.803 (2.295, 3.423)	5.54E-24	1.62E-20	2.934 (2.251, 3.825)	1.78E-15	5.22E-12
ZBTB17	1.715 (1.548, 1.9)	6.12E-25	1.79E-21	1.725 (1.523, 1.954)	9.33E-18	2.73E-14

Supplementary Table 7. The GO, KEGG, and Reactome enrichment analyses of proteins related to ILD

	ONTOLOGY	Description	pvalue	p.adjust	Count
GO:0051249	BP	regulation of lymphocyte activation	5.49E-11	1.56E-07	48
GO:0002694	BP	regulation of leukocyte activation	2.56E-10	3.63E-07	53
GO:0050863	BP	regulation of T cell activation	4.49E-10	4.25E-07	40
GO:0032944	BP	regulation of mononuclear cell proliferation	1.63E-09	8.15E-07	34
GO:0050865	BP	regulation of cell activation	1.72E-09	8.15E-07	55
GO:0045321	BP	leukocyte activation	1.97E-09	8.15E-07	67
GO:0070663	BP	regulation of leukocyte proliferation	2.01E-09	8.15E-07	36
GO:0042110	BP	T cell activation	2.97E-09	1.05E-06	46
GO:0007159	BP	leukocyte cell-cell adhesion	4.21E-09	1.33E-06	42
GO:0001775	BP	cell activation	6.98E-09	1.98E-06	73
GO:0022409	BP	positive regulation of cell-cell adhesion	1.02E-08	2.63E-06	34
GO:0046649	BP	lymphocyte activation	1.31E-08	2.96E-06	55
GO:0050670	BP	regulation of lymphocyte proliferation	1.36E-08	2.96E-06	32
GO:0070661	BP	leukocyte proliferation	2.26E-08	4.57E-06	37
GO:0032943	BP	mononuclear cell proliferation	2.93E-08	5.54E-06	34
GO:0022407	BP	regulation of cell-cell adhesion	3.50E-08	6.20E-06	43
GO:1903037	BP	regulation of leukocyte cell-cell adhesion	3.97E-08	6.62E-06	37
GO:0030155	BP	regulation of cell adhesion	7.02E-08	1.10E-05	57
GO:0002696	BP	positive regulation of leukocyte activation	7.72E-08	1.15E-05	36
GO:0098609	BP	cell-cell adhesion	9.78E-08	1.39E-05	65
GO:0050867	BP	positive regulation of cell activation	1.38E-07	1.71E-05	37
GO:0046651	BP	lymphocyte proliferation	1.39E-07	1.71E-05	32
GO:0051251	BP	positive regulation of lymphocyte activation	1.39E-07	1.71E-05	32
GO:0050870	BP	positive regulation of T cell activation	1.76E-07	2.02E-05	28
GO:0042129	BP	regulation of T cell proliferation	1.78E-07	2.02E-05	26
GO:0042098	BP	T cell proliferation	2.24E-07	2.44E-05	27

GO:0002250	BP	adaptive immune response	3.13E-07	3.21E-05	41
GO:1903039	BP	positive regulation of leukocyte cell-cell adhesion	3.17E-07	3.21E-05	29
GO:0009607	BP	response to biotic stimulus	3.75E-07	3.67E-05	78
GO:0002684	BP	positive regulation of immune system process	5.15E-07	4.87E-05	59
GO:0006954	BP	inflammatory response	6.40E-07	5.85E-05	57
GO:0046718	BP	viral entry into host cell	6.71E-07	5.95E-05	22
GO:0044409	BP	entry into host	1.11E-06	9.52E-05	22
GO:0051707	BP	response to other organism	1.28E-06	0.000107	75
GO:0043207	BP	response to external biotic stimulus	1.53E-06	0.000124	75
GO:0051241	BP	negative regulation of multicellular organismal process	1.99E-06	0.000156	59
GO:1903131	BP	mononuclear cell differentiation	2.07E-06	0.000158	33
GO:0002683	BP	negative regulation of immune system process	2.72E-06	0.000202	36
GO:0051701	BP	biological process involved in interaction with host	2.80E-06	0.000202	23
GO:0044000	BP	movement in host	2.85E-06	0.000202	22
GO:0002521	BP	leukocyte differentiation	3.20E-06	0.000221	39
GO:0045785	BP	positive regulation of cell adhesion	3.65E-06	0.000246	39
GO:0050868	BP	negative regulation of T cell activation	3.91E-06	0.000258	18
GO:1903038	BP	negative regulation of leukocyte cell-cell adhesion	4.26E-06	0.000275	19
GO:0002507	BP	tolerance induction	4.97E-06	0.000313	8
GO:0002643	BP	regulation of tolerance induction	5.96E-06	0.00036	7
GO:0045087	BP	innate immune response	5.98E-06	0.00036	50
GO:0002460	BP	adaptive immune response based on somatic recombination of immune receptors built from immunoglobulin superfamily domains	6.73E-06	0.000398	28
GO:0098542	BP	defense response to other organism	8.79E-06	0.000508	60
GO:0034097	BP	response to cytokine	1.02E-05	0.000579	55

GO:0002819	BP	regulation of adaptive immune response	1.08E-05	0.000601	21
GO:0002822	BP	regulation of adaptive immune response based on somatic recombination of immune receptors built from immunoglobulin superfamily domains	1.12E-05	0.000608	20
GO:0032733	BP	positive regulation of interleukin-10 production	1.15E-05	0.000613	10
GO:0097529	BP	myeloid leukocyte migration	1.29E-05	0.000676	28
GO:0019221	BP	cytokine-mediated signaling pathway	1.37E-05	0.000708	38
GO:0019058	BP	viral life cycle	1.62E-05	0.000821	25
GO:0071675	BP	regulation of mononuclear cell migration	1.72E-05	0.000855	17
GO:0071674	BP	mononuclear cell migration	1.82E-05	0.000882	24
GO:0002367	BP	cytokine production involved in immune response	1.87E-05	0.000882	15
GO:0002718	BP	regulation of cytokine production involved in immune response	1.87E-05	0.000882	15
GO:0071677	BP	positive regulation of mononuclear cell migration	2.30E-05	0.001066	13
GO:0032613	BP	interleukin-10 production	2.37E-05	0.001066	12
GO:0032653	BP	regulation of interleukin-10 production	2.37E-05	0.001066	12
GO:0097530	BP	granulocyte migration	2.42E-05	0.001072	21
GO:0051250	BP	negative regulation of lymphocyte activation	2.72E-05	0.001186	19
GO:0002697	BP	regulation of immune effector process	2.95E-05	0.001267	31
GO:0070665	BP	positive regulation of leukocyte proliferation	3.54E-05	0.001497	21
GO:0002252	BP	immune effector process	3.79E-05	0.001578	43
GO:0071345	BP	cellular response to cytokine stimulus	3.93E-05	0.001613	49
GO:0002520	BP	immune system development	4.72E-05	0.001911	49
GO:0001816	BP	cytokine production	4.82E-05	0.001925	48
GO:0002687	BP	positive regulation of leukocyte migration	5.02E-05	0.00196	19
GO:0030595	BP	leukocyte chemotaxis	5.05E-05	0.00196	26
GO:0016032	BP	viral process	5.76E-05	0.00218	27

GO:0045582	BP	positive regulation of T cell differentiation	5.77E-05	0.00218	13
GO:0032946	BP	positive regulation of mononuclear cell proliferation	6.10E-05	0.002275	19
GO:0001817	BP	regulation of cytokine production	6.49E-05	0.002361	47
GO:0045621	BP	positive regulation of lymphocyte differentiation	6.50E-05	0.002361	14
GO:0032703	BP	negative regulation of interleukin-2 production	6.62E-05	0.002376	7
GO:0050900	BP	leukocyte migration	6.99E-05	0.002475	35
GO:0060326	BP	cell chemotaxis	7.09E-05	0.002481	29
GO:0009617	BP	response to bacterium	7.65E-05	0.002643	41
GO:0002449	BP	lymphocyte mediated immunity	7.90E-05	0.002698	25
GO:0006935	BP	chemotaxis	8.33E-05	0.002738	43
GO:0042330	BP	taxis	8.33E-05	0.002738	43
GO:0032945	BP	negative regulation of mononuclear cell proliferation	8.40E-05	0.002738	14
GO:0050672	BP	negative regulation of lymphocyte proliferation	8.40E-05	0.002738	14
GO:0048247	BP	lymphocyte chemotaxis	8.74E-05	0.002814	12
GO:0046006	BP	regulation of activated T cell proliferation	8.88E-05	0.002827	9
GO:1902105	BP	regulation of leukocyte differentiation	9.14E-05	0.002879	23
GO:0002443	BP	leukocyte mediated immunity	9.28E-05	0.002891	31
GO:0071356	BP	cellular response to tumor necrosis factor	0.00012	0.003692	21
GO:0007162	BP	negative regulation of cell adhesion	0.000121	0.0037	25
GO:0044403	BP	biological process involved in symbiotic interaction	0.000123	0.003722	24
GO:0002237	BP	response to molecule of bacterial origin	0.00013	0.003884	27
GO:0050798	BP	activated T cell proliferation	0.000134	0.003952	9
GO:0030098	BP	lymphocyte differentiation	0.000135	0.003954	26
GO:0034612	BP	response to tumor necrosis factor	0.000144	0.00415	22
GO:0022408	BP	negative regulation of cell-cell adhesion	0.00016	0.00459	20

GO:0002688	BP	regulation of leukocyte chemotaxis	0.000164	0.004636	16
GO:0002685	BP	regulation of leukocyte migration	0.000167	0.004636	23
GO:0070098	BP	chemokine-mediated signaling pathway	0.000169	0.004636	13
GO:0002700	BP	regulation of production of molecular mediator of immune response	0.000169	0.004636	18
GO:0030097	BP	hemopoiesis	0.00017	0.004636	44
GO:0070664	BP	negative regulation of leukocyte proliferation	0.000173	0.004677	14
GO:0032496	BP	response to lipopolysaccharide	0.000176	0.004712	26
GO:0071621	BP	granulocyte chemotaxis	0.000186	0.00478	17
GO:1902107	BP	positive regulation of leukocyte differentiation	0.000186	0.00478	17
GO:1903708	BP	positive regulation of hemopoiesis	0.000186	0.00478	17
GO:1990266	BP	neutrophil migration	0.000186	0.00478	17
GO:0071216	BP	cellular response to biotic stimulus	0.000193	0.004926	21
GO:0002286	BP	T cell activation involved in immune response	0.000203	0.005092	12
GO:0042130	BP	negative regulation of T cell proliferation	0.000203	0.005092	12
GO:0042102	BP	positive regulation of T cell proliferation	0.000211	0.00513	15
GO:0048534	BP	hematopoietic or lymphoid organ development	0.000214	0.00513	45
GO:0002456	BP	T cell mediated immunity	0.000215	0.00513	13
GO:0002824	BP	positive regulation of adaptive immune response based on somatic recombination of immune receptors built from immunoglobulin superfamily domains	0.000215	0.00513	13
GO:1990868	BP	response to chemokine	0.000215	0.00513	13
GO:1990869	BP	cellular response to chemokine	0.000215	0.00513	13
GO:0001818	BP	negative regulation of cytokine production	0.000222	0.005235	23

GO:0050866	BP	negative regulation of cell activation	0.000261	0.006119	21
GO:0002699	BP	positive regulation of immune effector process	0.000299	0.006949	22
GO:0016477	BP	cell migration	0.000321	0.007392	69
GO:0002548	BP	monocyte chemotaxis	0.000343	0.007736	13
GO:0002690	BP	positive regulation of leukocyte chemotaxis	0.000343	0.007736	13
GO:0050920	BP	regulation of chemotaxis	0.000344	0.007736	22
GO:0002695	BP	negative regulation of leukocyte activation	0.000348	0.007761	19
GO:1903706	BP	regulation of hemopoiesis	0.000366	0.008113	24
GO:0002706	BP	regulation of lymphocyte mediated immunity	0.00038	0.00829	17
GO:0050671	BP	positive regulation of lymphocyte proliferation	0.00038	0.00829	17
GO:1905523	BP	positive regulation of macrophage migration	0.000388	0.008388	6
GO:1905517	BP	macrophage migration	0.000396	0.008507	9
GO:0002703	BP	regulation of leukocyte mediated immunity	0.000404	0.008591	21
GO:0071219	BP	cellular response to molecule of bacterial origin	0.000406	0.008591	19
GO:0009967	BP	positive regulation of signal transduction	0.000419	0.008803	66
GO:0045619	BP	regulation of lymphocyte differentiation	0.000424	0.008824	16
GO:0002821	BP	positive regulation of adaptive immune response	0.000428	0.008824	13
GO:0007219	BP	Notch signaling pathway	0.00043	0.008824	12
GO:0046631	BP	alpha-beta T cell activation	0.000466	0.009495	15
GO:0030217	BP	T cell differentiation	0.000473	0.009509	19
GO:0032102	BP	negative regulation of response to external stimulus	0.000481	0.009509	30
GO:0002720	BP	positive regulation of cytokine production involved in immune response	0.000483	0.009509	10
GO:0032623	BP	interleukin-2 production	0.000483	0.009509	10
GO:0032663	BP	regulation of interleukin-2 production	0.000483	0.009509	10
GO:0045580	BP	regulation of T cell differentiation	0.000503	0.009826	14
GO:0002369	BP	T cell cytokine production	0.000537	0.010355	7

GO:0002724	BP	regulation of T cell cytokine production	0.000537	0.010355	7
GO:0040011	BP	locomotion	0.000543	0.010381	64
GO:0050922	BP	negative regulation of chemotaxis	0.000546	0.010381	9
GO:0097028	BP	dendritic cell differentiation	0.000576	0.010803	8
GO:2000403	BP	positive regulation of lymphocyte migration	0.000576	0.010803	8
GO:0030593	BP	neutrophil chemotaxis	0.000611	0.011388	14
GO:0070374	BP	positive regulation of ERK1 and ERK2 cascade	0.000626	0.011595	20
GO:0071222	BP	cellular response to lipopolysaccharide	0.000636	0.011698	18
GO:0001773	BP	myeloid dendritic cell activation	0.000663	0.01205	6
GO:0010818	BP	T cell chemotaxis	0.000663	0.01205	6
GO:0046634	BP	regulation of alpha-beta T cell activation	0.000677	0.012228	12
GO:0001525	BP	angiogenesis	0.000691	0.012386	36
GO:0030335	BP	positive regulation of cell migration	0.000703	0.012528	35
GO:0043410	BP	positive regulation of MAPK cascade	0.000796	0.014099	32
GO:0033209	BP	tumor necrosis factor-mediated signaling pathway	0.000827	0.014562	10
GO:0022612	BP	gland morphogenesis	0.00084	0.014703	12
GO:2000147	BP	positive regulation of cell motility	0.000914	0.015896	35
GO:0043408	BP	regulation of MAPK cascade	0.000921	0.015907	38
GO:0002440	BP	production of molecular mediator of immune response	0.000988	0.016968	18
GO:0048646	BP	anatomical structure formation involved in morphogenesis	0.00106	0.018041	51
GO:0046632	BP	alpha-beta T cell differentiation	0.001063	0.018041	10
GO:0030334	BP	regulation of cell migration	0.001106	0.018595	49
GO:0006909	BP	phagocytosis	0.001109	0.018595	19
GO:0050776	BP	regulation of immune response	0.001153	0.019216	44
GO:0048514	BP	blood vessel morphogenesis	0.001171	0.019298	38
GO:0046638	BP	positive regulation of alpha-beta T cell differentiation	0.001178	0.019298	7

GO:0001568	BP	blood vessel development	0.001178	0.019298	40
GO:0003203	BP	endocardial cushion morphogenesis	0.001246	0.019726	5
GO:0045671	BP	negative regulation of osteoclast differentiation	0.001246	0.019726	5
GO:0060142	BP	regulation of syncytium formation by plasma membrane fusion	0.001246	0.019726	5
GO:0072574	BP	hepatocyte proliferation	0.001246	0.019726	5
GO:0072575	BP	epithelial cell proliferation involved in liver morphogenesis	0.001246	0.019726	5
GO:0072576	BP	liver morphogenesis	0.001246	0.019726	5
GO:0070372	BP	regulation of ERK1 and ERK2 cascade	0.00129	0.020313	23
GO:0072678	BP	T cell migration	0.001351	0.021157	10
GO:0050778	BP	positive regulation of immune response	0.001369	0.021323	32
GO:0002274	BP	myeloid leukocyte activation	0.001472	0.022793	21
GO:0001819	BP	positive regulation of cytokine production	0.001494	0.023007	32
GO:0031295	BP	T cell costimulation	0.001512	0.023038	8
GO:0045670	BP	regulation of osteoclast differentiation	0.001512	0.023038	8
GO:0040012	BP	regulation of locomotion	0.001565	0.023719	51
GO:0040017	BP	positive regulation of locomotion	0.001638	0.024623	35
GO:0008593	BP	regulation of Notch signaling pathway	0.001668	0.024623	7
GO:0048246	BP	macrophage chemotaxis	0.001668	0.024623	7
GO:1905521	BP	regulation of macrophage migration	0.001668	0.024623	7
GO:0032101	BP	regulation of response to external stimulus	0.001674	0.024623	52
GO:0051384	BP	response to glucocorticoid	0.001677	0.024623	13
GO:0030316	BP	osteoclast differentiation	0.0017	0.024833	10
GO:0072676	BP	lymphocyte migration	0.001759	0.025571	14
GO:0008037	BP	cell recognition	0.001795	0.025819	15
GO:0034341	BP	response to interferon-gamma	0.001795	0.025819	15
GO:0035239	BP	tube morphogenesis	0.001846	0.026427	43
GO:0006898	BP	receptor-mediated endocytosis	0.001981	0.027854	20

GO:0071887	BP	leukocyte apoptotic process	0.001992	0.027854	13
GO:0035295	BP	tube development	0.001993	0.027854	49
GO:2000145	BP	regulation of cell motility	0.001993	0.027854	49
GO:0045595	BP	regulation of cell differentiation	0.001995	0.027854	56
GO:0031294	BP	lymphocyte costimulation	0.002008	0.027894	8
GO:0043122	BP	regulation of I-kappaB kinase/NF-kappaB signaling	0.002066	0.028562	16
GO:0002523	BP	leukocyte migration involved in inflammatory response	0.002109	0.028871	5
GO:0003177	BP	pulmonary valve development	0.002109	0.028871	5
GO:0001944	BP	vasculature development	0.002126	0.028963	40
GO:0042742	BP	defense response to bacterium	0.002227	0.030192	20
GO:0008285	BP	negative regulation of cell population proliferation	0.002335	0.031505	36
GO:0043123	BP	positive regulation of I-kappaB kinase/NF-kappaB signaling	0.002412	0.032396	14
GO:0000165	BP	MAPK cascade	0.002562	0.034253	39
GO:0002709	BP	regulation of T cell mediated immunity	0.002615	0.034582	10
GO:0035710	BP	CD4-positive, alpha-beta T cell activation	0.002615	0.034582	10
GO:0031100	BP	animal organ regeneration	0.002624	0.034582	8
GO:0006959	BP	humoral immune response	0.002893	0.037961	21
GO:0070371	BP	ERK1 and ERK2 cascade	0.003006	0.039262	23
GO:0032689	BP	negative regulation of interferon-gamma production	0.003108	0.040406	7
GO:0071706	BP	tumor necrosis factor superfamily cytokine production	0.003201	0.041241	15
GO:1903555	BP	regulation of tumor necrosis factor superfamily cytokine production	0.003201	0.041241	15
GO:0010628	BP	positive regulation of gene expression	0.003253	0.041716	49
GO:0042104	BP	positive regulation of activated T cell proliferation	0.003338	0.042422	5
GO:0060412	BP	ventricular septum morphogenesis	0.003338	0.042422	5
GO:0033993	BP	response to lipid	0.003443	0.043258	40

GO:0031639	BP	plasminogen activation	0.00345	0.043258	6
GO:2000406	BP	positive regulation of T cell migration	0.00345	0.043258	6
GO:0006897	BP	endocytosis	0.003495	0.043639	31
GO:0002573	BP	myeloid leukocyte differentiation	0.003535	0.043863	16
GO:0045597	BP	positive regulation of cell differentiation	0.003544	0.043863	38
GO:0048729	BP	tissue morphogenesis	0.003603	0.044399	26
GO:0051093	BP	negative regulation of developmental process	0.003712	0.045541	37
GO:0009986	CC	cell surface	1.57E-11	4.55E-09	81
GO:0009897	CC	external side of plasma membrane	2.53E-07	3.66E-05	43
GO:0098552	CC	side of membrane	7.03E-06	0.00068	46
GO:0005887	CC	integral component of plasma membrane	0.000258	0.017456	60
GO:0031226	CC	intrinsic component of plasma membrane	0.000301	0.017456	64
GO:0043202	CC	lysosomal lumen	0.000728	0.035168	14
GO:0030312	CC	external encapsulating structure	0.00107	0.038782	40
GO:0031012	CC	extracellular matrix	0.00107	0.038782	40
GO:0005581	CC	collagen trimer	0.001277	0.041143	9
GO:0038023	MF	signaling receptor activity	4.88E-10	7.23E-08	67
GO:0060089	MF	molecular transducer activity	4.88E-10	7.23E-08	67
GO:0004888	MF	transmembrane signaling receptor activity	6.87E-07	6.03E-05	48
GO:0005035	MF	death receptor activity	8.15E-07	6.03E-05	8
GO:0001618	MF	virus receptor activity	1.55E-06	7.64E-05	17
GO:0140272	MF	exogenous protein binding	1.55E-06	7.64E-05	17
GO:0005125	MF	cytokine activity	7.38E-06	0.000312	30
GO:0030545	MF	signaling receptor regulator activity	0.000133	0.004928	46
GO:0030546	MF	signaling receptor activator activity	0.00018	0.005926	44
GO:0008009	MF	chemokine activity	0.000288	0.008322	12
GO:0048018	MF	receptor ligand activity	0.000309	0.008322	43
GO:0030246	MF	carbohydrate binding	0.000338	0.008327	23
GO:0005126	MF	cytokine receptor binding	0.001096	0.023851	27
GO:0042379	MF	chemokine receptor binding	0.001128	0.023851	12
GO:0019955	MF	cytokine binding	0.001979	0.039043	15

hsa04060	KEGG	Cytokine-cytokine receptor interaction	1.67E-10	3.11E-08	50
hsa04061	KEGG	Viral protein interaction with cytokine and cytokine receptor	1.34E-07	1.25E-05	24
R-HSA-5669034	Reactome	TNFs bind their physiological receptors	4.95E-11	1.87E-08	15
R-HSA-6783783	Reactome	Interleukin-10 signaling	7.18E-09	1.36E-06	17
R-HSA-5668541	Reactome	TNFR2 non-canonical NF-kB pathway	5.43E-08	6.84E-06	18
R-HSA-1280215	Reactome	Cytokine Signaling in Immune system	3.82E-06	0.00031	52
R-HSA-198933	Reactome	Immunoregulatory interactions between a Lymphoid and a non-Lymphoid cell	4.10E-06	0.00031	24
R-HSA-380108	Reactome	Chemokine receptors bind chemokines	0.00019	0.011973	11
R-HSA-216083	Reactome	Integrin cell surface interactions	0.000404	0.021799	13
R-HSA-1474244	Reactome	Extracellular matrix organization	0.000545	0.023728	27
R-HSA-1280218	Reactome	Adaptive Immune System	0.000565	0.023728	37

Supplementary Table 8. Baseline characteristics and covariate balance after three-group propensity-score matching

Characteristic	Prevalent ILD (n = 234)	Future incident ILD (n = 234)	No recorded ILD (n = 234)	SMD P vs I	SMD P vs N	SMD I vs N
**Demographic characteristics**						
Age, years, median [IQR]	63.0 [58.2, 66.0]	63.0 [57.2, 66.0]	63.5 [58.0, 67.0]	0.013	0.079	0.090
Female, n (%)	100 (42.7)	100 (42.7)	100 (42.7)	0.000	0.000	0.000
Male, n (%)	134 (57.3)	134 (57.3)	134 (57.3)	0.000	0.000	0.000
**Ethnicity, n (%)**						
White	218 (93.2)	214 (91.5)	222 (94.9)	0.068	0.068	**0.137**
Non-White	14 (6.0)	17 (7.3)	11 (4.7)	0.054	0.054	**0.108**
**Smoking status, n (%)**						
Never	90 (38.5)	86 (36.8)	77 (32.9)	0.035	**0.113**	0.079
Previous	116 (49.6)	115 (49.1)	130 (55.6)	0.009	**0.122**	**0.131**
Current	28 (12.0)	33 (14.1)	27 (11.5)	0.063	0.014	0.077
**BMI category, n (%)**						
Underweight (<18.5 kg/m2)	2 (0.9)	1 (0.4)	3 (1.3)	0.047	0.052	**0.106**
Normal weight (18.5 to <25 kg/m2)	72 (30.8)	70 (29.9)	69 (29.5)	0.019	0.028	0.010
Overweight (25 to <30 kg/m2)	83 (35.5)	83 (35.5)	73 (31.2)	0.000	0.088	0.086
Obesity (>=30 kg/m2)	77 (32.9)	80 (34.2)	89 (38.0)	0.027	**0.114**	0.085
**Comorbidities and ILD-related characteristics**						
Hypertension, n (%)	160 (68.4)	164 (70.1)	164 (70.1)	0.037	0.035	0.000
Diabetes, n (%)	26 (11.1)	31 (13.2)	21 (9.0)	0.068	0.081	**0.162**
Cardiovascular disease, n (%)	57 (24.4)	65 (27.8)	64 (27.4)	0.085	0.087	0.014
GERD before baseline, n (%)	34 (14.5)	34 (14.5)	44 (18.8)	0.000	**0.141**	**0.151**
Substance-related disorder before baseline, n (%)	8 (3.4)	9 (3.8)	8 (3.4)	0.025	0.000	0.033

**Systemic corticosteroid use at baseline, n (%)**						
Recorded use	40 (17.1)	29 (12.4)	34 (14.5)	**0.148**	0.093	**0.118**
No recorded use in available medication entry	186 (79.5)	198 (84.6)	192 (82.1)	**0.129**	0.060	0.061
**Selected ILD-associated/pneumotoxic medication use, n (%)**						
Recorded use	25 (10.7)	18 (7.7)	16 (6.8)	**0.109**	**0.166**	0.047
No recorded use in available medication entry	201 (85.9)	209 (89.3)	210 (89.7)	0.093	0.096	0.010
**Baseline lung function**						
FEV1, % predicted, median [IQR]	75.8 [67.6, 90.4]	80.3 [67.9, 93.2]	83.9 [69.5, 93.2]	**0.103**	**0.153**	0.047
FVC, % predicted, median [IQR]	84.5 [72.9, 97.0]	85.9 [74.5, 96.4]	88.4 [77.1, 98.2]	**0.103**	**0.143**	0.038
FEV1/FVC, median [IQR]	0.741 [0.684, 0.793]	0.737 [0.698, 0.777]	0.738 [0.691, 0.774]	0.001	0.001	0.000

Supplementary Table 9. Differential plasma protein expression between CTD-ILD and non-CTD-ILD

	Log2FC	AveExpr	t	P.Value	adj.P.Val
CXCL13	0.638	0.070	8.618	7.01E-18	2.70E-16
CCL7	0.634	0.061	8.576	1.01E-17	2.70E-16
PDCD1	0.447	0.039	8.690	3.73E-18	2.50E-16
TNFRSF4	0.431	0.028	9.139	6.51E-20	8.73E-18
RRM2	0.428	0.064	7.138	9.60E-13	1.84E-11
NOS3	0.399	0.044	6.223	4.91E-10	6.09E-09
VSIG4	0.374	0.028	7.015	2.33E-12	3.90E-11
LGALS9	0.369	0.018	8.577	9.98E-18	2.70E-16
IGFBP2	0.334	-0.052	3.833	0.000126661	0.00046
TNFRSF6B	0.331	0.044	4.734	2.21E-06	1.32E-05
CD300E	0.329	0.032	6.207	5.46E-10	6.09E-09
TYMP	0.306	0.006	3.833	0.000126939	0.00046
TNFSF13B	0.288	0.015	8.188	2.72E-16	6.09E-15
LTA4H	0.255	0.085	3.465	0.000529942	0.00142
BAP18	0.254	0.057	3.469	0.000522124	0.00142
CD27	0.252	0.029	4.953	7.31E-07	5.16E-06
MDK	0.247	0.042	3.935	8.33E-05	0.000349
IL2RA	0.241	0.030	4.852	1.23E-06	7.83E-06
CD83	0.233	0.017	5.445	5.19E-08	4.64E-07
CCL18	0.229	0.043	2.614	0.008938592	0.017698
POLR2F	0.228	0.034	5.293	1.20E-07	9.50E-07
ULBP2	0.213	0.007	4.498	6.88E-06	3.69E-05
CSF1	0.212	0.022	6.213	5.24E-10	6.09E-09
IGFBP4	0.204	0.037	3.637	0.000275793	0.000786
CD80	0.199	0.022	5.467	4.59E-08	4.39E-07
LMNB2	0.199	0.014	6.378	1.81E-10	2.70E-09
OGFR	0.199	0.010	4.892	1.00E-06	6.71E-06
CKAP4	0.196	0.028	5.767	8.10E-09	8.35E-08
PILRA	0.193	-0.022	3.675	0.000238486	0.000726
SDC1	0.191	0.027	3.910	9.23E-05	0.000375
IL18	0.190	0.019	3.273	0.001063255	0.002659
ST6GAL1	0.190	0.009	5.142	2.73E-07	2.03E-06
WFDC2	0.189	0.064	3.711	0.000206545	0.00066
HIP1R	0.178	0.042	3.753	0.000174575	0.000585
ALPP	0.178	0.136	1.191	0.23380697	0.295567
TIGIT	0.176	0.020	4.728	2.27E-06	1.32E-05
LAMP3	0.174	0.047	2.450	0.014282689	0.025863
CLEC6A	0.172	0.012	3.033	0.002422511	0.005567
MMP12	0.171	0.040	2.423	0.015378015	0.027475
ADA2	0.171	0.025	3.271	0.001071638	0.002659

CCL20	0.170	0.148	1.558	0.119245334	0.168199
MZB1	0.169	0.034	2.647	0.008114986	0.016476
MAMDC2	0.168	0.014	4.548	5.44E-06	3.04E-05
CD5	0.165	0.012	3.855	0.000115785	0.000443
CLEC5A	0.161	0.002	4.352	1.35E-05	6.96E-05
TNFRSF10 A	0.161	0.019	3.823	0.000132204	0.000466
DSC2	0.159	0.016	3.768	0.000164654	0.000566
CD300C	0.159	0.013	3.652	0.000260747	0.000776
TNFRSF14	0.157	0.022	4.155	3.26E-05	0.000145
SHISA5	0.156	0.016	5.404	6.56E-08	5.49E-07
SIGLEC10	0.154	0.011	3.711	0.000206734	0.00066
PLAUR	0.150	0.019	4.147	3.37E-05	0.000146
ADAM8	0.149	0.001	4.218	2.47E-05	0.000118
LAYN	0.145	0.017	3.351	0.000806998	0.00208
EDA2R	0.142	0.017	2.510	0.012076786	0.022476
TCOF1	0.141	0.012	4.220	2.45E-05	0.000118
LTBP2	0.139	0.007	3.073	0.00211809	0.005068
SCARB2	0.136	0.028	3.410	0.000649467	0.001706
TGFA	0.133	0.067	2.321	0.020273536	0.03313
CD79B	0.133	0.030	2.408	0.016031506	0.027673
CWC15	0.130	0.106	2.014	0.043973034	0.067729
KRT19	0.118	0.079	1.443	0.149043164	0.203794
LRG1	0.118	0.007	4.161	3.17E-05	0.000145
CDCP1	0.118	0.044	1.684	0.092195437	0.131428
NECTIN4	0.115	0.023	3.010	0.00261105	0.005831
ROBO1	0.115	0.011	3.876	0.000106127	0.000418
SORCS2	0.115	0.008	2.609	0.009081934	0.017698
SEZ6L2	0.114	0.000	3.641	0.000272117	0.000786
PSAP	0.112	0.018	3.683	0.000230789	0.000719
PLAU	0.110	0.007	3.061	0.002208151	0.005191
EFEMP1	0.109	0.011	2.608	0.009113306	0.017698
DDAH1	0.107	0.037	1.961	0.049882927	0.075958
TNC	0.107	0.017	1.841	0.065648523	0.096669
TCN2	0.106	0.008	2.840	0.004511068	0.009445
ANGPT2	0.106	0.028	2.208	0.027274702	0.04351
COL18A1	0.106	0.002	3.487	0.000488525	0.001364
RSPO3	0.106	0.010	2.898	0.003760265	0.008127
NPC2	0.106	0.018	2.985	0.002833437	0.006224
CCN3	0.105	0.019	2.358	0.018370452	0.030391
PLA2G10	0.105	0.007	1.418	0.156256825	0.211499
ADM	0.102	-0.007	2.580	0.009870068	0.018894

TNFRSF12A	0.100	0.021	2.159	0.030883585	0.048687
CHCHD10	0.099	0.019	2.128	0.033370693	0.051996
LILRA5	0.099	0.002	2.407	0.016108186	0.027673
FCER2	0.096	0.002	1.542	0.123077666	0.171796
CD300A	0.096	0.006	3.029	0.002451121	0.005567
MSLN	0.095	0.094	1.297	0.194520926	0.253066
CLEC14A	0.094	0.009	2.387	0.016999358	0.028834
SCGB3A2	0.092	0.048	0.874	0.382076062	0.457127
CAPG	0.092	-0.009	1.261	0.207187071	0.26441
ACVRL1	0.091	0.017	2.815	0.004886149	0.010073
CD22	0.090	0.008	1.693	0.090399206	0.130388
GDF15	0.086	0.068	1.373	0.169632347	0.227307
PLA2G15	0.085	0.015	2.555	0.010618265	0.02004
FGA	0.079	0.010	2.360	0.018272694	0.030391
TNFRSF19	0.078	0.017	1.927	0.053968809	0.080354
PTPRN2	0.077	-0.001	1.936	0.052933181	0.079697
RNASET2	0.074	0.014	2.229	0.025796862	0.041648
ATRAID	0.074	0.022	2.410	0.01595161	0.027673
SCGB3A1	0.068	0.004	2.487	0.012877072	0.023637
MMP7	0.065	-0.022	1.081	0.279865041	0.34724
CSF2	0.062	0.010	1.693	0.090493366	0.130388
PIGR	0.061	0.036	1.300	0.19364404	0.253066
FLT3LG	0.054	0.001	1.263	0.206484356	0.26441
TNFRSF10B	0.047	0.040	0.920	0.357626327	0.431729
CCL17	0.039	0.005	0.353	0.724167481	0.770146
CLEC3B	0.032	-0.004	1.448	0.147690741	0.203794
PLAT	0.028	-0.013	0.396	0.691938943	0.74774
ICAM3	0.022	0.008	0.707	0.479544469	0.553957
EPS8L2	0.021	0.013	0.555	0.578685246	0.646199
KLKB1	0.018	-0.004	0.693	0.488348293	0.559305
NRCAM	0.008	0.012	0.222	0.824532854	0.856491
SCGB1A1	0.007	-0.058	0.095	0.924101803	0.938598
CLC	0.004	0.073	0.049	0.960634197	0.960634
SPINT1	-0.002	0.013	-0.058	0.954021106	0.960634
COL24A1	-0.006	-0.050	-0.095	0.924589418	0.938598
FUT3_FUT5	-0.007	-0.001	-0.117	0.906844809	0.934748
SELPLG	-0.015	-0.021	-0.434	0.664307003	0.729649
AREG	-0.018	0.069	-0.324	0.746195477	0.787324
NPL	-0.020	0.016	-0.371	0.710351319	0.761497
BPIFB1	-0.020	-0.005	-0.247	0.804694527	0.842415
SERPINA4	-0.021	-0.003	-0.852	0.394229003	0.467493
GALNT5	-0.023	-0.001	-0.793	0.42779853	0.502851

ICAM5	-0.023	-0.031	-0.408	0.68321611	0.744317
CEACAM6	-0.028	0.027	-0.634	0.526086584	0.59742
CXCL17	-0.031	0.037	-0.473	0.63589933	0.704219
PRSS8	-0.032	0.027	-0.569	0.569142322	0.640883
ROBO2	-0.034	0.003	-1.174	0.240343216	0.300991
POSTN	-0.040	0.006	-1.011	0.311855787	0.383382
LY96	-0.041	0.019	-0.948	0.343079607	0.417933
CCL22	-0.046	0.019	-0.719	0.472332525	0.55037
EGFR	-0.058	-0.004	-2.850	0.00437618	0.009308
FCRL5	-0.096	0.007	-1.317	0.187957328	0.249369
SFTPD	-0.256	0.008	-3.116	0.001836656	0.004475

Supplementary Table 10. Model evaluation for incident ILD

Outcome	Model	AUC	Sensitivity	Specificity
ALL incident ILD	Proteins	0.8586 (0.8275-0.8897)	0.7252 (0.7167-0.7337)	0.845 (0.8381-0.8519)
	Clinical	0.7844 (0.7491-0.8196)	0.7863 (0.7785-0.7941)	0.6611 (0.6521-0.6701)
	Proteins+Clinical	0.8914 (0.8641-0.9187)	0.7787 (0.7708-0.7866)	0.8507 (0.8439-0.8575)
5-year incident ILD	Proteins	0.8987 (0.8467-0.9506)	0.8974 (0.8917-0.9032)	0.8301 (0.823-0.8373)
	Clinical	0.8467 (0.7972-0.8962)	0.7568 (0.7485-0.765)	0.7763 (0.7683-0.7843)
	Proteins+Clinical	0.9385 (0.9065-0.9704)	0.9333 (0.9286-0.9381)	0.8618 (0.8552-0.8684)
10-year incident ILD	Proteins	0.8898 (0.8544-0.9253)	0.8462 (0.8392-0.8531)	0.8296 (0.8224-0.8368)
	Clinical	0.796 (0.7515-0.8404)	0.725 (0.7165-0.7335)	0.7455 (0.7372-0.7538)
	Proteins+Clinical	0.9076 (0.8811-0.9341)	0.9231 (0.918-0.9282)	0.735 (0.7265-0.7434)

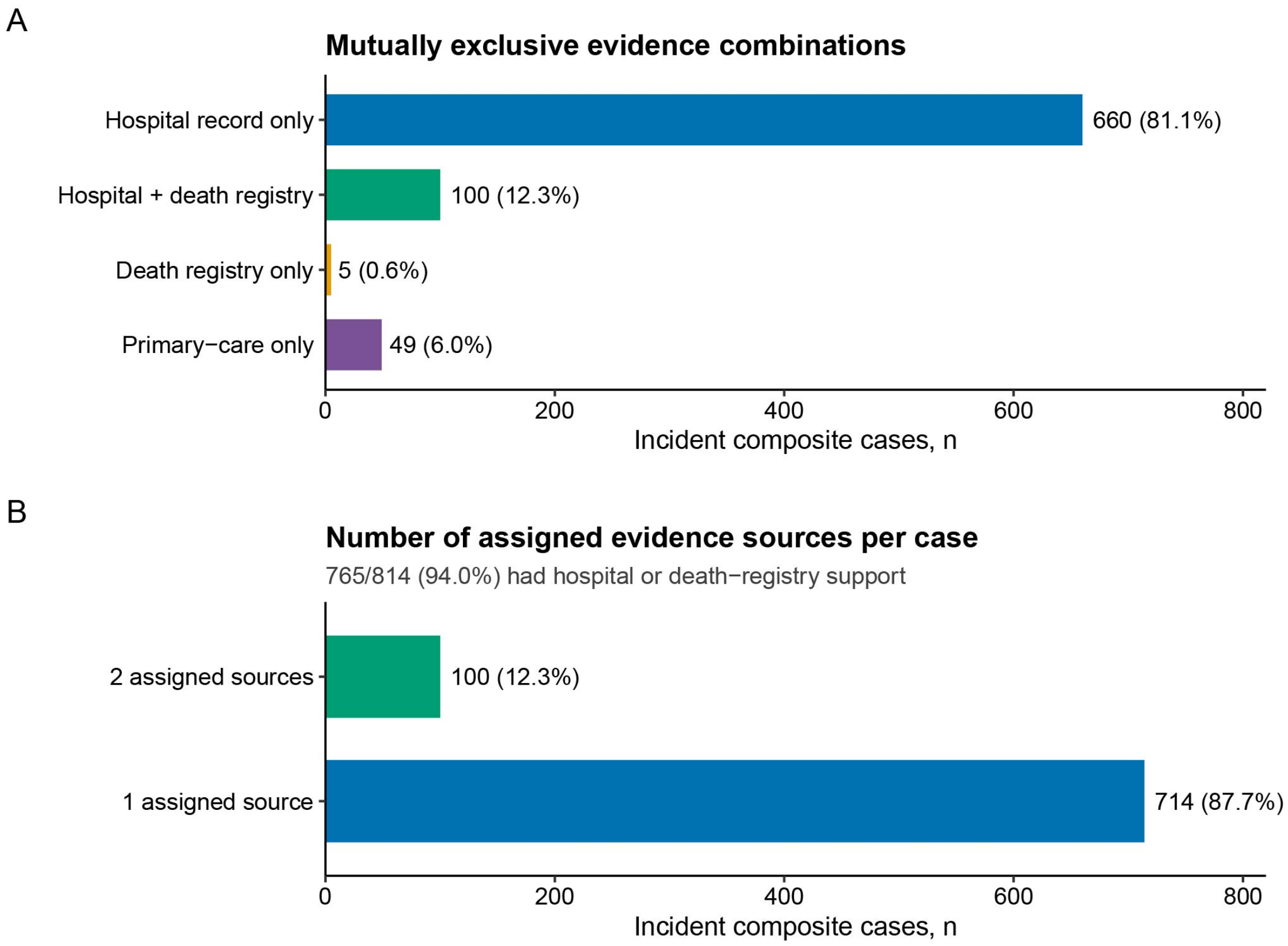

Supplementary Figure 1. Evidence sources for incident composite ILD ascertainment

(A) Mutually exclusive combinations of assigned evidence sources.

(B) Number of assigned sources per case. Bar labels indicate counts and percentages. Death-registry evidence refers to the underlying cause of death. Primary-care-only evidence was inferred from the absence of supporting records in the available hospital, death-registry, and self-report fields, rather than directly verified. Source concordance does not constitute gold-standard diagnostic validation.

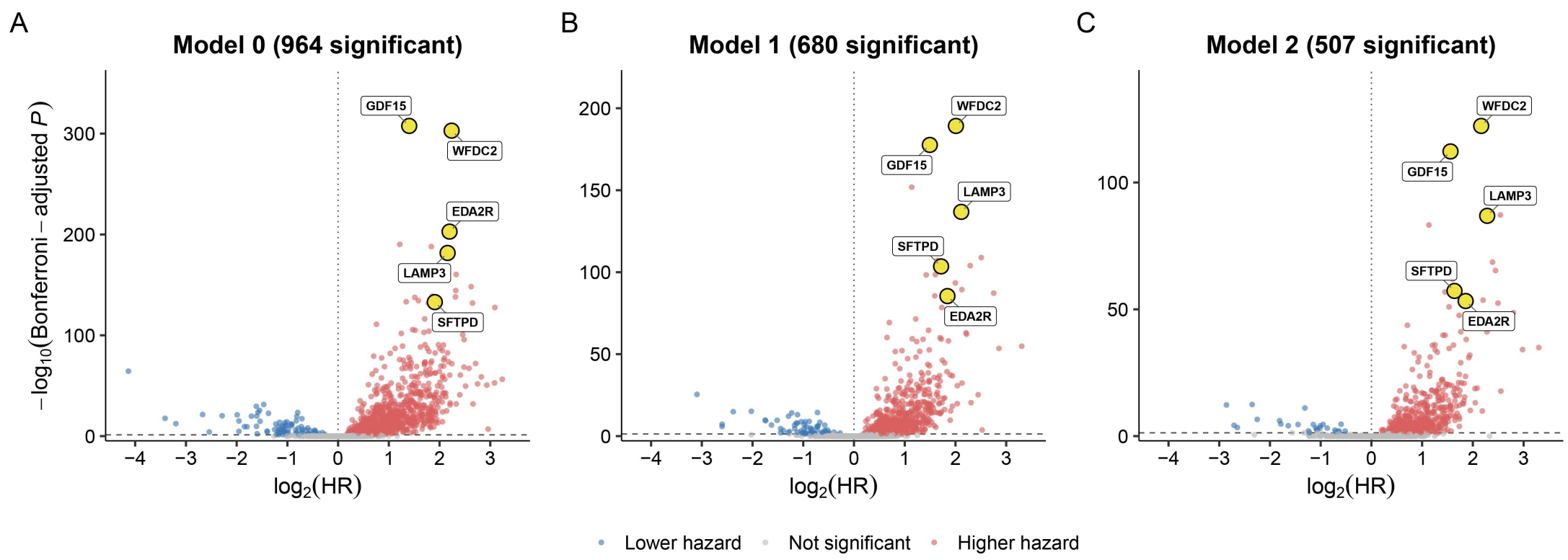

Supplementary Figure 2. Volcano plots of protein–ILD associations in the sensitivity analysis

(A-C). Volcano plots showing the ILD associated proteins. Red and blue indicate significant associations with higher and lower hazard, respectively; gray indicates nonsignificant associations. Yellow points highlight GDF15, LAMP3, SFTPD, EDA2R, and WFDC2. Reference lines indicate HR = 1 and Bonferroni-adjusted P = 0.05. Panel titles indicate significant protein counts; y-axis scales differ between (A) Model 0: unadjusted. (B) Model 1: adjusted for age, sex, BMI category, Townsend deprivation, and ethnicity and (C) Model 2: additionally adjusted for smoking, alcohol consumption, physical activity, hypertension, diabetes, and air pollution score.

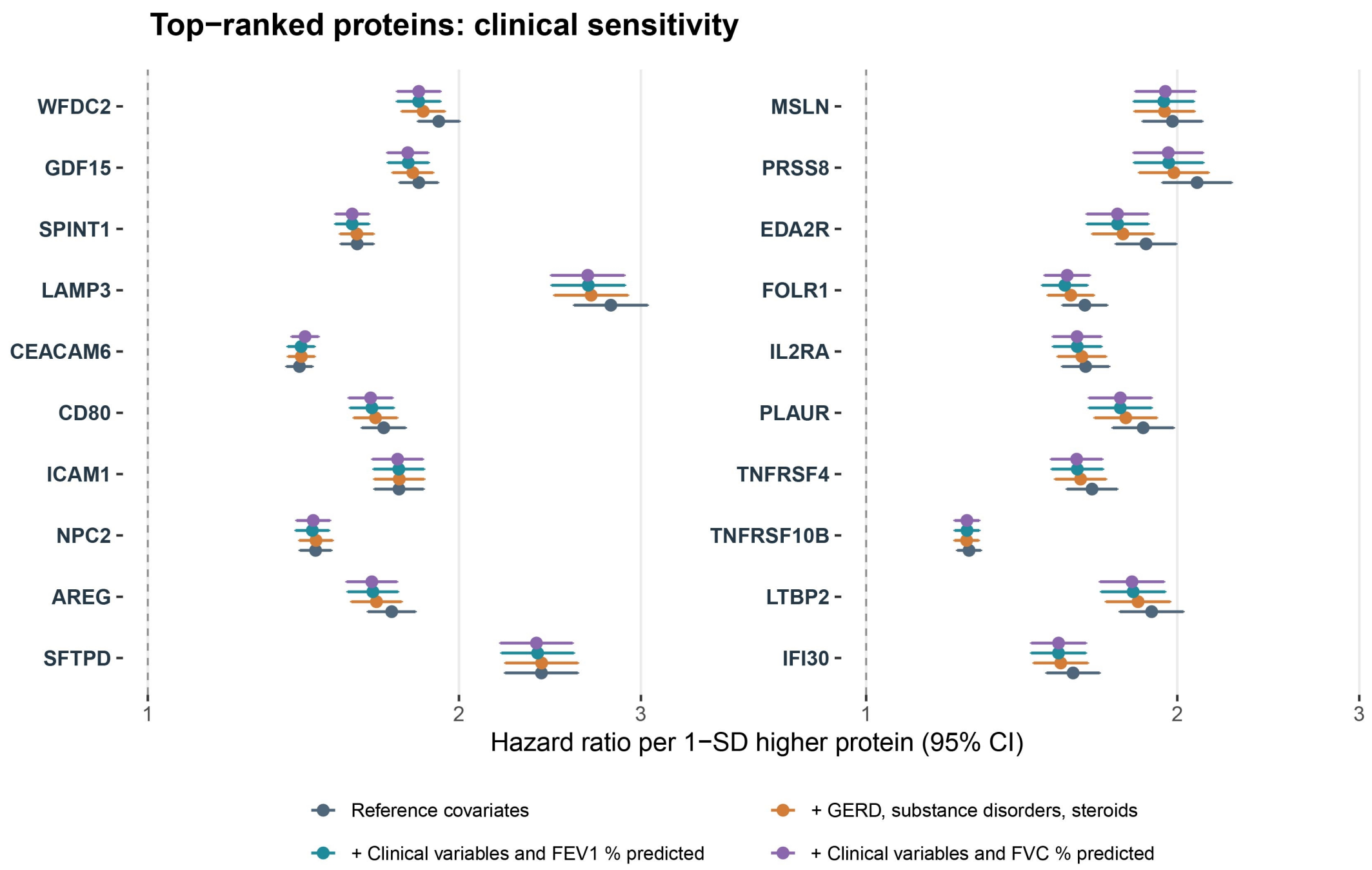

Supplementary Figure 3. Sensitivity of protein-ILD associations to additional clinical adjustment

Forest plot show the hazard ratios per 1-SD higher baseline protein level and 95% confidence intervals from Cox regression models. Models include reference covariates, additional adjustment for baseline GERD, substance-related disorders and systemic corticosteroid use (orange), and further adjustment for FEV1 % predicted (teal) or FVC % predicted (purple). Percent-predicted values were calculated using reference equations incorporating age, sex and height; FEV1 and FVC entered separate models. The dashed vertical line indicates a hazard ratio of 1.

Abbreviations ILD, interstitial lung disease; GERD, gastroesophageal reflux disease; FEV1, forced expiratory volume in 1 second; FVC, forced vital capacity.

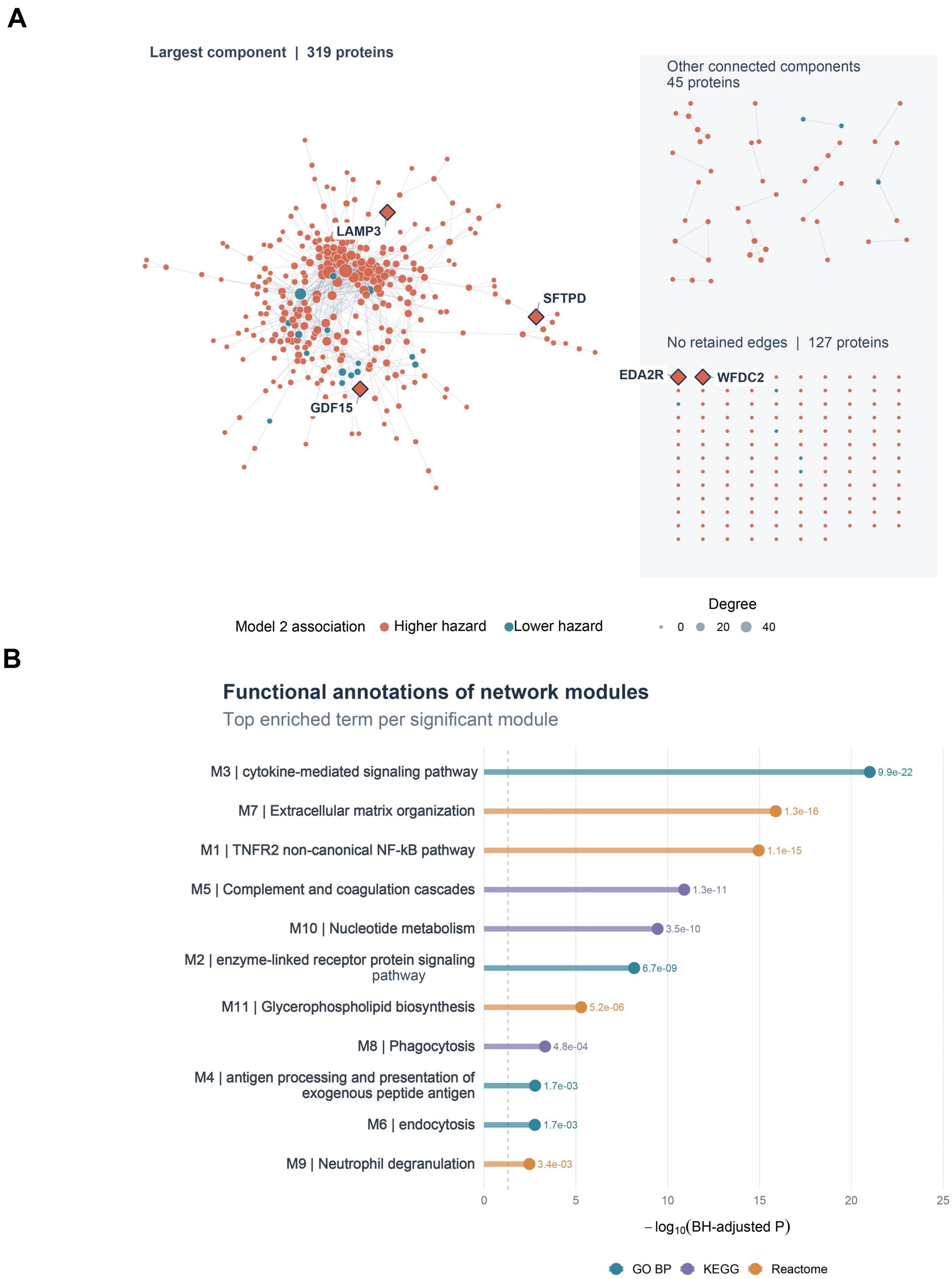

Supplementary Figure 4. STRING functional network and module enrichment of ILD-associated proteins

(A) Functional association network at a STRING confidence score ≥0.70. Nodes represent proteins, with size indicating degree and color indicating higher (orange) or lower (blue) ILD hazard in Model 2. Diamonds highlight GDF15, LAMP3, SFTPD, EDA2R, and WFDC2. The largest connected component, other connected components, and nodes without retained edges are displayed separately. Edges represent functional associations, not necessarily direct physical interactions.

(B) The most significantly enriched term for each significant network module. Colors indicate annotation databases; horizontal positions and numerical labels indicate $-\log_{10}$(BH-adjusted P) and BH-adjusted P values, respectively. Enrichment used selected STRING-mapped genes as the background, with BH correction across all module–term tests.

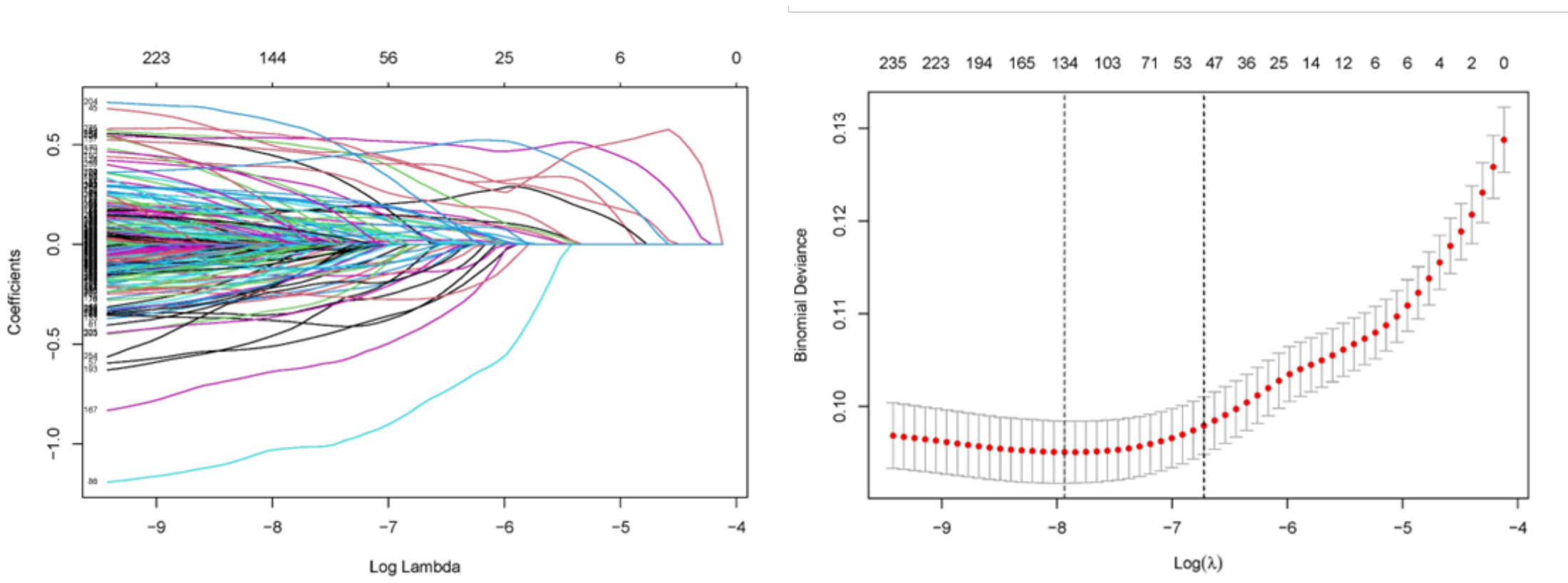

Supplementary Figure 5. LASSO (L1 regularization) protein screening

(A) Coefficient path plot of the LASSO regression. The coefficient paths of the LASSO regression model for each predictor are shown as functions of the regularization parameter (lambda).

(B) Cross-validation curve of the LASSO regression. The line plot presents the mean cross-validated error of the LASSO regression model as a function of the regularization parameter (lambda). The vertical dashed line indicates the optimal lambda from 5-fold cross-validation.

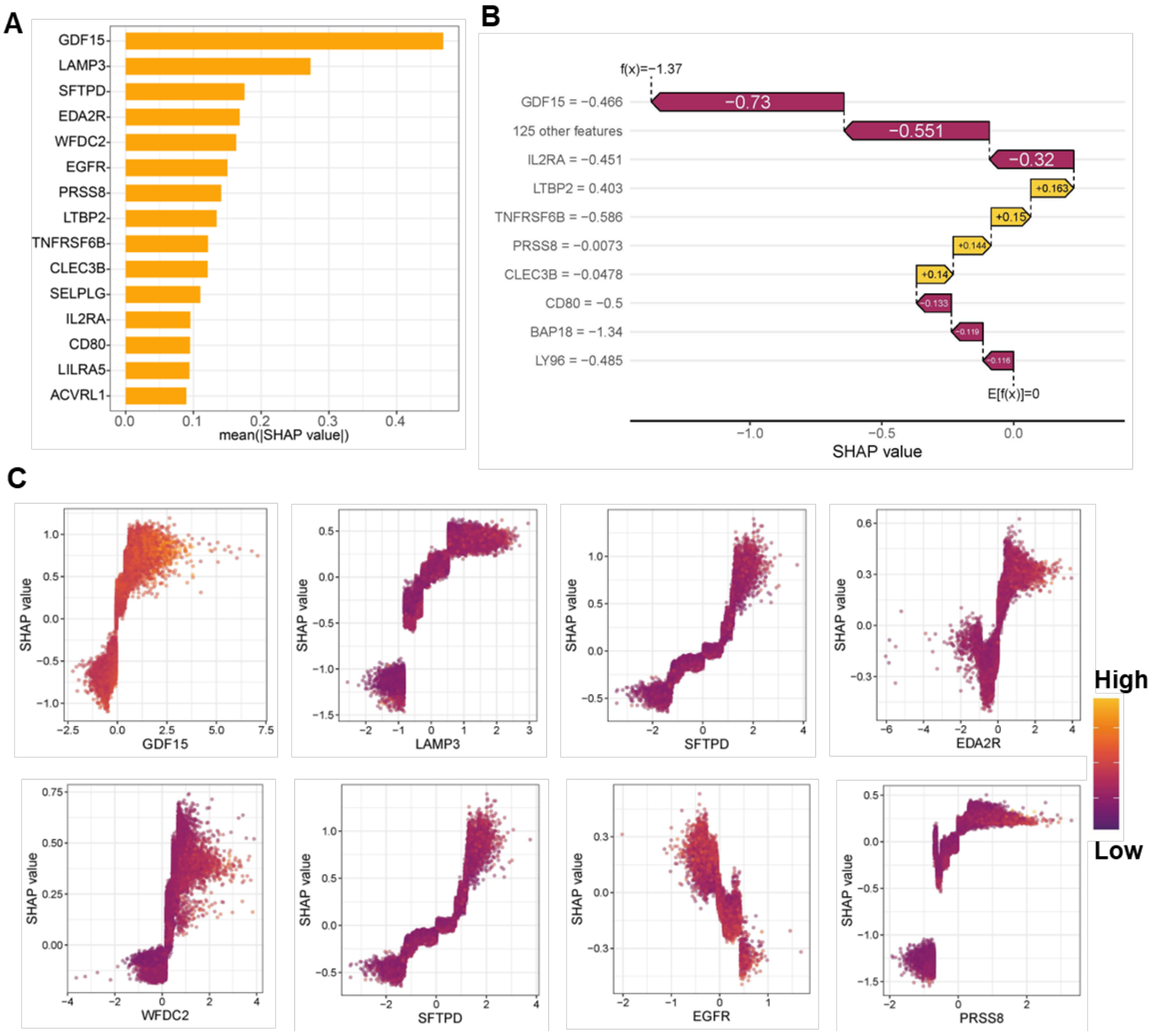

Supplementary Figure 6. Protein model explanation by the SHAP method in ILD

(A) Summary bar plot of the SHAP.

(B) Waterfall plot showing the individual contributions of each feature to the risk of incident ILD. This waterfall plot shows the cumulative effects of individual features on the overall risk of developing ILD. Each bar in the plot represents a specific feature, with the height of the bar indicating the change in the risk score attributable to that feature. The plot starts with a baseline risk (indicated by the dashed line) and shows how the risk accumulates as each feature is added sequentially. The red bars represent the features that decrease the risk of ILD. The yellow bars represent the features that increase the risk of developing ILD. The final bar shows the total change in risk after considering all features.

(C) SHAP dependence plot. Each dependence plot shows the effect of a single feature on the output of the prediction model, where each dot represents a patient.

Abbreviations SHAP: SHapley Additive exPlanations. ILD: Interstitial lung disease.

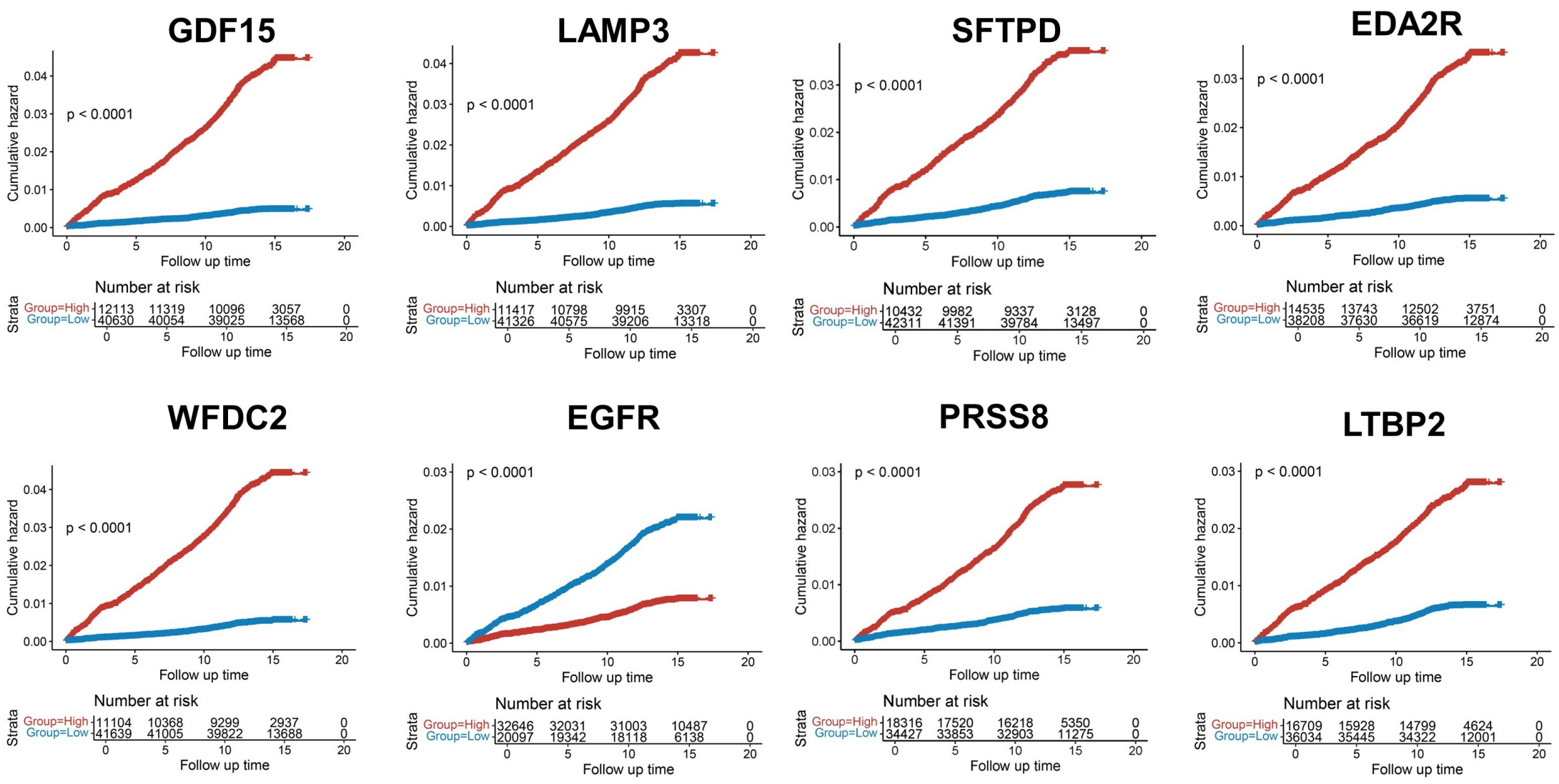

Supplementary Figure 7. Kaplan–Meier curves showing the cumulative incidence of ILD according to protein levels

The Kaplan-Meier curves demonstrate clinical progression over time for all incident ILD, visualizing individuals with low (blue lines) and high (red lines) baseline plasma levels of top 8 genes. The cut-off points for stratifying the high and low protein level groups were calculated by obtaining the maximum Youden index.

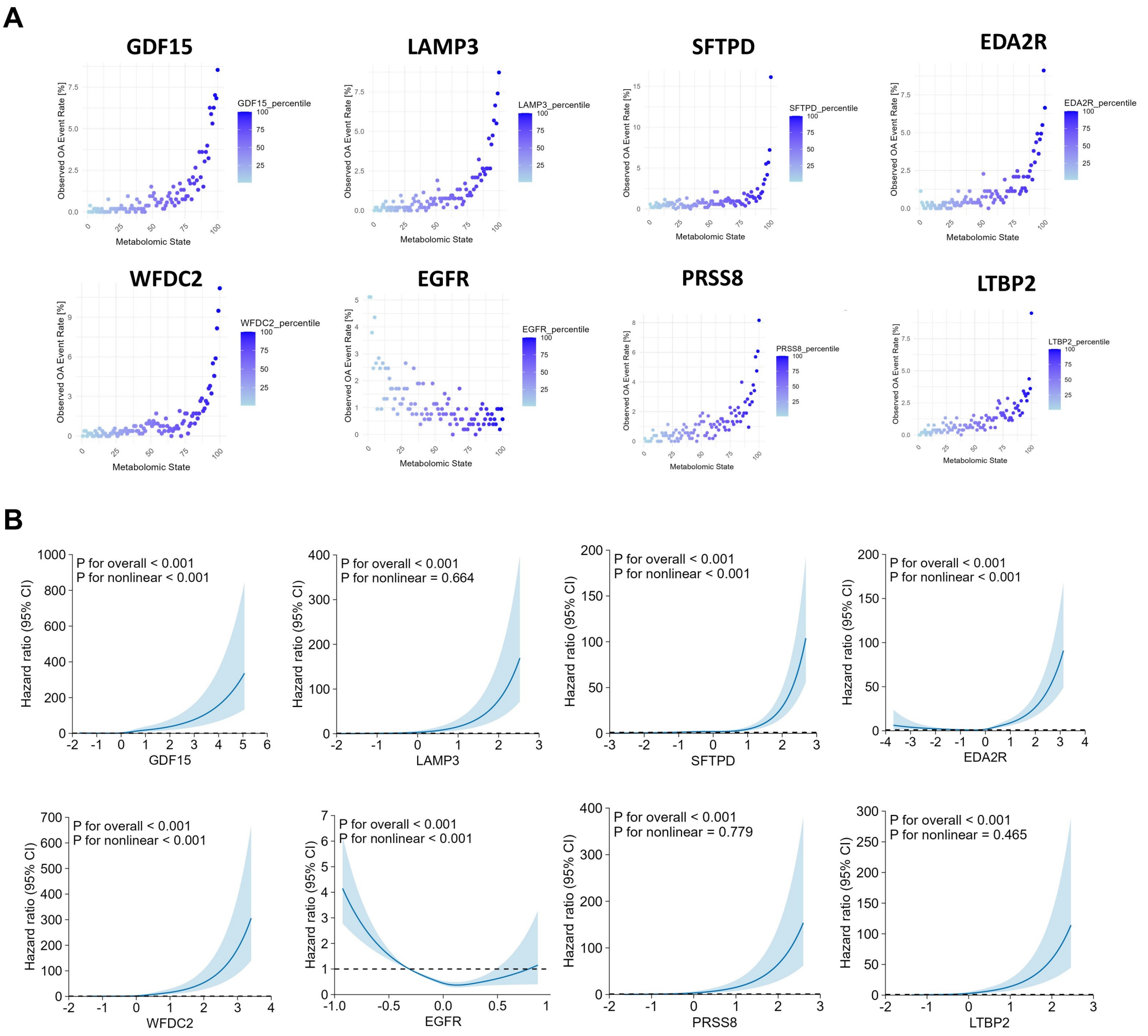

Supplementary Figure 8. Predictive performance of baseline protein levels on the risk of ILD

(A) Relationship between the observed event rate of incident ILD and baseline protein levels by percentile in the entire study population. The scatter plot illustrates the correlation between the observed event rate of new-onset ILD and the baseline levels of specific proteins, stratified by their percentile ranks within the entire study cohort.

(B). RCS curves illustrating the dose-response relationship between the expression levels of the top-ranked proteins and ILD risk. The x-axis represents the protein expression levels, and the y-axis represents the HR for ILD. The curves depict the linear/nonlinear relationship between protein expression and ILD risk, with confidence intervals shown as shaded areas.

Abbreviations ILD: Interstitial lung disease. RCS: Restricted cubic spline. HR: Hazard ratio.

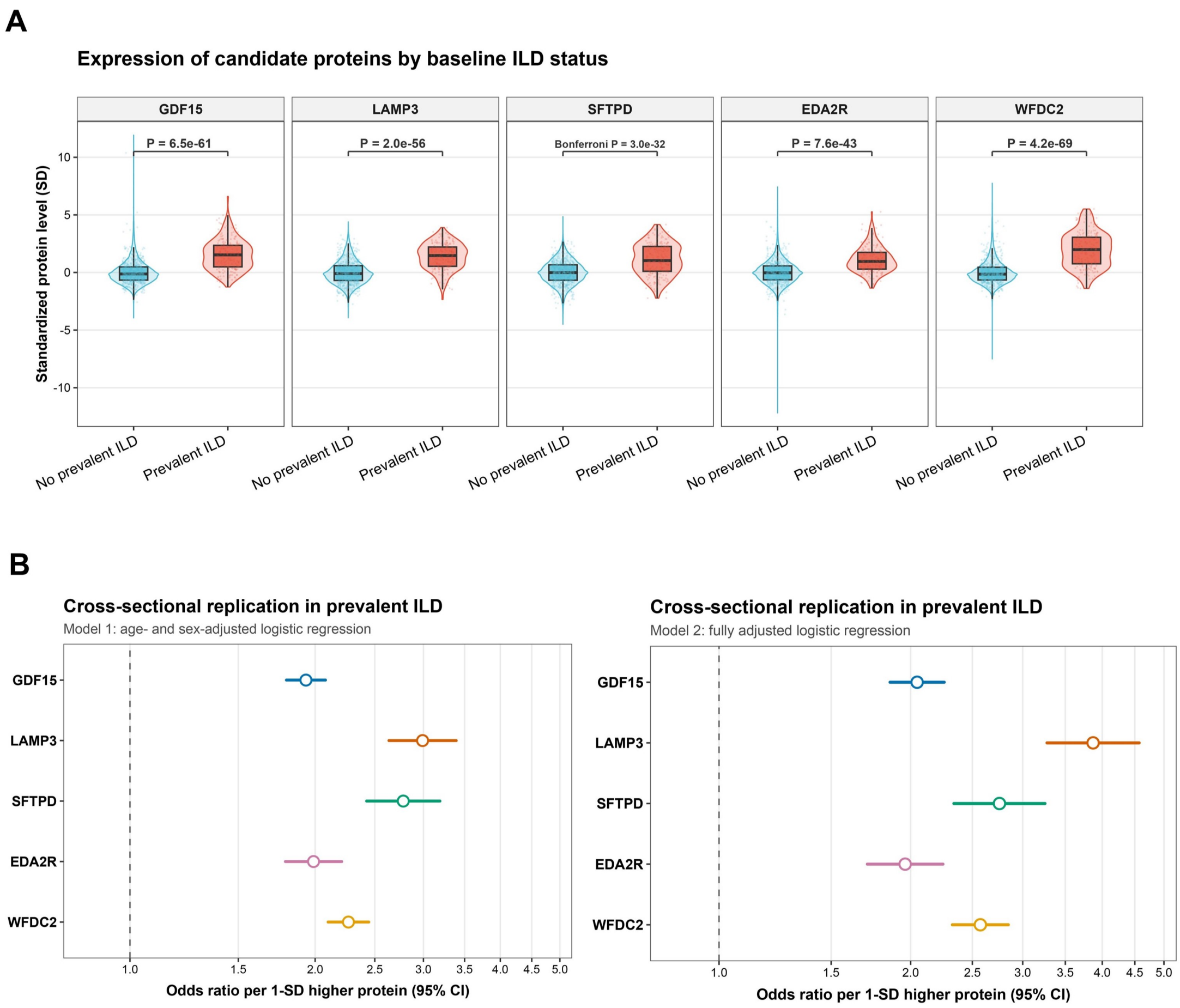

Supplementary Figure 9. Cross-sectional assessment of five candidate proteins in prevalent ILD

(A) Distributions of standardized plasma protein levels by baseline ILD status. Violin plots show distributions, with embedded boxplots indicating medians and interquartile ranges. Annotated P values indicate between-group comparisons.

(B) Associations between each protein and prevalent ILD from age- and sex-adjusted (Model 1) and fully adjusted (Model 2) logistic regression. Points and horizontal lines indicate odds ratios per 1-SD higher protein level and 95% confidence intervals, respectively. Dashed vertical lines indicate an odds ratio of 1. These analyses provide cross-sectional replication, not external or prospective validation.

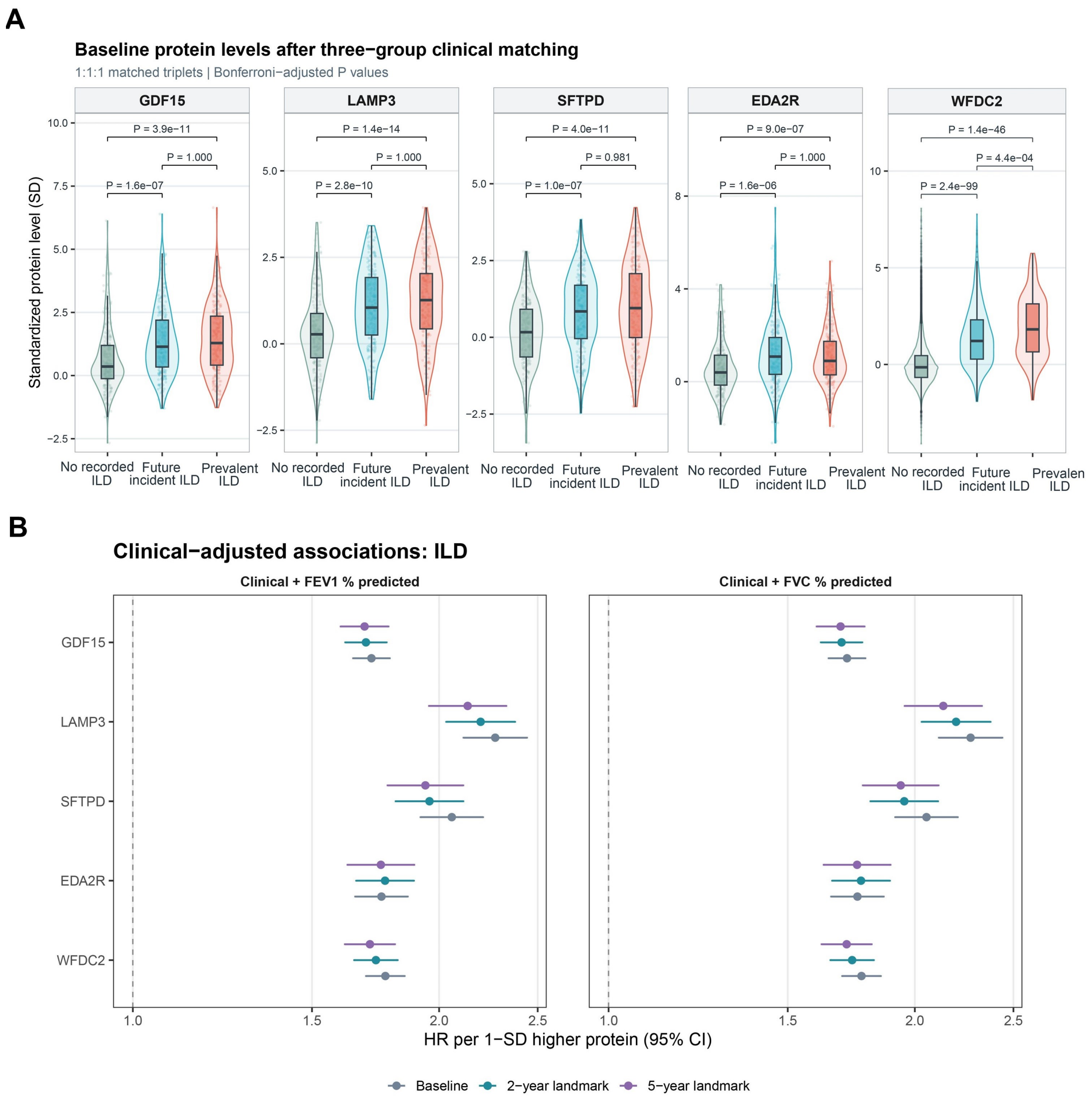

Supplementary Figure 10. Baseline protein levels across ILD groups and prospective associations in landmark analyses

(A) Baseline levels of GDF15, LAMP3, SFTPD, EDA2R and WFDC2 in 1:1:1 propensity-score-matched triplets comprising participants with prevalent ILD, future incident ILD and no recorded ILD during follow-up. Two binary matches sharing prevalent ILD participants as common anchors were intersected to construct triplets. Protein levels are expressed using a common reference SD scale. Violins show distributions; boxes indicate medians and interquartile ranges; whiskers extend to the furthest observations within 1.5 interquartile ranges. Brackets indicate within-triplet paired t-test P values with Bonferroni correction across 15 comparisons.

(B) Hazard ratios per 1-SD higher baseline protein level from clinically adjusted Cox models incorporating FEV1 or FVC percent predicted separately. Analyses used the baseline ILD-free cohort, rather than only the matched sample. The 2- and 5-year landmark analyses included participants remaining free of recorded ILD and under follow-up at each landmark, with follow-up restarted thereafter. Percent-predicted values were calculated using reference equations incorporating age, sex and height. Points and horizontal bars indicate hazard ratios and 95% confidence intervals; dashed lines indicate a hazard ratio of 1.

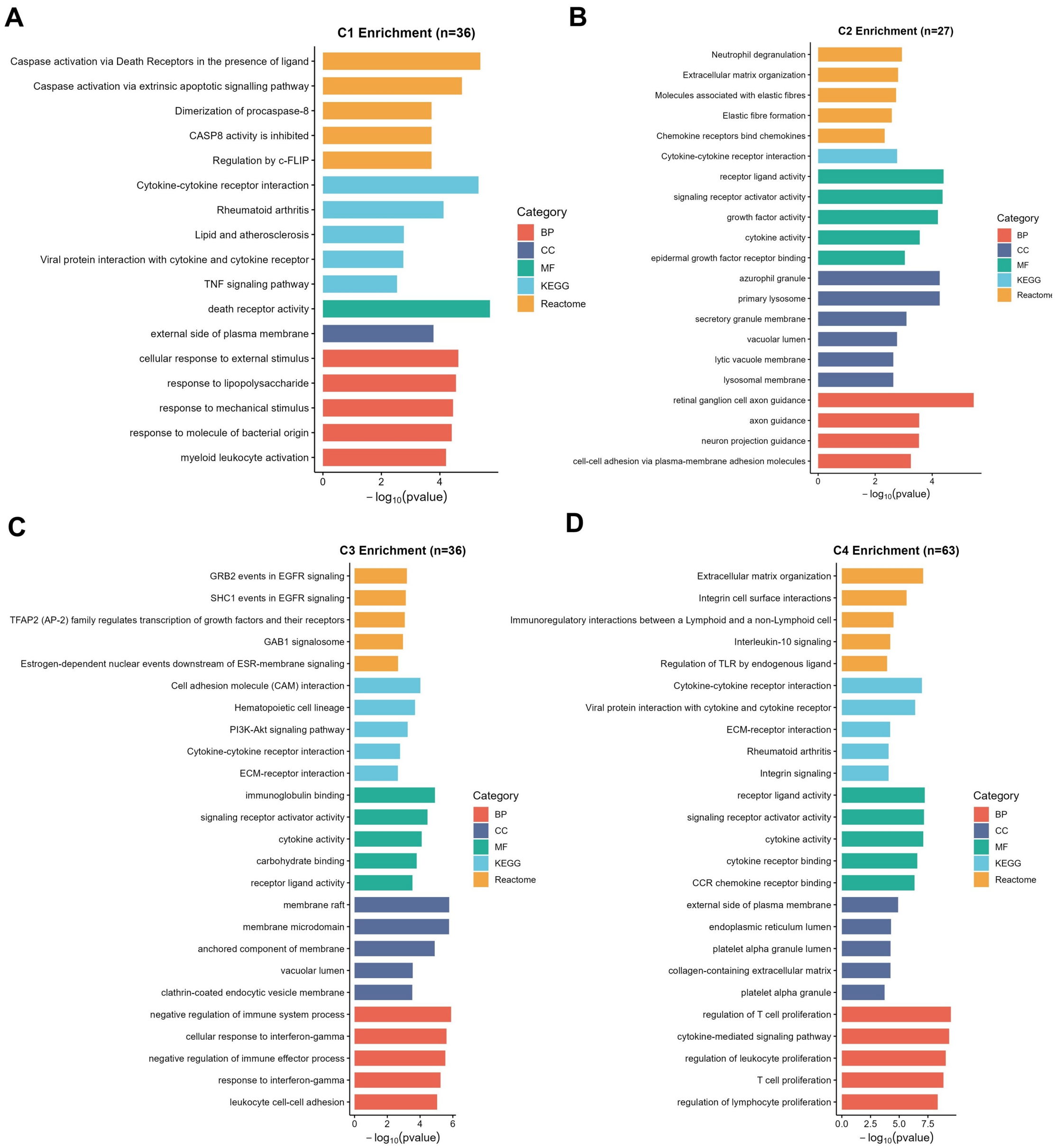

Supplementary Figure 11. Functional enrichment analysis of trajectory-based protein clusters

(A-D). Enrichment of GO, KEGG, and Reactome pathways for significant proteins identified in each trajectory cluster.

Abbreviations GO: Gene ontology; KEGG: Kyoto encyclopedia of genes and genomes.

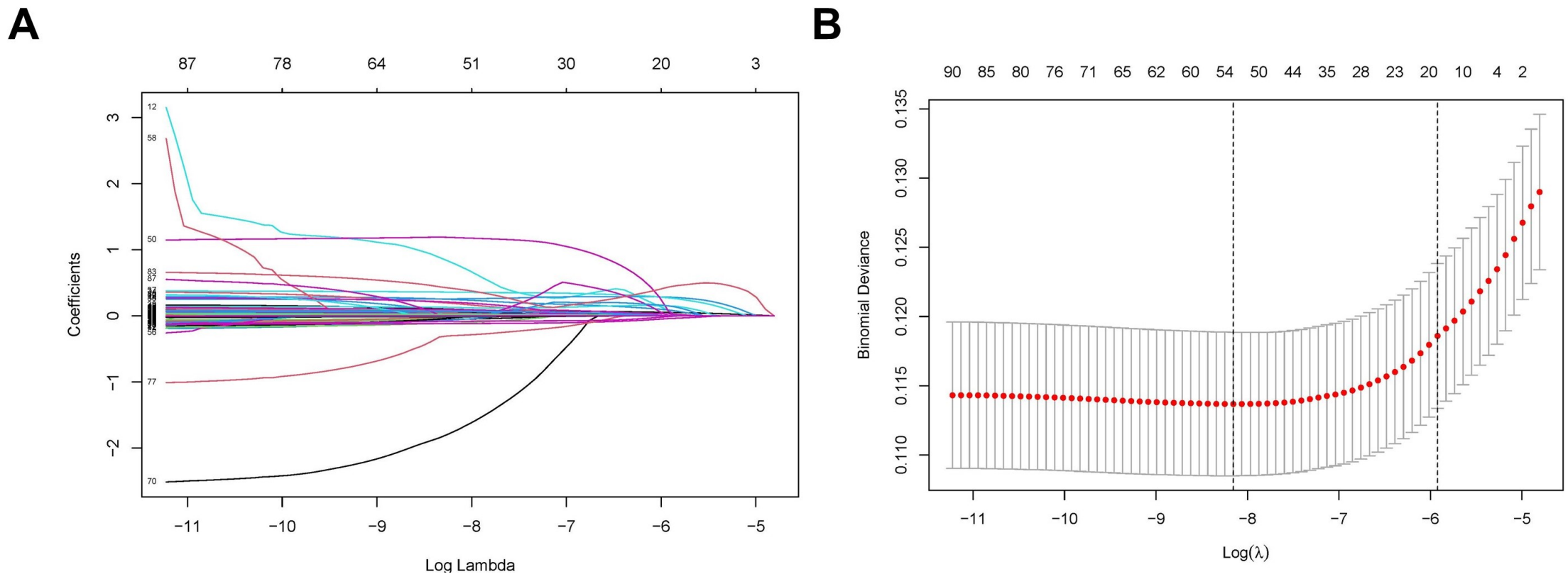

Supplementary Figure 12. LASSO (L1 regularization) clinical feature screening

(A) Coefficient path plot of the LASSO regression. The coefficient paths of the LASSO regression model for each predictor are shown as functions of the regularization parameter (lambda).

(B) Cross-validation curve of the LASSO regression. The line plot presents the mean cross-validated error of the LASSO regression model as a function of the regularization parameter (lambda). The vertical dashed line indicates the optimal lambda from 5-fold cross-validation.

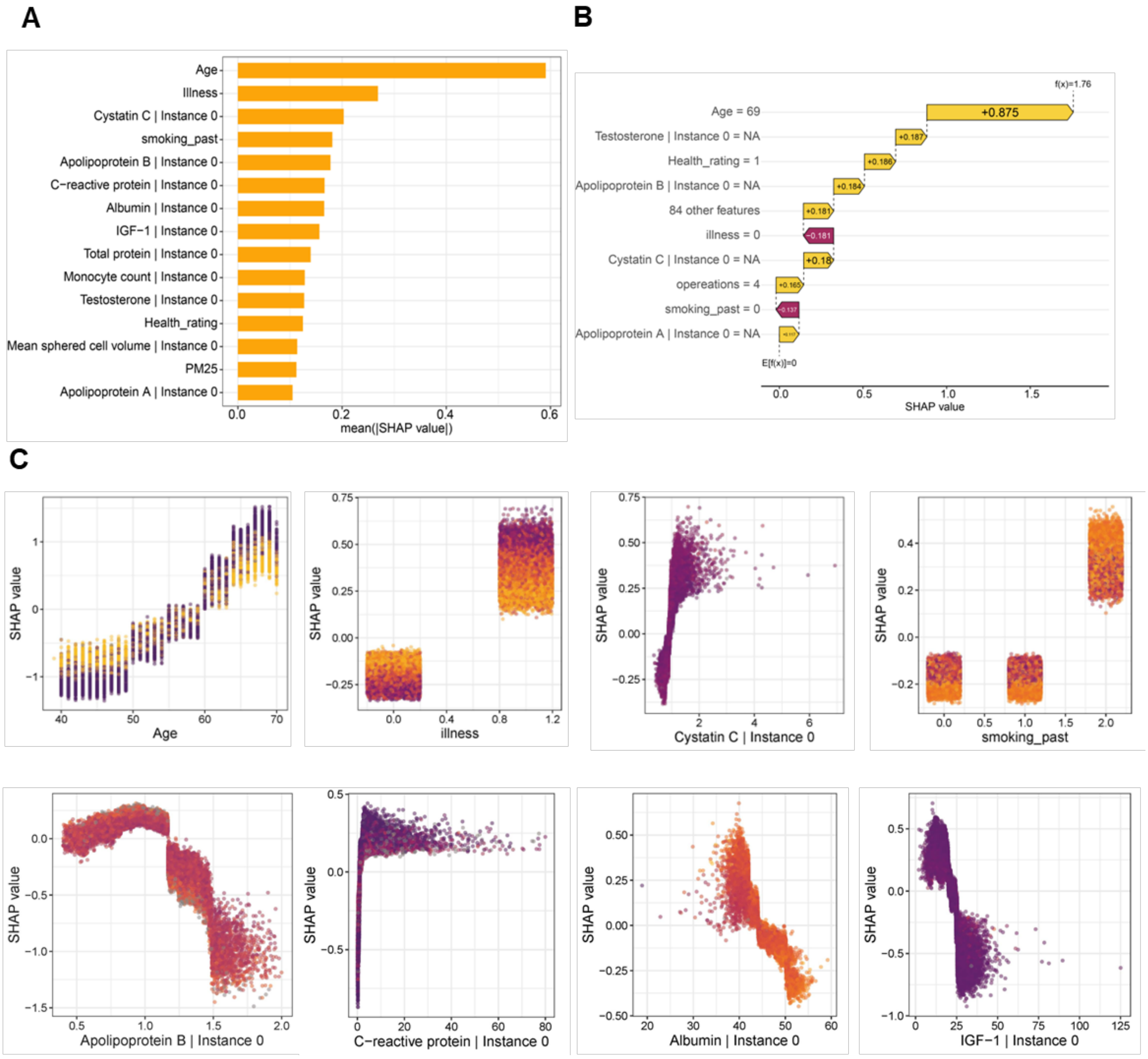

Supplementary Figure 13. Clinical model explanation by the SHAP method in ILD

(A) Summary bar plot of the SHAP.

(B) Waterfall plot showing the individual contributions of each feature to the risk of incident ILD. This waterfall plot shows the cumulative effects of individual features on the overall risk of developing ILD. Each bar in the plot represents a specific feature, with the height of the bar indicating the change in the risk score attributable to that feature. The plot starts with a baseline risk (indicated by the dashed line) and shows how the risk accumulates as each feature is added sequentially. The red bars represent the features that decrease the risk of ILD. The yellow bars represent the features that increase the risk of developing ILD. The final bar shows the total change in risk after considering all features.

(C) SHAP dependence plot. Each dependence plot shows the effect of a single feature on the output of the prediction model, where each dot represents a patient.

Abbreviations SHAP: SHapley Additive exPlanations. ILD: Interstitial lung disease.

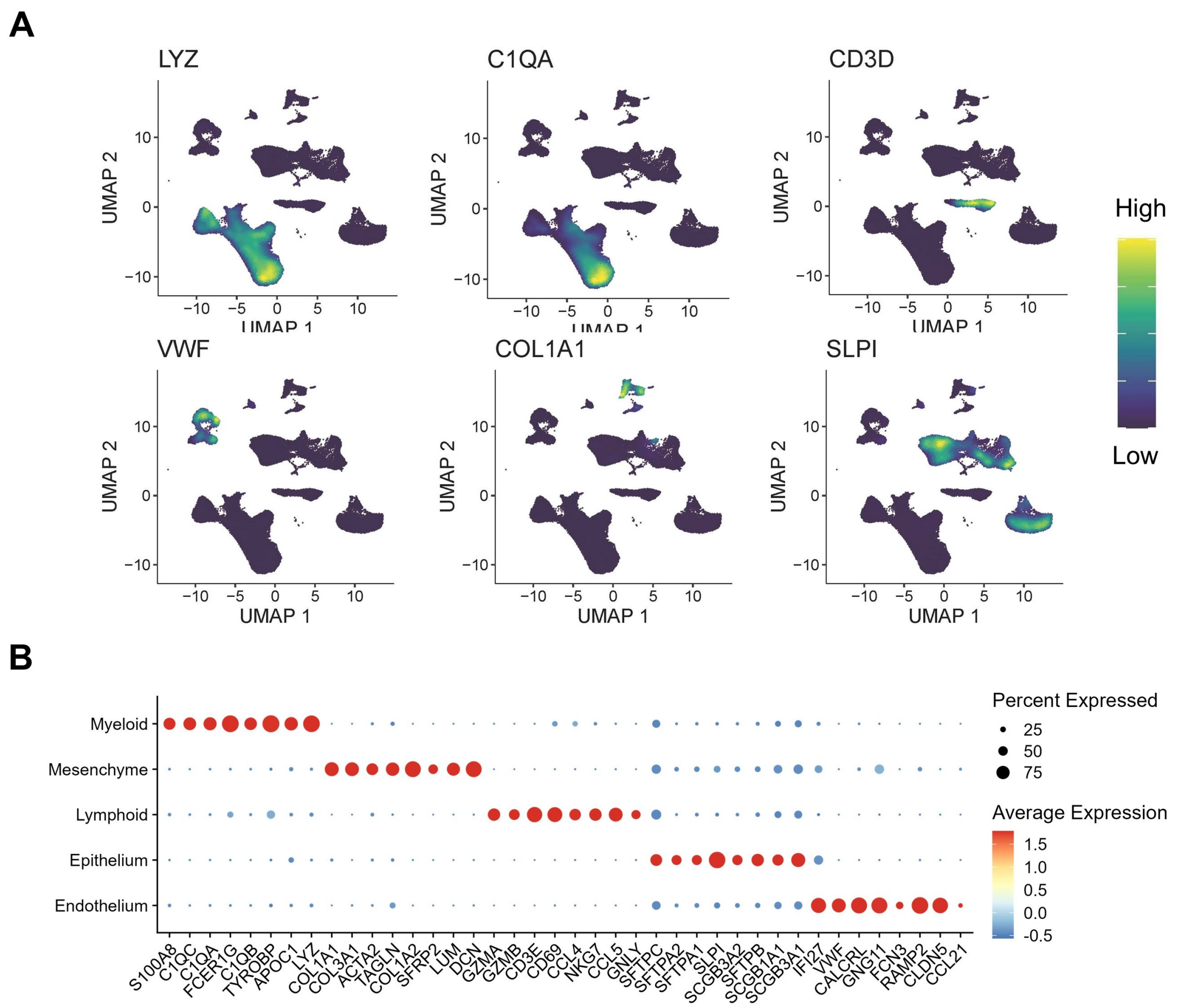

Supplementary Figure 14. Marker gene expression in the single-cell RNA sequencing data from idiopathic pulmonary fibrosis (IPF) lungs.

(A) UMAP plot showing the expression levels of canonical marker genes for each major cell type. The expression levels were overlaid on the cell clusters.

(B) Dot plot showing the expression patterns of characteristic genes across annotated cell types. The size of each dot corresponds to the percentage of cells within a cluster that express the gene, and the color intensity represents the average expression level in the expressing cells.
